# Agentic campaign control for high-throughput de novo binder design

**DOI:** 10.64898/2026.09.22.753604

**Published:** 2026-09-24

**Authors:** Minkyu Jeon, Jinyeop Song, Jina Kim, Ellen D. Zhong

**Affiliations:** 1 Department of Computer Science, Princeton University; 2 Department of Physics, Massachusetts Institute of Technology

## Abstract

Progress in artificial intelligence has produced a rapidly growing ecosystem of methods for *de novo* protein design. With access to many specialized and often complementary tools, how does one use them effectively, especially with a finite compute budget? Here, we introduce **Target-adaptive Rescue–Explore–eXploit (T-REX)**, an agentic protein binder design framework that orchestrates multiple state-of-the-art protein generative models and structure evaluators. Over the course of a design campaign, T-REX leverages large language model (LLM) agents to reason over accumulating outcomes and decide whether to rescue promising candidates, explore alternative settings, or exploit productive routes, while a deterministic controller validates and schedules proposed actions. Across seven targets, we find that T-REX adaptively allocates its compute across methods in a target-dependent manner and achieves the highest throughput of structurally distinct hits compared to all baselines. These results suggest that adaptive orchestration can complement increasingly powerful protein design models, and we openly release T-REX to support compute-efficient binder design workflows.

## 1. Introduction

De novo protein design is emerging as a powerful paradigm to create novel proteins of desired structures and molecular functions, with opportunities in therapeutics, catalysis and molecular engineering (Chu et al., 2024; Listov et al., 2024; Bennett et al., 2023; Watson et al., 2023). Recent advances in artificial intelligence have introduced a growing suite of computational tools that are revolutionizing various stages of the protein design pipeline (Yang et al., 2026), including generative models of backbone structures (Watson et al., 2023; Ingraham et al., 2023), inverse folding for sequence design (Ingraham et al., 2019; Dauparas et al., 2022; Hsu et al., 2022), and folding models for structure prediction and evaluation (Jumper et al., 2021; Lin et al., 2023; Abramson et al., 2024; Chai Discovery et al., 2024; Wohlwend et al., 2025; Protenix Team et al., 2026). Joint structure–sequence generative models (Stark et al., 2025; Didi et al., 2026b; Hayes et al., 2025) and integrated hallucinate-and-filter pipelines such as BindCraft (Pacesa et al., 2025) provide yet additional paths to contemporary *de novo* protein binder design.

As the set of individual tools becomes increasingly capable and diverse, a new question emerges: *which methods should be run, with which settings, and how should a finite compute budget be allocated*, especially as the protein design campaign progresses? Here, a campaign denotes the sequence of computational generation, redesign and evaluation jobs carried out for a single target. Notably, these specialized tools can differ in productivity and computational cost (Liu et al., 2026), both between targets and throughout the course of a design campaign. At different stages, candidate designs can miss *in silico* metrics or duplicate existing structures, and tool calls can fail. Additionally, designs that do not initially qualify can still provide promising starting points for subsequent structural or sequence refinement (Vázquez Torres et al., 2024; Bennett et al., 2023). Effective orchestration of available methods therefore requires interpreting these outcomes to decide whether to refine promising designs, continue productive approaches, or test alternative paths.

Recent progress in scientific agents suggests that large language models (LLMs) can provide the contextual reasoning capabilities needed to interpret heterogeneous outcomes and autonomously strategize follow-up actions (Ding et al., 2025; Ghafarollahi and Buehler, 2024; Ouyang et al., 2026; Swanson et al., 2025; Teneggi et al., 2026; Xu et al., 2026). In protein design, LLM agents have begun to coordinate multi-stage computational workflows and iterative design procedures (Ge et al., 2026; Swanson et al., 2025), and recent approaches have shown autonomous protein design albeit with large compute and LLM token budgets (Claude Science and Shanehsazzadeh, 2026). A central control challenge remains in adaptively allocating limited compute over the course of a design campaign. In particular, protein generation, redesign and structure evaluation are computationally intensive, whereas LLM reasoning over compact summaries of campaign outcomes can be comparatively fast. A relatively brief reasoning step can therefore guide more expensive computation by specialized scientific tools. Concurrent jobs also finish at different times, providing new evidence while other computations remain in progress. These differences in computational cost and runtime motivate a supervisory campaign controller that continually interprets incoming evidence and decides which methods and settings to run next while other jobs continue asynchronously.

Here, we introduce **Target-adaptive Rescue–Explore–eXploit (T-REX)**, an agentic campaign controller for high-throughput de novo binder design. In T-REX, LLM agents interpret accumulating campaign evidence to propose and prioritize work according to three strategies: **Rescue**, which directs follow-up computation toward an identified weakness in a promising design or route; **Explore**, which tests underused methods or alternative configurations; and **eXploit**, which allocates additional computation to productive routes. A deterministic execution layer then validates the proposed jobs, enforces resource constraints, and manages asynchronous execution. Across seven de novo binder-design targets, T-REX achieved the highest throughput of structurally distinct hits on every target, outperforming a non-agentic tree-search controller by a geometric mean of 2.43-fold and the strongest generator-only baseline for each target by 2.48-fold under matched compute budgets. Analysis of the campaign records shows that T-REX uses different follow-up jobs in response to distinct failure modes and shifts its **Rescue-Explore-eXploit** priorities as campaign conditions change. These results support evidence-guided, adaptive orchestration as a practical approach to allocating compute across complementary protein-design methods during a design campaign. T-REX is available fully open source at https://github.com/ml-struct-bio/T-REX.

## 2. Results

### 2.1. Overview of the T-REX campaign controller

T-REX is designed to adaptively orchestrate diverse computational protein binder design tools to maximize structurally distinct hits under a fixed compute budget (Figure 1). The toolset includes six generation tools – four Proteína-Complexa variants (beam search, best-of-*n*, Feynman-Kac steering, and Monte Carlo Tree Search (MCTS)), BindCraft, and BoltzGen (Stark et al., 2025; Didi et al., 2026b; Pacesa et al., 2025) – along with ProteinMPNN (Dauparas et al., 2022) for sequence redesign and AlphaFold2 (AF2) refolding for structure evaluation (Jumper et al., 2021) (Figure 1a). We formulate a *de novo* binder-design campaign as an online allocation problem over computational jobs where each job specifies an action family with a particular tool, hyperparameter configuration, and, when applicable, a previously generated parent design (Supplementary Table S5). Designed binders must satisfy pre-specified *in silico* metrics to qualify as hits (e.g., pLDDT, iPAE, scRMSD thresholds), and the accumulating pool of designed binders is structurally clustered by Foldseek (Van Kempen et al., 2024) to count unique hits (Methods Section 4.2).

During a campaign, hit production can slow or stall, but simply considering a scalar hit count does not reveal where progress is being lost or what type of follow-up is appropriate. In T-REX, we distinguish four operational failure modes (Figure 1b). These correspond to different points where ongoing or completed work has not yet produced a new structural hit: generation may produce no usable output, evaluation may still be pending, a candidate may narrowly miss the hit qualification criteria, or a qualified design may duplicate a structure already found. Because these failure modes arise for different reasons, they can require different responses. To organize these responses, T-REX defines three priorities for follow-up actions: **Rescue**, **Explore**, and **eXploit** (Figure 1c). **Rescue** addresses an observed limitation in a promising design or route, **Explore** tests alternative methods or settings, and **eXploit** allocates further compute to routes that continue to produce new structural hits.

Rather than relying only on a scalar hit count, T-REX uses a structured summary of outcomes (i.e. the *EvidenceSummary*) to represent the broader campaign context (Figure 1d). The *EvidenceSummary* combines completed results, ongoing work, and available resources, summarizing evidence about structure quality and novelty, route-specific yield and cost, pending evaluations, design lineage, and active compute (Figure 1d; Methods Section 4.3; Supplementary Section C.4 and Supplementary Figure S1). The *EvidenceSummary* is used to assign one of seven campaign states, which provide a high-level description of the campaign’s current condition, such as whether evidence is still sparse, new structures are being produced, near misses are accumulating, or progress has stalled (Figure 1d and Supplementary Section C.4.5). Because the relevance of the evidence depends on the current campaign state and history, choosing what to try next requires contextual interpretation. T-REX therefore uses LLM agents to reason over the campaign state, underlying evidence, and history. The Planner LLM formulates new evidence-linked hypotheses and follow-up tests as *HypothesisCards*. The proposed follow-up jobs then pass a deterministic “Check + Build” stage for validation. Finally, the Supervisor LLM assigns each valid candidate job to **Rescue**, **Explore**, or **eXploit**, ranks candidate jobs, and proposes an R/E/X allocation specifying the desired proportions of subsequent jobs devoted to each priority (Figure 1d). The deterministic controller uses these priorities and rankings to select jobs for execution as worker capacity becomes available, subject to feasibility and resource constraints.

Generation, redesign, and evaluation jobs can differ substantially in runtime and resource requirements, so waiting for all ongoing work to finish before planning the next job would delay the use of newly available evidence from a recently completed job. T-REX therefore uses asynchronous execution to incorporate completed results into decisions about which methods and settings to run next (Figure 1e). When a job finishes and its GPU becomes available, the controller can assign a new proposed job to that GPU while jobs on other GPUs continue. As each job finishes, its results are added to the campaign archive, a JSON-style record of campaign results and decisions (Methods Section 4.3). These results are summarized in the *EvidenceSummary*, which is used primarily as evidence for deciding which job to run next. This closes the loop between execution and evidence-guided planning (Figure 1e; Methods Section 4.5). In this work, the Planner and Supervisor use Qwen3.6–27B-FP8, served with vLLM (Team, 2026; Kwon et al., 2023).

### 2.2. T-REX achieves high-throughput in silico design across diverse protein targets

We compare T-REX against three generator-only baselines and two non-agentic adaptive controllers under matched worker-compute budgets across seven targets selected from the Proteína-Complexa (Complexa) (Didi et al., 2026a) benchmark: CD45, BetV1, CbAgo, HER2-AAV, SC2RBD, PDL1 and IL7RA (Figure 2; Table 1). The generator-only baselines were Complexa, BindCraft (Pacesa et al., 2025) and BoltzGen (Stark et al., 2025); the adaptive comparators were non-agentic tree-search controller PUCT (Aygün et al., 2026; Silver et al., 2017; Rosin, 2011) and *ϵ*-greedy (Vermorel and Mohri, 2005). PUCT and *ϵ*-greedy operated over the same action space and execution and evaluation framework as T-REX, but used scalar reward-based allocation without LLM-based contextual reasoning (Supplementary Section B.5.1).

Across the seven targets, T-REX produced 1,461 structurally unique hits (SUs) clustered at a TM threshold of 0.6 with each design campaign spanning 48 hours on a 4-H100 GPU node (144 worker GPU-hours). The final SU count of T-REX for each target exceeded all five baselines (Figure 2a and Table 1). No single generator-only method was strongest across all targets, supporting the use of target-adaptive tool orchestration. T-REX throughput was 1.38–4.36-fold that of PUCT across targets, with geometric-mean gains of 2.43-fold over PUCT and 2.48-fold over the strongest observed generator-only baseline for each target (Figure 2a and Table 1). Comparisons at 10, 50, 100 and 144 GPU-hours further quantify the throughput trajectories in Figure 2a (Supplementary Section D.1.2 and Supplementary Table S10).

The throughput gain of T-REX was robust to alternative definitions of structural diversity and design quality (Figure 2b, c). T-REX led on every target under structural clustering at TM-score thresholds of 0.5, 0.6 and 0.8 and sequence clustering at 70% identity (Figure 2b and Supplementary Tables S14 and S13). The throughput gain was also retained as hit qualification criteria were tightened (Supplementary Figure S4). Across AF2, actifpTM, PRODIGY, ESMFold2 and Boltz-2 diagnostics, T-REX retained the largest total number of structurally distinct hits passing each score threshold (Figure 2c and Supplementary Figure S6). This indicates that the throughput gain of T-REX is not limited to AF2-based assessment.

We ran independently initialized replicate campaigns for three targets (CD45, SC2RBD, and CbAgo) and observed that T-REX consistently outperformed both PUCT and *ϵ*-greedy baselines (Figure 2d). T-REX also had higher throughput than a no-LLM SMAC3 Bayesian optimizer on all seven targets, extending the comparison beyond PUCT and *ϵ*-greedy (Supplementary Table S11) (Lindauer et al., 2022). Together, these analyses show that the throughput gain was not specific to the primary diversity or quality criteria, a single controller initialization, or the particular adaptive comparators used in the primary benchmark.

### 2.3. T-REX integrates campaign evidence to propose follow-up jobs

To qualitatively investigate how T-REX uses campaign context to interpret failures and select follow-up jobs, we retrospectively examined three illustrative campaign trajectories. In HER2-AAV, a pLDDT-reward test yielded no qualified designs (0/32), leading to a revised hypothesis about sequence–backbone compatibility and a sequence refinement job that yielded 5/32 qualified designs (Figure 3a). In CbAgo, five designs with iPAE values above the cutoff motivated broader Feynman–Kac (FK) steering (Singhal et al., 2025; Didi et al., 2026b), which yielded 4/16 qualified designs (Figure 3b). In PDL1, a parent that failed only pLDDT was selected for fixed-backbone ProteinMPNN redesign, yielding 14/16 qualified descendants and one new SU (Figure 3c). For each case, the campaign archive links the earlier evidence to the proposed follow-up test, confirmed job start, and resulting measurements; the PDL1 example additionally retains the direct parent–descendant relationship (Supplementary Section D.2.3). These examples illustrate how T-REX reused criterion- and route-specific evidence to propose different follow-up computations.

### 2.4. T-REX adapts allocation across campaign states and prioritizes stall recovery

We next investigated how T-REX adapts its allocation by examining shifts in **Rescue**, **Explore**, and **eXploit** priorities across campaign states. Campaign states were assigned from archived campaign evidence using our seven-state classification (Figure 1d and Methods Section 4.3). Among 1,454 confirmed generation and redesign starts, **Explore** accounted for 74% of starts when little evidence was available, **Rescue** for 51% during “stall” or “deep stall”, and **eXploit** for 82% in “productive-with-duplication” states (Figure 4a). Thus, T-REX allocated work differently across campaign states, favoring **Explore** when evidence was sparse, **Rescue** during stalls and **eXploit** when productive routes continued to yield new structures despite frequent structural duplication.

We tested the role of **Rescue** prioritization in stall recovery by removing this priority while retaining the same action space. Over the first 100 GPU-hours, recovery was measured from entry into a stall or deep-stall campaign state until the next new TM 0.6 SU (Figure 4b; Supplementary Section D.3.3). Full T-REX recovered in 26 of 27 such entries, compared with 14 of 20 under the Rescue removed intervention. Among entries followed by recovery, the median time to the next SU was shorter with full T-REX for both “stall” (1.77 versus 5.04 GPU-hours) and “deep stall” (3.52 versus 7.60 GPU-hours); full T-REX also spent less time in “stall” or “deep-stall” states than the Rescue removed intervention (3.8% versus 27.7%). These results support a role for **Rescue** prioritization in recovering from stalled campaign states.

Finally, we examined how different job outcomes and structural duplication were associated with allocation priorities and follow-up jobs. Among 1,305 confirmed generation and redesign starts with a retrospectively identified primary evidence focus, interface confidence (iPAE) and structural duplication were most often associated with **eXploit** (65% and 93%, respectively), while fold confidence (pLDDT) was most often associated with **Rescue** (88%; Figure 4c). Follow-up jobs also differed by evidence: confidence-related evidence commonly led to changes in generator scoring weights or sequence redesign, while structural duplication was most often associated with repeating known routes, followed by changes in sampling or search settings (Figure 4d; Supplementary Section D.3.2).

### 2.5. Designed proteins span diverse secondary structures and predicted binding positions

To qualitatively characterize the repertoire of binder structures produced by T-REX, we examined the secondary-structure composition of the structurally distinct hits. Across the seven primary target campaigns, we calculated *α*-helix and *β*-strand fractions for all 1,461 final TM 0.6 representatives using simplified three-state Dictionary of Protein Secondary Structure (DSSP) assignments (Kabsch and Sander, 1983; McGibbon et al., 2015). Representative *α*-helix-rich, mixed helix/strand and *β*-strand-rich binders are shown in Figure 5a, with their secondary-structure composition shown among all representatives in Figure 5b. To compare *β*-strand-rich designs across methods, we selected all qualified designs with at least 20% of binder residues assigned as a *β*-strand before structural clustering. Across the seven targets, T-REX yielded 308 TM 0.6 clusters, compared with 95 for BoltzGen-only, the highest count among the other methods (Supplementary Table S23). These results show that structurally distinct hits produced by T-REX span different secondary-structure compositions, including more *β*-rich clusters than the other methods tested.

We then analyzed predicted target-relative binder positions for CbAgo to assess structural variation around the target. After aligning each predicted complex on the target, we obtained each binder center as the mean position of its C*α* atoms. Joint principal component analysis (PCA) showed that T-REX binder centers occupied a broader region than those from PUCT (Figure 5c). Across 1,000 sample-size-matched resamples of 30 representatives per method, the median pairwise separation between binder centers averaged 31.2 Å for T-REX and 9.97 Å for PUCT. Related interface-contact analyses for all seven targets are reported in Supplementary Table S24. Together, these results show additional structural variation among T-REX hits, including different secondary-structure compositions and, for CbAgo, a broad range of predicted target-relative binder positions.

## 3. Discussion

We propose **Target-adaptive Rescue–Explore–eXploit (T-REX)**, an agentic campaign controller that achieves high-throughput *de novo* binder design by orchestrating multiple specialized protein structure generators and evaluators. By leveraging multiple design tools and strategies, T-REX achieved higher hit throughput than both single-generator baselines and non-agentic adaptive controllers under matched compute budgets (Figure 2). These results highlight how campaign-level orchestration is emerging as a complementary direction to continued improvements in specialized protein-design tools, consistent with general trends towards higher-level abstractions for generative design in biology (Merchant et al., 2026).

Central to T-REX is its combination of adaptive decision-making driven by LLM reasoning, a structured classification of outcomes and failure modes tailored to protein-design campaigns, and deterministic control for efficient resource allocation. Rather than asking an LLM to reason directly over an unstructured history of design attempts, T-REX organizes campaign outcomes into interpretable failure modes and campaign states that provide context for subsequent decisions (Figure 1). Two LLM agents then operate over this structured evidence: a Planner proposes evidence-linked hypotheses and follow-up actions, while a Supervisor prioritizes candidate actions and proposes how subsequent computation should be distributed among **Rescue**, **Explore**, and **eXploit**. These priorities distinguish between repairing promising but unsuccessful designs or routes, exploring alternative strategies, and extending strategies that are already productive (Figures 3,4). Together, these design choices provide structure to autonomous decision-making while retaining the flexibility of LLM reasoning.

Here, we solely evaluated T-REX in a computational setting using established *in silico* metrics to score “successful” and “unique” hits. While experimental validation is ulimtely required, we note that the individual generators within T-REX (Proteína-Complexa, BindCraft and BoltzGen) have each produced experimentally validated binders in their original studies (Didi et al., 2026a; Pacesa et al., 2025; Stark et al., 2025). Real-world *de novo* design and development also require assessment of binding affinity (Passaro et al., 2025; Xue et al., 2016), specificity and developability (Listov et al., 2024), followed by iterative lead optimization, molecular simulation and mutation screening (Barletta et al., 2024; Chaudhury et al., 2010). Extending T-REX to these stages would require adding the corresponding computational tools and experimental operations to the action space and adapting the *EvidenceSummary*, campaign states, decision criteria, and deterministic constraints to their outcomes and costs. Emerging techniques in robotics and autonomous laboratories could further connect this control loop to experimental validation, enabling cycles of design, testing and refinement (Boiko et al., 2023).

More generally, T-REX complements recent scientific-agent systems, including *Autoresearch* and *The AI Scientist*, that use feedback to guide extended sequences of computational experiments (Karpathy, 2026; Lu et al., 2026; Yamada et al., 2025). A potentially transferable design principle is the separation of structured campaign-state representation, LLM-based proposal and prioritization, and deterministic validation and execution for allocating limited resources. Future work could examine whether these principles can improve resource allocation in other scientific campaigns involving heterogeneous tools, delayed outcomes, and fixed budgets, using domain-specific campaign states and Rescue, Explore, and eXploit priorities. To facilitate adoption and further development, we openly release T-REX together with its agent prompts and campaign-control framework. T-REX uses Qwen3.6–27B-FP8, an openly available LLM that can be served locally, and is available at https://github.com/ml-struct-bio/T-REX.

## 4. Methods

### 4.1. Campaign specification and shared action space

#### Campaign setup and controllers.

T-REX adaptively selects computational jobs to maximize the number of structurally distinct binder-design hits obtained within a fixed compute budget. A campaign comprises generation, redesign, and evaluation jobs carried out for a single target using a fixed compute budget and multiple GPUs. For each target, campaigns used a prespecified target structure and binding-site hotspots, along with a default binder-length range (Supplementary Table S1). Structurally distinct hits were counted using the common in silico qualification criteria and structural clustering described in Section 4.2. T-REX, PUCT (Aygün et al., 2026; Silver et al., 2017; Rosin, 2011) and *ϵ*-greedy (Vermorel and Mohri, 2005) served as campaign-level controllers and operated over the same action space, with shared parameter bounds and a common execution and evaluation framework. PUCT and *ϵ*-greedy used scalar reward-based allocation without using LLM-based contextual reasoning (Supplementary Section B.5.1).

#### Shared action space.

The shared action space, defined as the action families and configurations permitted within a campaign, comprised eight action families: de novo generation using four Complexa search variants—beam search, best-of-*n*, Feynman–Kac (FK) steering and Monte Carlo tree search (MCTS)—as well as BindCraft and BoltzGen; sequence redesign using ProteinMPNN; and evaluation using AF2 (Singhal et al., 2025; Didi et al., 2026b; Pacesa et al., 2025; Stark et al., 2025; Dauparas et al., 2022). Within this action space, an action family identifies a computational method, a route groups generation or redesign jobs that share an action family and configuration, and a job specifies the concrete computation to execute, including its permitted settings and, when applicable, an eligible parent design. Controllers could adjust family-specific configuration settings and the weights of Complexa reward terms or BindCraft design loss terms within prespecified bounds. The pretrained model weights, generator implementations, and permitted action space remained fixed throughout each campaign, while available jobs changed as controllers selected permitted configurations and newly generated designs became eligible for redesign.

#### Campaign execution.

All three adaptive controllers began with prespecified Complexa Beam, BoltzGen and BindCraft jobs using common initial settings. PUCT and *ϵ*-greedy additionally started a Complexa best-of-*n* job on their fourth worker. As results became available, subsequent job selections used the accumulated campaign evidence, including job and evaluation outcomes, design quality and novelty, route productivity and cost, and resource state (Section 4.3, Supplementary Section C.4). Worker use was counted per job and qualification per design. For controller statistics, routes served as the unit for aggregating job outcomes and compute use. Redesign routes retained the upstream generation context. Operationally, each campaign followed a repeated evidence, planning, and execution loop: completed jobs updated campaign evidence; the controller used that evidence to propose and prioritize follow-up work; validated jobs were admitted to the shared worker queue; and their outcomes became evidence for subsequent decisions. Sections 4.3–4.5 describe each stage of this loop.

### 4.2. Design qualification and throughput measurement

#### Design qualification.

Designs were evaluated in two stages: they first had to satisfy the common qualification criteria, after which qualified binder chains were clustered to identify structurally distinct hits (SUs). Designs qualified only if binder pLDDT ≥ 90, normalized iPAE ≤ 7/31 and binder scRMSD < 1.5 Å, using thresholds adapted from the Proteina-Complexa evaluation protocol (Didi et al., 2026b) and measurements from the common ColabDesign-derived AF2-Multimer protocol (Didi et al., 2026b; Evans et al., 2021; Ovchinnikov et al., 2025). Normalized iPAE was the mean of the two directional binder-to-target and target-to-binder PAE means, divided by 31. Binder scRMSD was the C*α* root-mean-square deviation between generated and AF2-predicted binder structures after Kabsch alignment of the corresponding target C*α* atoms (Kabsch, 1976). Complexa normally produced all three qualification measurements during generation. Outputs from BindCraft, BoltzGen, and ProteinMPNN redesign generally required separate standardized AF2 evaluation, subject to the admission rules described in Section 4.5. Generator-native scores (e.g., BoltzGen design ipTM, interface ipTM, structure confidence and native-refold RMSD) and labels (e.g., BindCraft accept/reject labels indicating whether a design passed BindCraft’s native filters) could guide planning but did not determine qualification.

#### Structural novelty.

A qualified design could still add no new SU if its binder chain clustered with a structure already represented in the campaign. Foldseek clustered qualified binder chains separately within each campaign at TM0.6, a threshold previously used with Foldseek to quantify structural diversity in generated protein backbones (Jendrusch and Korbel, 2025). TM0.5 and TM0.8 were additionally evaluated as sensitivity analyses (Supplementary Table S14). Each final cluster counted as one structure-unique unit (SU) (Van Kempen et al., 2024), quantifying structural similarity and not biological fold or epitope identity. Protocol and clustering details are given in Supplementary Sections B.1–B.2 and Table S4.

#### Throughput measurement.

Final SU counts for campaigns controlled adaptively by T-REX, PUCT or *ϵ*-greedy were recomputed from the finalized campaign records rather than copied from the last *EvidenceSummary* generated during campaign execution (see Section 4.3 for details on the *EvidenceSummary*). Each SU was credited to the generation or redesign job that produced its selected representative, not to an evaluation job; rules for selecting one representative design from each cluster are given in Supplementary Section B.2. Throughput is defined as *N*_SU_/*H*, where *N*_SU_ is the final cluster count and *H* = 144 H100 worker GPU-hours for each method–target combination in the primary benchmark. Adaptive campaigns counted generation, redesign and standardized AF2 evaluation toward this budget because these computations occurred during campaign execution and could inform subsequent allocation. For generator-only baselines, generation and search counted toward the budget, whereas standardized AF2 evaluation performed only after the generation cutoff was excluded because it could not affect which designs were generated. For T-REX, LLM serving and post-hoc analyses were excluded from *H*, which is defined to capture worker compute used during the design campaign. Alternative accounting and campaign-level statistics are specified in Supplementary Sections D.1.3 and D.1.1.

### 4.3. Campaign archive and evidence construction

#### Campaign archive.

To make the evidence–planning–execution loop traceable, each campaign stored results and controller decisions in an archive that added new records without overwriting earlier ones. Three record types captured the main scientific results and planning information: (1) a *ResultRecord* recorded an output or failure from job execution; (2) an *EvidenceSummary* summarized accumulated evidence, unfinished work and available resources at each planning update; and (3) a *HypothesisCard* represented an evidence-linked hypothesis and proposed follow-up test. A *ResultRecord* linked each output or failure to its action family and route and, when applicable, to its parent design. It also stored available measurements, worker compute, execution status and links to structure, score and log files. A job could produce multiple *ResultRecords*, and individual outputs could be recorded before the job finished. If a job produced no usable design, its *ResultRecord* could still record the failure status and worker compute, even though no design measurements were available. In addition to these three record types, the archive stored candidate jobs, Supervisor rankings and allocation proportions, queue-admission decisions, confirmed worker starts, start failures, and LLM-call audits (Supplementary Section C.3).

#### EvidenceSummary construction.

Before the Planner proposed follow-up tests and candidate jobs were selected, prespecified deterministic code constructed an *EvidenceSummary* from archived results, unfinished work and available resources. The *EvidenceSummary* provided the shared campaign context for the Planner and Supervisor; a reduced view of the summary was supplied to their prompts. As illustrated in Figure 1d, the summary organized evidence about (1) Structure Quality: qualification measurements, outcomes and margins; (2) Structure Novelty: new SUs and structural duplication; (3) Exact-Route Yield + Cost: route-level outcomes, recent new SUs per GPU-hour and compute use; (4) Waiting Evals: designs awaiting standardized AF2 evaluation; (5) Parent Lineage + Outcomes: parent designs and the outcomes of their descendants; and (6) GPU Resources + Active Jobs: queued and running jobs and available worker capacity. It also retained near-miss evidence and source-specific auxiliary measurements. A near miss had complete qualification measurements, failed qualification and satisfied the prespecified near-miss rule. Auxiliary measurements were source-specific diagnostic scores not used to determine qualification, including ipTM, ipSAE, interface energy and shape complementarity. The summary kept these measurements associated with their sources, and missing values were not treated as failures or zeros. The full *EvidenceSummary* schema, recent-evidence windows defined by *ResultRecord* count or recorded worker compute, and quality calculations are specified in Supplementary Sections C.4.1–C.4.3; the reduction applied before LLM input is described in Supplementary Section C.5.2.

#### Campaign-state assignment.

Campaign-state labels provided context for LLM reasoning and deterministic allocation rules without prescribing a particular job (Figure 1d). As part of this evidence-construction step, a deterministic classifier assigned one of seven campaign-state labels: *low evidence*, *structural duplicate collapse*, *productive*, *productive with duplication*, *near-miss-enriched*, *stalled* or *deep stall*. The classifier used evidence of productivity, structural duplication, near misses and worker compute without a new SU. When the evidence satisfied the conditions for more than one campaign state, a prespecified decision order determined which label was assigned; *low evidence* served as the default when no condition matched. The assigned label was included in the *EvidenceSummary*. State definitions and thresholds are given in Supplementary Section C.4.5.

### 4.4. Evidence-guided planning and hypothesis feedback

#### LLM agent roles.

T-REX used two LLM-based controller roles, the **Planner** and **Supervisor** (Figure 1d). Both were instantiated using the same locally served Qwen3.6–27B-FP8 model with vLLM, but used separate prompts and structured output schemas (Team, 2026; Kwon et al., 2023). We selected this open-weight model to enable local deployment with a fixed, versioned model snapshot and prespecified serving configuration. The model was served on a single H100 GPU. Across the seven primary campaigns, 7 of 3,125 LLM calls (0.22%) produced responses that failed structured-output validation; these responses were rejected before launch approval and triggered deterministic fallback paths (Supplementary Table S15). Functionally, the Planner proposed what to test next and why, whereas the Supervisor prioritized which validated candidates should receive future worker starts. Neither LLM directly launched jobs; candidate construction, validation, admission, dispatch, and execution were handled by deterministic controller logic (Section 4.5; Supplementary Section C.8.2).

#### LLM decision guidance.

Both prompts provided guidance on whether follow-up work should extend a productive route, address an identified weakness, or test an alternative route or configuration. Route productivity was measured as the recent number of new TM0.6 SUs per GPU-hour charged to the route, including required evaluation and ancestor jobs. To determine which routes to prioritize for further computation, the prompts instructed both LLM roles to use recent route productivity as the primary quantitative criterion. Two routes were considered to have comparable productivity when the recent new TM0.6 SUs per GPU-hour of the less productive route was at least 85% of that of the more productive route, although both prompts advised caution when this comparison was based on few observations or little compute. For such routes, the LLM roles were instructed to prefer the route with the higher 25th percentile of its qualified-design quality scores, using the median as the next comparison (Supplementary Sections C.5.3, C.7 and C.4.3).

#### Planning.

The Planner received the *EvidenceSummary* and campaign-state context, together with up to eight active or recent *HypothesisCards*, available action families and their roles, and permitted settings and per-job workload limits. The prompt requested one to four new *HypothesisCards*, each describing an evidence-linked hypothesis and a proposed follow-up computational test. Each new card cited supporting evidence, suggested action families and settings, and indicated the test’s relevance to Rescue, Explore, and eXploit. It also predicted how selected qualification measurements should change and, when applicable, identified other measurements that should not deteriorate beyond a specified limit. Serving settings, prompt details, and call conditions are specified in Supplementary Sections C.5 and C.5.4.

#### Check + Build and candidate validation.

Planner proposals were not directly executable. Fixed Check + Build logic converted new or retained *HypothesisCards* into concrete *ActionCandidates* and validated the requested operation, registered action family, permitted parameter and workload bounds, required input files and, where applicable, parent eligibility. Permitted deterministic repairs were applied when defined; otherwise invalid candidates were rejected. This validation enforced the fixed action space, preventing the LLM from introducing unregistered action families, exceeding prespecified parameter bounds, or altering fixed campaign definitions such as the target, qualification criteria, structural-novelty rule, or worker budget. Only build-checked candidates were eligible for Supervisor prioritization and subsequent admission. Passing Check + Build established construction-time validity but did not guarantee execution; candidates remained subject to final admission rules and live dispatch-time checks described in Section 4.5.

#### Supervisor prioritization and allocation.

The Supervisor received the *EvidenceSummary* and campaign-state context, together with hypotheses from the Planner and eligible candidate jobs. It recommended candidate jobs for execution, assigning each recommended job to **Rescue**, **Explore** or **eXploit** and ranking them across all three priorities. It also returned a normalized **R/E/X** mixture specifying the desired proportions of jobs to be executed under each priority. Detailed input and output specifications, ranking guidance and allocation rules are given in Supplementary Sections C.5.2 and C.7.

#### Hypothesis feedback.

After selecting candidate jobs, the controller used prespecified rules to evaluate results from follow-up tests proposed earlier by the Planner. Results linked to each *HypothesisCard* were compared with a previously observed reference result and classified as supporting, contradictory or neutral. Accumulated results determined whether a card was supported, refuted or no longer active after its specified lifetime. These statuses informed later Planner and Supervisor decisions but did not affect design qualification or SU credit or validate the proposed causal explanation. Detailed evaluation and status-update rules are given in Supplementary Section C.9, with a worked example in Supplementary Section D.2.2.

### 4.5. Asynchronous job allocation and execution

#### Candidate job selection.

Candidate selection operated on build-checked jobs described in Section 4.4. The Supervisor assigned each recommended candidate job to **Rescue**, **Explore** or **eXploit** and ranked them across the three priorities. The controller considered these jobs in rank order and used the **R/E/X** mixture to guide how subsequent generation and redesign starts were distributed, subject to prespecified feasibility and resource constraints. If additional jobs could be admitted to the shared worker queue, the controller selected them from the remaining eligible candidates using deterministic rules based on recent starts by action family, route productivity and estimated job cost. Selected jobs entered the shared worker queue, but only confirmed generation and redesign starts contributed to the realized **R/E/X allocation**. Detailed candidate ordering, allocation, fallback and dispatch rules are given in Supplementary Section C.7 and Table S8.

#### Worker scheduling and asynchronous execution.

T-REX used three H100 workers and a separate H100 for LLM serving; PUCT and *ϵ*-greedy used four workers. T-REX had a reporting budget of 144 worker GPU-hours, corresponding to three worker slots over 48 h (3 × 48 = 144 worker GPU-hours). When a worker became free, the controller rechecked the next job’s inputs, parent eligibility and resource availability before starting or deferring it. Completed results were incorporated into campaign evidence as soon as they became available, so subsequent planning and job selection could use them while other jobs remained in progress. Detailed rules for queue admission, worker starts, concurrency limits and failure handling are given in Supplementary Section C.8, with the controller workflow summarized in Algorithm S1.

#### AF2 evaluation admission.

Standardized AF2 evaluation used the same three worker GPUs as generation and redesign but followed a separate deterministic admission policy. Designs with usable structures were eligible for this evaluation when one or more of the three required qualification measurements had not been computed during generation (Figure 1e). Prespecified deterministic controller rules determined which eligible designs were evaluated and in what order. For each route requiring AF2 evaluation, up to four designs could initially be evaluated. After results from all evaluations within the current allowance were available, the cumulative allowance could increase successively to 8, 16, 32 and 64 evaluations and then continue doubling, but only if the route showed evidence of improvement relative to the preceding allowance boundary. Improvement was defined as an increase in route-attributed new SUs, near-miss count, the number of qualified structural clusters, the 25th percentile or median of the qualified-design quality scores, or the source-specific auxiliary diagnostic score. Qualified-design quality scores were calculated from the three normalized pass margins while retaining only the highest-scoring design per structural cluster. Exact score definitions, comparison rules, capacity limits and the spare-capacity exception are specified in Supplementary Sections C.4.3 and C.8.1.

#### Campaign termination and final endpoint reconstruction.

The evidence–planning–execution loop ran until the prespecified 48-h campaign time limit; campaigns did not terminate based on campaign state, lack of new SUs or the number of SUs already obtained. The controller checked the time limit before each pass through the loop; a pass already in progress was allowed to finish. At shutdown, each running job was allowed up to 600 s to complete. Results from completed jobs were parsed. After execution stopped, the qualification criteria were reapplied to the final archived *ResultRecord* entries for outputs with a usable structure and all three required qualification measurements, including any such outputs recovered during shutdown. Foldseek TM0.6 clustering was then recomputed for the designs that qualified. Detailed shutdown, endpoint-inclusion and clustering rules are given in Supplementary Sections C.8.3, B.3 and B.2.

## Supplementary Material

Supplement 1

## Figures and Tables

**Figure 1 |:**
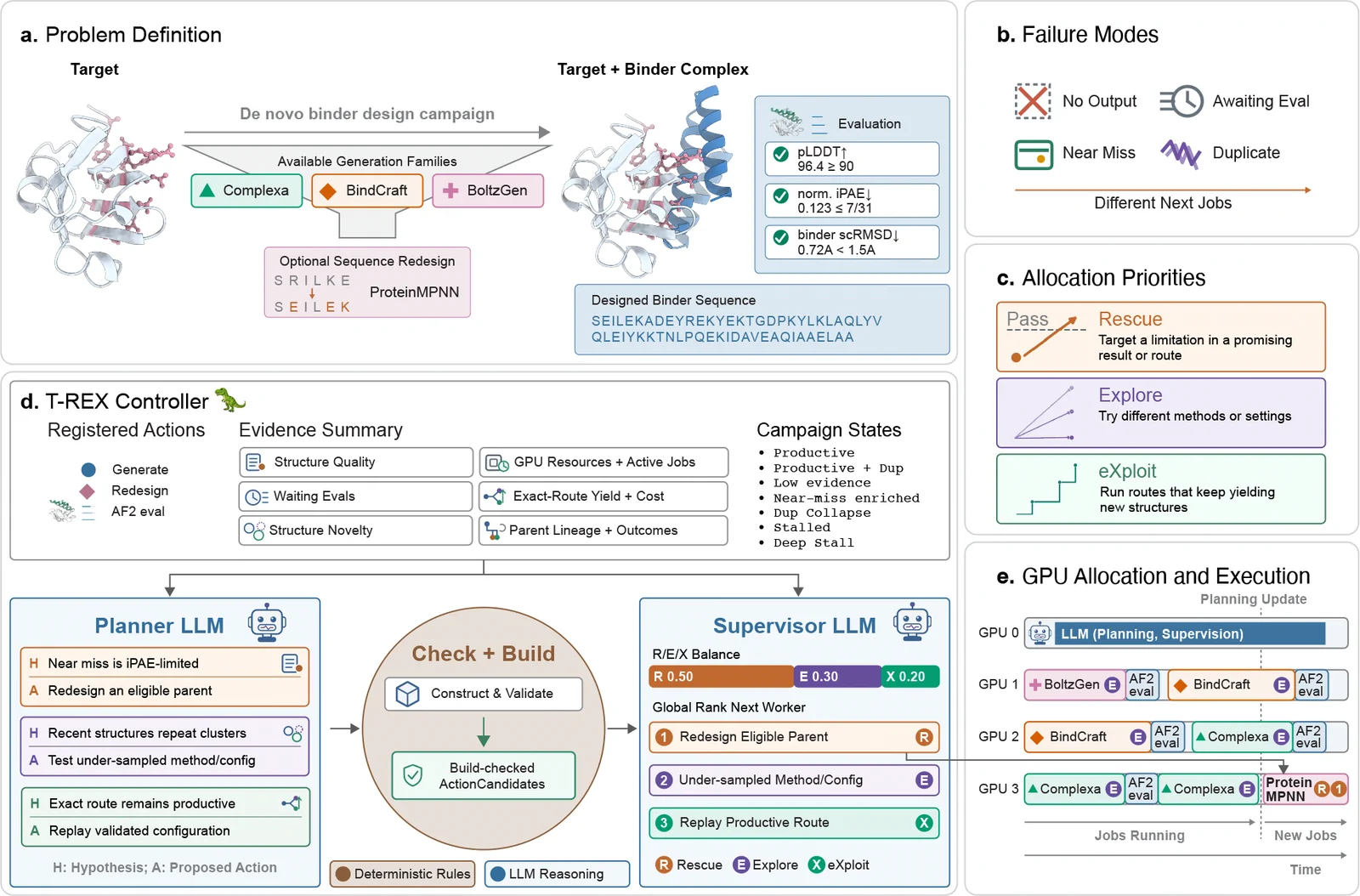
Overview of T-REX. **a.** T-REX is an agentic framework that adaptively orchestrates computational tools for *de novo* protein binder design under a fixed compute budget. **b,c.** Design outcomes are organized into failure modes that motivate distinct priorities for subsequent actions: **Rescue** promising designs, **Explore** alternative methods or settings, or **eXploit** productive routes. **d.** Evidence from completed and ongoing design jobs is organized into a structured representation of the evolving campaign state and interpreted by two LLM agents. The Planner proposes evidence-guided hypotheses and follow-up actions, while the Supervisor prioritizes validated actions and proposes their allocation across Rescue, Explore and eXploit. A deterministic controller orchestrates job execution given the available compute resources. **e.** Proposed actions are asynchronously executed across available GPUs, with completed results providing new evidence for subsequent planning and allocation.

**Figure 2 |:**
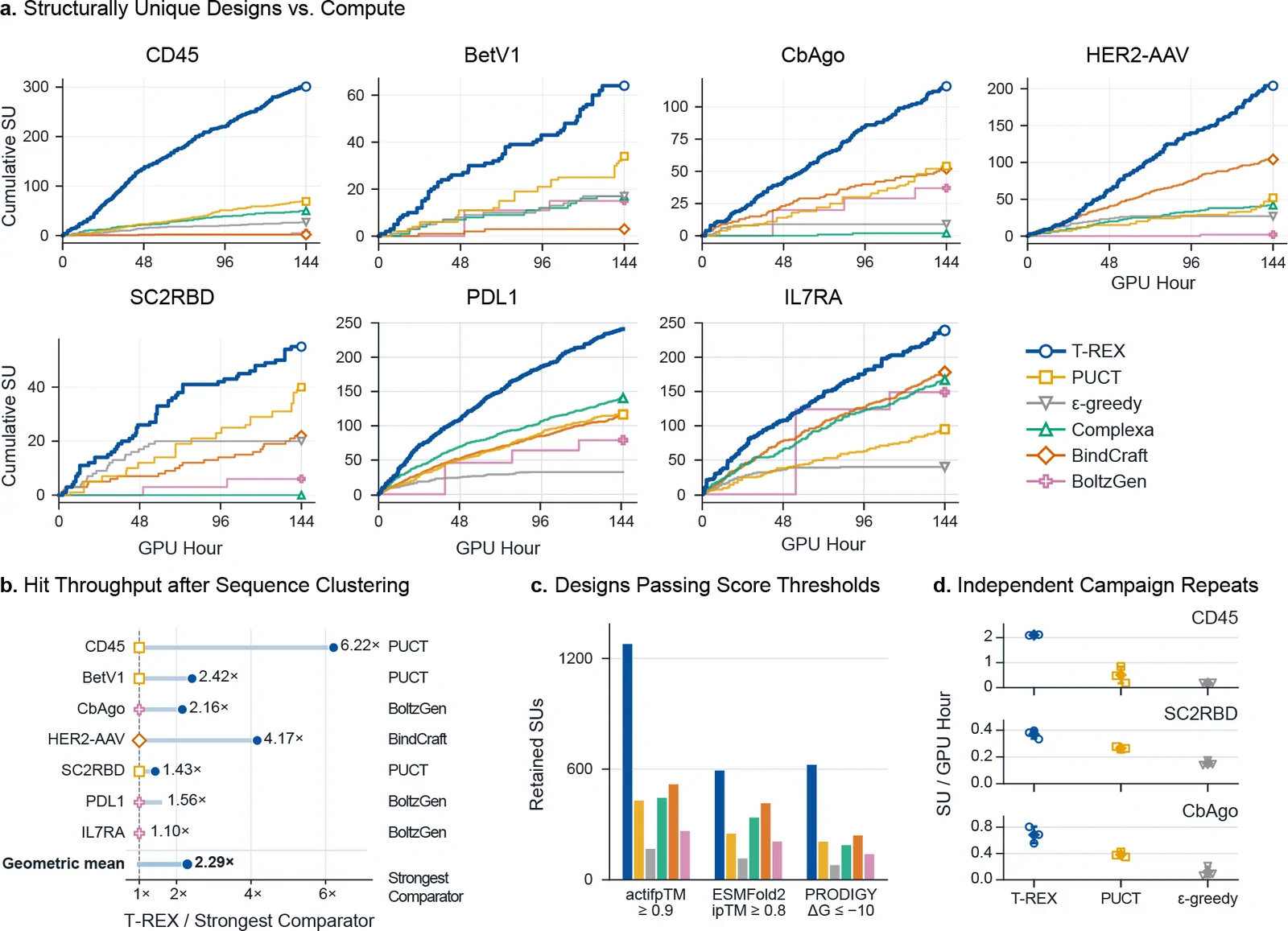
T-REX produces more structurally unique hits than baseline methods over the course of a design campaign. **a.** Cumulative number of structurally unique hits versus GPU-hours across seven targets for T-REX, non-agentic adaptive approaches and single-generator baselines. **b.** Hit throughput under MMseqs2 clustering at 70% sequence identity, shown relative to the highest-throughput alternative for each target. **c.** Number of structurally unique hits retained under alternative post-hoc quality thresholds, pooled across seven targets. actifpTM and ESMFold2 ipTM measure predicted interface confidence, and PRODIGY estimates binding free energy (Δ*G*, kcal mol^−1^). Each threshold is applied separately to the same set of TM0.6 cluster representatives. **d.** Hit throughput across three independently initialized campaigns for T-REX, PUCT and *ϵ*-greedy on CD45, SC2RBD and CbAgo. Points show individual campaigns; diamonds and error bars show the mean and s.d.

**Figure 3 |:**
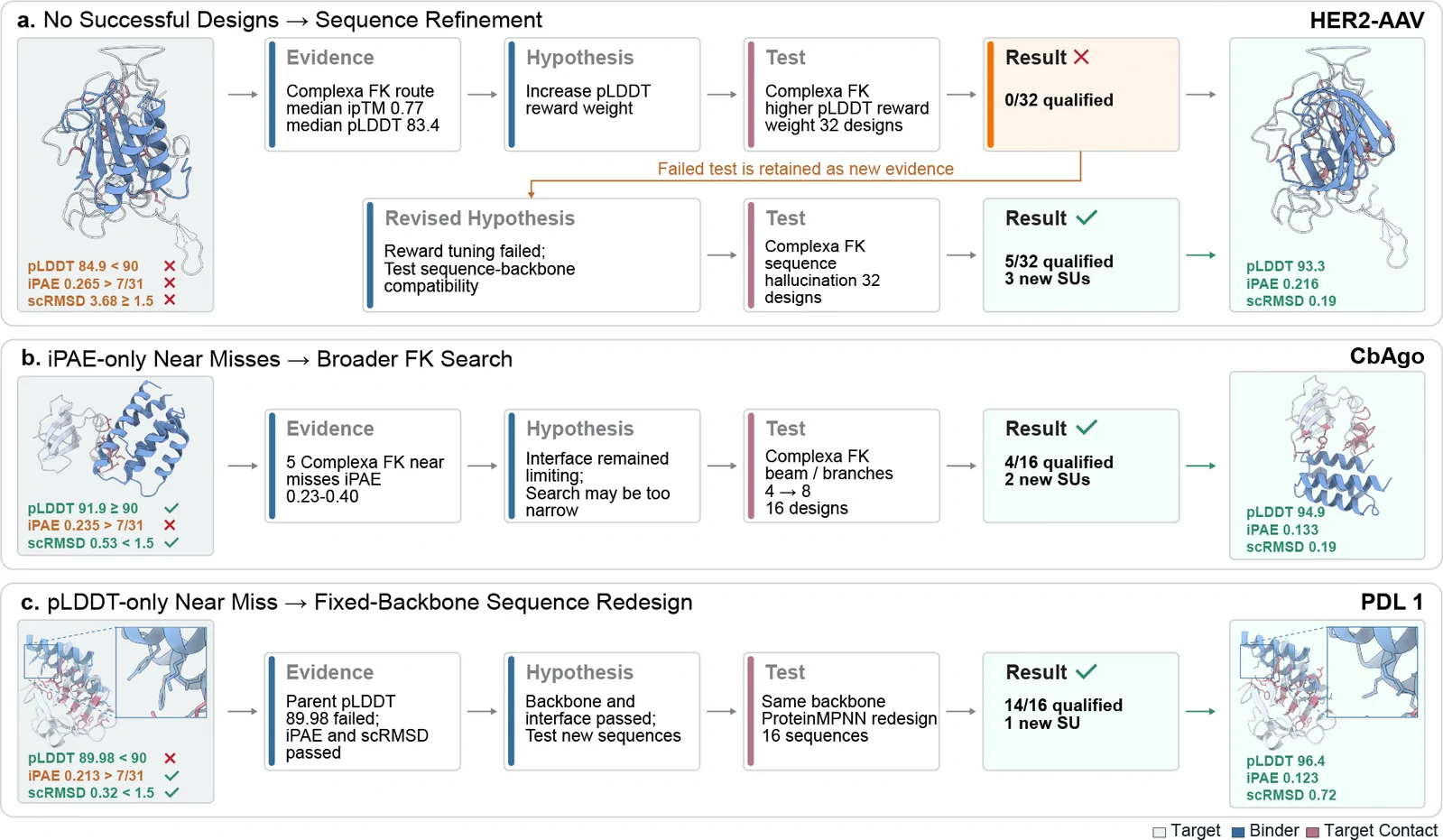
T-REX uses campaign outcomes to propose follow-up design strategies. Three retrospective examples illustrate how T-REX uses observed outcomes to propose subsequent computational actions. **a.** For HER2-AAV, a batch optimizing pLDDT produced no successful designs, motivating sequence refinement that produced 5/32 successful designs and three new structurally unique hits. **b.** For CbAgo, repeated designs failing only the iPAE criterion motivated broader Feynman–Kac (FK) search, producing 4/16 successful designs and two new structurally unique hits. **c.** For PDL1, a design failing only the pLDDT criterion motivated fixed-backbone ProteinMPNN sequence redesign, producing 14/16 successful designs and one new structurally unique hit. Orange and green labels indicate failed and passed design criteria, respectively.

**Figure 4 |:**
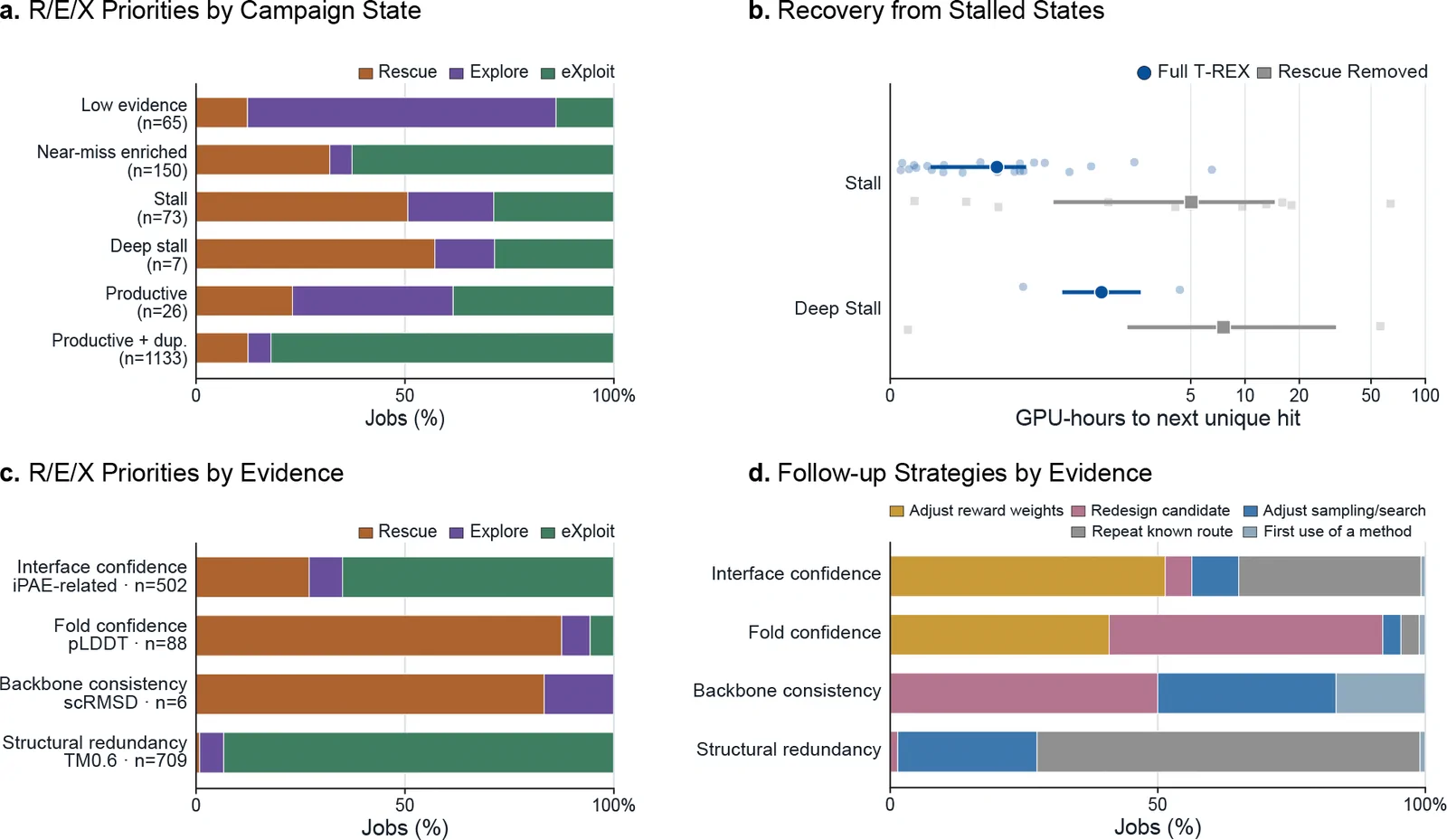
T-REX adapts design strategies to campaign state and accumulated evidence. **a.** Rescue–Explore–eXploit (R/E/X) priorities across 1,454 generation and redesign jobs, grouped by campaign state. **b.** GPU-hours from entry into a stalled campaign state to the next structurally unique hit for T-REX and T-REX without an explicit Rescue priority. Points show individual recovery events; larger markers and bars show medians and interquartile ranges. **c.** R/E/X priorities across 1,305 generation and redesign jobs, grouped by the primary evidence informing each decision. **d.** Follow-up design strategies for the same jobs, grouped by primary evidence as in **c**.

**Figure 5 |:**
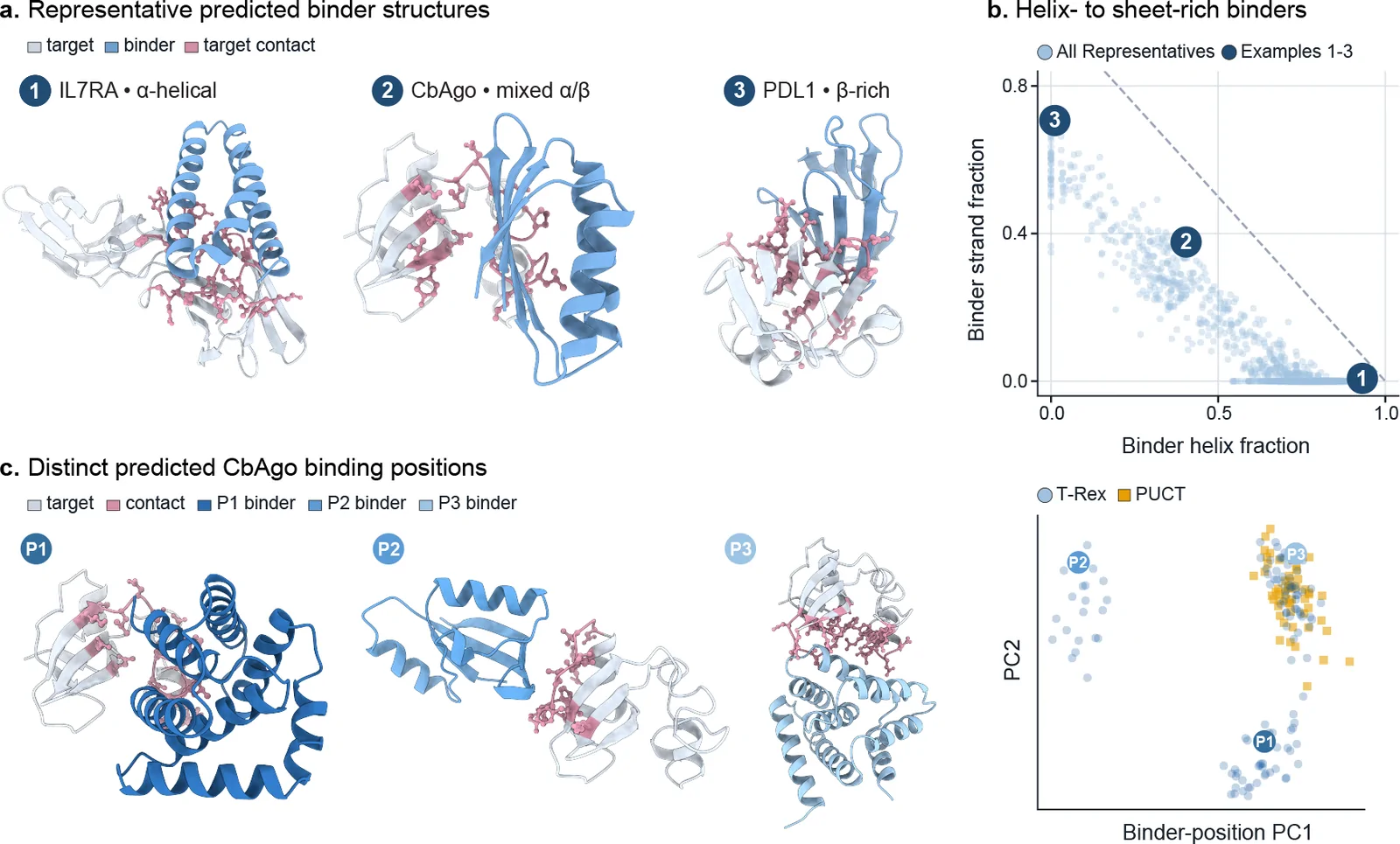
T-REX produces binders with diverse structures and predicted binding positions. **a.** Representative predicted complexes showing an *α*-helix-rich IL7RA binder, a mixed *α*-helix/*β*-strand CbAgo binder and a *β*-rich PDL1 binder. **b.** Secondary-structure fractions of T-REX hits (*n* = 1, 461), calculated using DSSP. Numbered points correspond to the examples in **a**. **c.** Representative T-REX hits at distinct predicted binding positions on CbAgo. **d.** Principal component analysis (PCA) of target-relative binder positions for hits from T-REX (*n* = 116) and PUCT (*n* = 54).

**Table 1 |:** Protein binder design hit throughput for T-REX and baseline methods.

Target	T-REX	PUCT	*ϵ*-gr.	CX	BC	BG	T-REX gain	
							vs PUCT	vs best GO

CD45	**2.090**	0.479	0.188	0.347	0.014	0.035	4.36×	6.02×
BetV1	**0.444**	0.236	0.118	0.118	0.021	0.104	1.88×	3.76×
CbAgo	**0.806**	0.375	0.062	0.014	0.361	0.257	2.15×	2.23×
HER2-AAV	**1.417**	0.361	0.188	0.292	0.722	0.014	3.92×	1.96×
SC2RBD	**0.382**	0.278	0.139	0.000	0.153	0.042	1.38×	2.50×
PDL1	**3.347**	1.618	0.451	1.951	1.604	1.097	2.07×	1.72×
IL7RA	**1.660**	0.660	0.278	1.160	1.236	1.035	2.52×	1.34×

Hit throughput is reported as the number of structurally unique hits per GPU-hour under a total 144 H100 worker GPU-hour budget. Hits are designs that pass the prespecified *in silico* criteria and are clustered with Foldseek at a TM-score threshold of 0.6. Gain columns report the T-REX ratio relative to PUCT and the highest-throughput generator-only (GO) baseline for each target. *ϵ*-gr., *ϵ*-greedy; CX, Complexa-only; BC, BindCraft-only; BG, BoltzGen-only. Bold and underline indicate the highest and second-highest throughput, respectively, for each target.

## Data Availability

Design target inputs are listed in Supplementary Table S1. HER2-AAV, SC2RBD, PDL1 and IL7RA derive from Protein Data Bank (PDB) entries 1N8Z, 6M0J, 5O45 and 3DI3, respectively, with cropping or repacking recorded in Supplementary Table S1 (Cho et al., 2003; Lan et al., 2020; Magiera-Mularz et al., 2017; McElroy et al., 2009).

## Code availability

The T-REX source code is available at https://github.com/ml-struct-bio/T-REX. The repository includes installation instructions, example configurations and tools for running campaigns and analyzing campaign archives.
