# Supplementary material for: Agentic campaign control for high-throughput de novo binder design
Source: bioRxiv. 2026 Sep 24:2026.09.22.753604. Preprint. [Version 2] doi: 10.64898/2026.09.22.753604 (PMC13622205; doi:10.64898/2026.09.22.753604)
Supplement: Supplementary file 1 [file NIHPP2026.09.22.753604v2-supplement-1.pdf]

bioRxiv preprint doi: https://doi.org/10.64898/2026.09.22.753604; this version posted September 24, 2026. The copyright holder for this preprint (which was not certified by peer review) is the author/funder, who has granted bioRxiv a license to display the preprint in perpetuity. It is made available under a CC-BY 4.0 International license.

Supplementary Information

A Data and target specifications . . . 23
A.1 Primary benchmark inputs . . . 23
B Evaluation metrics and benchmark design . . . 23
B.1 Qualification criteria and evaluation . . . 23
B.2 Sequence and structural clustering . . . 24
B.3 Compute budget and endpoint inclusion . . . 25
B.4 Baseline setup and adaptive initialization . . . 26
B.5 Adaptive comparator policies . . . 26
B.6 Related work: protein-design workflows and campaign control . . . 27
B.7 Campaign replication . . . 28
C Campaign controller implementation . . . 29
C.1 Controller workflow and agent roles . . . 29
C.2 Software and permitted job configurations . . . 32
C.3 Campaign archive and runtime state . . . 34
C.4 EvidenceSummary construction and derived measures . . . 37
C.5 Language-model inputs, outputs and call handling . . . 44
C.6 Candidate construction and validation . . . 50
C.7 Supervisor prioritization and deterministic selection . . . 54
C.8 Evaluation admission and worker execution . . . 58
C.9 Hypothesis feedback . . . 62
D Supplementary experiments and campaign analyses . . . 63
D.1 Throughput and robustness . . . 64
D.2 Evidence-linked decisions and worked examples . . . 70
D.3 Controller behavior and intervention . . . 76
D.4 Post-hoc characterization of binder designs . . . 81

Reading guide. Section A specifies target inputs. Section B defines evaluation and benchmark comparisons. Section C describes the controller from stored observations through evidence, proposals, selection, execution and feedback. Section D groups supporting analyses for main Figures 2–5, including archived decisions and post-hoc characterization analyses. Monospace text denotes schema fields, settings or explicitly labeled example identifiers.

bioRxiv preprint doi: https://doi.org/10.64898/2026.09.22.753604; this version posted September 24, 2026. The copyright holder for this preprint (which was not certified by peer review) is the author/funder, who has granted bioRxiv a license to display the preprint in perpetuity. It is made available under a CC-BY 4.0 International license.

A. Data and target specifications

A.1. Primary benchmark inputs

Unless otherwise stated, qualification, outcome and post-hoc diagnostic tables use the fixed seven-target set: CD45, BetV1, CbAgo, HER2-AAV, SC2RBD, PDL1 and IL7RA. These targets were selected from the benchmark assembled by Proteina-Complexa, and we retained its target structures, chain and residue selections, hotspot definitions and default binder-length ranges (Didi et al., 2026b). Table S1 lists the exact frozen specifications used here.

Table S1 | **Fixed target inputs and default binder-length ranges.** The table shows the frozen target specifications used in the primary benchmark. Entries without a PDB accession are identified as AF2 predictions. Residue and hotspot numbering follows each input structure. The binder-length column gives the target-specific default range; adaptive-controller BindCraft actions could vary these design bounds within the prespecified range without changing the target definition. Source denotes the benchmark from which the specification originated.

Target	PDB ID	Target residues	Hotspot residues	Binder length	Source
CD45	AF2 prediction	A1–88	A1	80–200	BindCraft
BetV1	AF2 prediction	A1–159	A24	70–185	BindCraft
CbAgo (PAZ)	AF2 prediction	A180–276	A268	70–160	BindCraft
HER2-AAV	1N8Z	C23–101, C111–268	C165, C170	60–100	BindCraft
SC2RBD	6M0J	E333–526	E485, E489, E494, E500, E505	80–120	AlphaProteo
PDL1	5O45	A17–132	A56, A115, A123	50–120	AlphaProteo
IL7RA	3DI3	B17–209	B58, B80, B139	50–120	AlphaProteo

B. Evaluation metrics and benchmark design

B.1. Qualification criteria and evaluation

Qualification required binder pLDDT ≥ 90, normalized iPAE $\leq 7/31$ and binder scRMSD < 1.5 Å under the common AF2 protocol, with thresholds adapted from Proteina-Complexa (Didi et al., 2026b). pLDDT is the mean confidence over binder residues in the predicted complex, reported on a 0–100 scale (90 equals 0.90 on the 0–1 scale). Normalized iPAE averages the two directional binder–target PAE means and divides by 31 Å; the dimensionless cutoff $7/31$ corresponds to an unnormalized mean of 7 Å. Binder scRMSD compares generated and AF2-predicted binder Cα coordinates after Kabsch alignment of the corresponding target Cα coordinates.

AlphaFold2 evaluation (Jumper et al., 2021; Evans et al., 2021) used `model_1_multimer_v3`, three recycles, no dropout, generated-complex initial coordinates and a binder template with inter-chain template contacts removed. Adaptive evaluation derived its seed from the first eight hexadecimal digits of SHA-256 over `candidate_id|af2_refilter|s{RUN_SEED}`, interpreted as an integer modulo 10^6; generator-only evaluation used rank-index seeds. Complexa supplied the common AF2 measurements during generation when available, whereas outputs without a complete set required separate standardized AF2 evaluation. Harmonized AF2 was a separate post-hoc analysis and did not affect allocation, qualification or SU credit.

bioRxiv preprint doi: https://doi.org/10.64898/2026.09.22.753604; this version posted September 24, 2026. The copyright holder for this preprint (which was not certified by peer review) is the author/funder, who has granted bioRxiv a license to display the preprint in perpetuity. It is made available under a CC-BY 4.0 International license.

B.1.1. Outputs admitted to evaluation

An individual design was eligible for standardized AF2 evaluation when its output was recorded as successful and its structure was available in Protein Data Bank (PDB), Crystallographic Information File (CIF) or macromolecular CIF (mmCIF) format. Evaluation could begin while the producing job was still running (Section C.8.2). The structure could be supplied directly or retrieved from its output directory.

Complexa and BindCraft outputs. A final sample from Complexa (Didi et al., 2026b) was admitted when a valid structure was available. Inline measurements from the common qualification protocol were used when present; otherwise the structure entered standardized AF2 evaluation. Final PDBs from BindCraft (Pacesa et al., 2025) were eligible for standardized AF2 evaluation regardless of whether they passed BindCraft's own filters.

BoltzGen outputs. For BoltzGen (Stark et al., 2025), the fixed reference admitted individual designs listed in BoltzGen's metrics table and excluded aggregate root structures. CIF files were converted to PDB, and up to 10,000 designs per target were admitted in ascending order of BoltzGen's final rank before qualification outcomes were inspected. Adaptive BoltzGen admitted completed individual designs with resolvable per-design structures, again excluding aggregate root structures. BoltzGen's own measurements could inform allocation, but qualification required all three measurements from the common AF2 protocol. Only a subset of adaptive BoltzGen designs underwent this evaluation during the campaign.

ProteinMPNN outputs. For ProteinMPNN (Dauparas et al., 2022), a redesigned child was admitted when its generated sequence, from a FASTA-formatted row beginning with `sample=`, had been successfully threaded. The child entered standardized AF2 evaluation as a new design; evaluating its parent did not establish the child's qualification.

B.2. Sequence and structural clustering

Foldseek clustered qualified binder chains, not full complexes. Sorted inputs were processed with `easy-cluster`, minimum sequence identity 0, minimum coverage 0, coverage mode 0, alignment type 1, alignment-length TM-score normalization (`--tmscore-threshold-mode 0`) and one thread. TM0.5 and TM0.8 changed only the primary TM0.6 threshold.

Representative-based set-cover clustering defines similarity to cluster representatives, not an all-pairs criterion or biological fold classes; higher thresholds generally give finer partitions (Van Kempen et al., 2024; Xu and Zhang, 2010; Jendrusch and Korbel, 2025; Liu et al., 2026). Sensitivity analyses re-clustered the same qualified pools without rerunning controllers. Native generator clusters did not determine cross-method SU credit.

MMseqs2 clustered qualified binder sequences before structural deduplication at 70% identity, 0.8 coverage and coverage mode 0 (Steinegger and Söding, 2017). Tables S13 and S14 report sequence throughput and structural-threshold sensitivity, respectively.

bioRxiv preprint doi: https://doi.org/10.64898/2026.09.22.753604; this version posted September 24, 2026. The copyright holder for this preprint (which was not certified by peer review) is the author/funder, who has granted bioRxiv a license to display the preprint in perpetuity. It is made available under a CC-BY 4.0 International license.

Fixed representatives. For every qualified design, normalized qualification margins were

$$\begin{aligned} m_{\mathrm{pLDDT}} &= (\mathrm{pLDDT} - 90)/10, \\ m_{\mathrm{iPAE}} &= (7/31 - \mathrm{iPAE})/(7/31), \\ m_{\mathrm{scRMSD}} &= (1.5 - \mathrm{scRMSD})/1.5, \\ m_{\min} &= \min\{m_{\mathrm{pLDDT}}, m_{\mathrm{iPAE}}, m_{\mathrm{scRMSD}}\}. \end{aligned}$$

Each Foldseek TM0.6 cluster contributed the representative with the largest $m_{\min}$; ties were broken, in order, by larger mean margin, larger pLDDT margin, larger iPAE margin, larger scRMSD margin and finally a stable record identifier (lexicographically smaller for Complexa-only and larger for the other methods). These fixed representatives were used for final generation/redesign attribution, post-hoc analyses and qualification-margin sensitivity. During campaigns, the controller used separate route-specific representatives and quality summaries, defined in Section C.4.3.

B.3. Compute budget and endpoint inclusion

The primary benchmark used a fixed reporting denominator of 144 H100 worker GPU-h per method–target pair. T-REX used three worker GPUs plus one GPU for LLM serving; PUCT and ϵ-greedy used four workers. LLM serving and post-hoc diagnostic predictions were excluded from this denominator. Adaptive campaigns charged generation, redesign and standardized AF2 evaluation to the worker budget. For generator-only baselines, separate AF2 evaluation was performed post hoc after the 144 worker GPU-h generation budget. The comparison therefore matched worker compute rather than wall-clock duration. Final adaptive counts included eligible outputs collected during shutdown, without adding shutdown time to the reporting denominator. Generator-only counts used the admitted generation cohort defined in Section B.1.1. Table S2 summarizes execution differences. Compute-accounting sensitivities are reported in Section D.1.3; runtime and shutdown procedures are specified in Section C.8.3.

Table S2 | **Shared and condition-specific fields in the primary benchmark.** This table separates the common scientific task and endpoint from the allocation policy and its execution protocol.

Field	Shared across compared conditions	Condition-specific part
Target	Frozen target coordinates, chains and hotspot residues.	None within a target comparison.
Allowed work	The adaptive controllers used the same eight registered action families, role definitions, target-specific permitted binder-length ranges, parameter bounds, workload limits and parent-validity rules.	Each generator-only condition used only its named generator; its fixed settings are reported in Supplementary Section B.4.
Candidate proposal and ranking	Adaptive policies draw from the shared action-space bounds; the available candidates depend on campaign evidence and usable parents.	T-REX combines LLM proposals with deterministic construction, repair and additional candidates. PUCT and ϵ-greedy use progressive configuration sampling and scalar ranking (Section B.5).
Measurement	Common qualification rule, AF2 measurements eligible for qualification, Foldseek clustering and reported-metric definitions.	None for the primary endpoint; alternative clustering analyses are labeled separately.
Control and resources	Common artifact-validity checks and the 144 H100 worker GPU-hour reporting budget.	Allocation policy, queue and worker protocol, launch and completion windows and endpoint eligibility were condition specific. Generator-only AF2 evaluation occurred after its generation cutoff and could not affect generation; the corresponding sensitivity is reported in Supplementary Table S12.

bioRxiv preprint doi: https://doi.org/10.64898/2026.09.22.753604; this version posted September 24, 2026. The copyright holder for this preprint (which was not certified by peer review) is the author/funder, who has granted bioRxiv a license to display the preprint in perpetuity. It is made available under a CC-BY 4.0 International license.

B.4. Baseline setup and adaptive initialization

Each generator-only baseline repeatedly ran one prespecified workflow under the common 144 worker GPU-h generation budget: Proteina-Complexa's Beam workflow, BindCraft's default four-stage multimer workflow or BoltzGen's published `protein-anything` recipe. Configurations were fixed across targets apart from the target inputs and binder-length intervals, and none was selected using T-REX outcomes. The adaptive controllers used the same generator implementations and learned checkpoints. Table S4 lists the versions, baseline settings and initial adaptive workloads; Table S1 specifies target inputs.

Adaptive campaigns began with prespecified Complexa Beam, BoltzGen and BindCraft jobs to obtain initial evidence. The initial Complexa settings matched Complexa-only; BindCraft and BoltzGen used smaller initial workloads to obtain earlier feedback, as specified in Table S4. Subsequent jobs varied within the bounds in Table S5. Successive Complexa runs used non-overlapping explicit seeds, BindCraft trajectories drew fresh stochastic seeds, and fixed BoltzGen runs were initialized from system entropy. Campaign replication is described in Section B.7.

Development scope. Controller settings were fixed rather than fitted online. Development runs used to calibrate the threshold for worker time without a new SU included some primary-panel targets. Here, target-independent means that the same runtime rules apply without target-identity branches, not that development used a disjoint held-out target set. The campaign-state definitions are given in Section C.4.5.

B.5. Adaptive comparator policies

The primary adaptive comparison shares the action-space bounds, scientific endpoint and required-evaluation machinery, while candidate proposal and ranking differ. T-REX combines LLM proposals with deterministic candidate construction, repair and supplementation (Section C.6); PUCT and ϵ-greedy use progressive configuration sampling and scalar ranking. Their shared AF2-admission policy can use diagnostic evidence (Section C.8.1) even though that evidence does not enter scalar ranking. These comparisons evaluate the complete allocation policies and do not isolate the contribution of LLM reasoning from the other policy differences.

B.5.1. Adaptive scalar-reward comparators

PUCT. Our PUCT comparator searched the same action space as T-REX without an LLM, using a prior-weighted exploration score adapted from tree-search allocation (Rosin, 2011; Silver et al., 2017; Aygün et al., 2026). It selected an action family and then a configuration or eligible parent-bound action within that family. At decision step k, the scalar reward was online new TM0.6 SU per charged worker GPU-h:

$$Q_k(a) = \frac{N_{\text{new SU},k}(a)}{H_k(a)}. \tag{S1}$$

Here, $N_{\text{new SU},k}(a)$ and $H_k(a)$ are the cumulative new-SU count credited to action a and its charged worker GPU-hours, with $Q_k(a) = 0$ before any cost is recorded. Family rewards use the corresponding totals across their actions. Candidate configurations were progressively sampled within the shared parameter bounds.

At each level, the highest-scoring available child a of parent p was selected using

$$\text{score}_k(a) = \text{RankScore}(Q_k(a)) + c_{\text{puct}} P(a) \frac{\sqrt{\max(1, N_p)}}{1 + N_a}, \qquad P(a) = \frac{1}{|\mathcal{C}(p)|}. \tag{S2}$$

bioRxiv preprint doi: https://doi.org/10.64898/2026.09.22.753604; this version posted September 24, 2026. The copyright holder for this preprint (which was not certified by peer review) is the author/funder, who has granted bioRxiv a license to display the preprint in perpetuity. It is made available under a CC-BY 4.0 International license.

Here, $\mathcal{C}(p)$ is the available child set, $c_{\text{puct}} = 1.0$, and N_p, N_a are visit counts including queued, running and newly assigned work as virtual visits. For m distinct sibling rewards, the jth value in ascending order has $\text{RankScore} = (j-1)/(m-1)$; it is zero when $m = 1$. This normalization puts the reward term on a 0–1 scale, while the second term favors less-visited actions.

ϵ-greedy. The ϵ-greedy comparator used the same action space, configuration-sampling procedure and scalar reward $Q_k(a)$ in Eq. (S1), without an LLM (Vermorel and Mohri, 2005). It first prioritized unvisited feasible actions. Once none remained, it selected uniformly among available actions with probability $\epsilon = 0.10$ and otherwise selected the action with the highest $Q_k(a)$. Both comparators used the shared validation, required AF2 evaluation, worker execution and qualification procedures.

B.5.2. Bayesian-optimization comparator

As an additional no-LLM comparator, SMAC3 searched the same action families and parameter bounds using a random-forest surrogate and expected-improvement acquisition (Lindauer et al., 2022). The objective was negative cumulative online new TM0.6 SU per route worker GPU-h. Initial sampling prioritized unobserved families and configurations; model fitting began after at least eight completed configuration observations with two distinct objective values.

The surrogate used action family and configuration, without scientific diagnostic fields or parent identity. Its acquisition score ranked feasible candidate jobs under the shared execution and qualification procedures. One campaign per target used four H100 workers for 25 h, with a reporting denominator of 100 worker GPU-h. Software versions and surrogate settings are listed in Table S4. The comparison with retrospective 100-worker-GPU-h prefixes is reported in Section D.1.2 and Table S11.

B.6. Related work: protein-design workflows and campaign control

Protein-design workflows and adaptive execution. ProteinDJ provides modular, parallel protein-design workflows and parameter sweeps (Silke et al., 2026); BinderFlow combines parallel binder-design batches with monitoring, hit-count-based stopping and candidate refinement (González-Rodríguez et al., 2025). Locuaz uses binding-affinity scores from MD-sampled complexes to retain or prune mutation lineages (Barletta et al., 2024), and BioPipelines demonstrates iterative redesign of small-molecule binding pockets using Boltz-2 predictions (Quargnali and Rivera-Fuentes, 2026). IMPRESS couples result-dependent ProteinMPNN–AlphaFold refinement with asynchronous execution and dynamic resource management (Alsaadi et al., 2025); Colmena provides more general infrastructure for result-guided scientific computation (Ward et al., 2025). These studies establish relevant precedents for workflow integration, feedback-directed optimization and adaptive execution. Our question concerns how to choose subsequent generation and redesign jobs across complementary methods as evidence accumulates within a finite-compute campaign.

Scientific agents and binder-design campaigns. ProtAgents combines LLM planning and critique with protein generation and physics-based analysis (Ghafarollahi and Buehler, 2024). The Virtual Lab develops a workflow for mutating existing nanobodies, with the resulting designs experimentally tested by human researchers (Swanson et al., 2025). ProteinMCP demonstrates autonomous protein-engineering workflows, including binder and nanobody design (Xu et al., 2026), while PDAgent uses template retrieval and conservation-aware directed mutation (Ouyang et al., 2026). The AutoBinder Agent preprint describes binder-workflow orchestration through the Model Context Protocol (MCP) and reports prompt-based evaluations of tool selection and execution order (Ge et al., 2026). The

bioRxiv preprint doi: https://doi.org/10.64898/2026.09.22.753604; this version posted September 24, 2026. The copyright holder for this preprint (which was not certified by peer review) is the author/funder, who has granted bioRxiv a license to display the preprint in perpetuity. It is made available under a CC-BY 4.0 International license.

Claude binder-design technical report is a close precedent: agents choose methods and settings, manage GPU work within resource limits and deliver ranked designs for experimental testing (Claude Science and Shanehsazzadeh, 2026). It establishes that budget-aware multi-tool binder campaigns already form part of agentic protein design.

Campaign control and evaluation in T-REX. T-REX combines criterion-specific qualification outcomes, structural redundancy, computational cost and pending evaluations to guide evidence-linked follow-up tests and **Rescue**, **Explore** and **eXploit** priorities. LLM agents propose and prioritize work; a deterministic execution layer constructs and validates candidate jobs, enforces resource constraints and manages asynchronous execution while ongoing computations continue (Section C). We evaluate this controller by TM0.6 SU per worker GPU-h under common in silico qualification criteria across seven fixed targets, comparing it with generator-only workflows and implemented no-LLM allocation policies (Sections B and B.5). The related systems discussed above were not rerun here, so this evaluation establishes no performance advantage over them. The comparisons assess the complete controller and do not isolate the contribution of LLM reasoning.

B.7. Campaign replication

The campaign was the unit of replication. The primary seven-target benchmark included one campaign per method–target pair. For T-REX, PUCT and ϵ-greedy, two additional independently initialized campaigns on CD45, SC2RBD and CbAgo gave $n = 3$ per controller–target pair, using the same setup and prespecified denominator of 144 H100 worker GPU-h. In Table S9, Run 0, Run 1 and Run 2 correspond to controller seeds 0, 1 and 2, respectively.

bioRxiv preprint doi: https://doi.org/10.64898/2026.09.22.753604; this version posted September 24, 2026. The copyright holder for this preprint (which was not certified by peer review) is the author/funder, who has granted bioRxiv a license to display the preprint in perpetuity. It is made available under a CC-BY 4.0 International license.

C. Campaign controller implementation

C.1. Controller workflow and agent roles

T-REX combines Planner and Supervisor LLMs with deterministic candidate construction, selection and execution. Table S3 distinguishes their inputs and responsibilities; Algorithm S1 specifies the event order. Newly recorded results inform later planning updates (Figure S2b), while qualification and SU credit follow fixed evaluation rules.

Table S3 | Controller responsibilities, inputs and outputs. Brown and blue distinguish deterministic rules and LLM reasoning (Figure 1d). Rows group responsibilities rather than execution order. Current *HypothesisCards* can include new or retained Planner proposals. Worked examples appear in Sections D.2.1–D.2.2.

Component	Handled by	Inputs	Output or decision	Details (section)
**Evidence and campaign state**				
Campaign summary and state	Deterministic rules	Recorded results, queued/running jobs and available resources.	An *EvidenceSummary* that includes one of seven campaign states.	C.3–C.4
**Hypotheses, candidate jobs and prioritization**				
Planner	LLM	Reduced *EvidenceSummary*; active or recent *HypothesisCards* from the campaign archive; available action families and permitted settings.	*HypothesisCards* with evidence-linked hypotheses, follow-up tests and predicted measurement changes.	C.5.2–C.5.3
Check + Build	Deterministic rules	Current *HypothesisCards* from Planner; *EvidenceSummary*; action space; parent designs.	Constructs and validates candidate jobs within the action space, including candidates added by fixed rules.	C.6
Supervisor	LLM	Reduced *EvidenceSummary*; current *HypothesisCards* from Planner; valid candidate jobs from Check + Build; worker and queue information.	Proposed R/E/X shares of future starts and an optional ranking of a subset of valid candidate jobs.	C.5.2, C.7
LLM response checks	Deterministic rules	Planner and Supervisor responses, each checked before use.	Validated responses, permitted repairs or a fallback if the response is unusable.	C.5.2, C.7.1
**Job selection, scheduling and starts**				
Candidate-job selection	Deterministic rules	Valid candidate jobs; Supervisor output; allocation history; resources.	Selects jobs using permitted Supervisor rankings or deterministic allocation rules.	C.7
Required AF2 evaluation	Deterministic rules	Unevaluated designs; available workers.	Admits standardized AF2 jobs under fixed limits to the shared queue.	C.8.1
Job scheduling and starts	Deterministic rules	Selected jobs, required inputs and available workers.	Rechecks inputs and resource constraints; records confirmed starts, temporary deferrals or start failures.	C.8.2
**Job execution, campaign archive and feedback**				
Job execution	Scientific tools	Started jobs, with required inputs and settings.	Binder designs and computational measurements, or unsuccessful outcomes.	C.2, C.8.3
Result collection	Deterministic rules	Tool outputs, job settings and execution information.	Archives results linked to jobs and design lineage for subsequent summaries.	C.3, C.8.2
Hypothesis feedback	Deterministic rules	Earlier active *HypothesisCards*; linked results; comparison baselines.	Tests predicted measurement changes and updates card status for later planning.	C.9

bioRxiv preprint doi: https://doi.org/10.64898/2026.09.22.753604; this version posted September 24, 2026. The copyright holder for this preprint (which was not certified by peer review) is the author/funder, who has granted bioRxiv a license to display the preprint in perpetuity. It is made available under a CC-BY 4.0 International license.

Algorithm notation. Algorithm S1 uses the campaign archive $\mathcal{D}$ (Section C.3), an ordered queue $\mathcal{Q}$ of admitted job identifiers awaiting a start, and an ordered pool $\mathcal{W}$ of W worker slots. Define

$$K(\mathcal{Q}) = \max\{0, W - |\mathcal{Q}|\}, \qquad F(\mathcal{W}) = W - |\mathcal{W}_{\text{busy}}|,$$

where $\mathcal{W}_{\text{busy}}$ contains the occupied worker slots. K counts positions needed to reach the target queue depth W; F counts free workers. AF2 reservations can temporarily exceed the queue target. The campaign wall-time limit $T_{\max}$ is checked at each loop entry (Section C.8.3). The reporting denominator H is defined in Section B.3; the final qualified-cluster count N_{SU} follows the clustering rules in Section B.2.

The current EvidenceSummary is E, the campaign-state label is s, available HypothesisCards are $\mathcal{H}$, valid candidate jobs for Supervisor prioritization are $\mathcal{J}$, and selection decisions are $\mathcal{L}$. $\mathcal{J}$ includes generation, redesign and permitted diagnostic AF2 refolds; required standardized AF2 evaluations use the separate reservation procedure (Section C.2.2). The Planner also returns any response warning or fallback reason f_P; S is the Supervisor response. The resolved selection policy $\mathcal{P}$ contains permitted candidate rankings, including any usable global order of the subset returned by the current Supervisor response (the *fresh global order*), adjusted **Rescue–Explore–eXploit** proportions, and each candidate's priority and resource information. Planner fallback reasons and Supervisor validation or abstention determine which response fields can be used (Section C.7.1). The carried allocation difference $\mathbf{d}$ tracks allocation targets relative to confirmed starts and is reconstructed from archived decisions before LLM input preparation (Section C.7.3). It is included in the Supervisor's selection context.

Procedure conventions. Procedures update the archive, queue and worker states in place. Planner and Supervisor procedures include input preparation, response validation and call records. Invalid, empty or abstaining Planner output supplies no new cards; retained valid cards can still be used. The Supervisor LLM is called only when cards and valid candidate jobs are available (Section C.5.4).

Before the first dispatch, a state check obtains s from recorded results and worker and queue information, or reuses its cached result. It appends no EvidenceSummary and calls neither LLM. DispatchReady rechecks queued jobs and records starts, deferrals or failures, with bounded retries (Section C.8.2). Running jobs continue during synchronous LLM calls; new starts are attempted at the two explicit dispatch points. State-check caching and planning-failure handling are described in Section C.8.3.

In each controller iteration, b is the shared AF2 reservation ceiling in jobs, and u counts the jobs newly reserved so far in that iteration. ReserveAF2 inserts eligible evaluations at the front of the queue, updates u in place and returns newly reserved identifiers; $\mathcal{V}$ stores only the second call's identifiers. Both calls share b, with each call bounded by $\max\{0, b - u - n_{\text{AF2}}(\mathcal{W})\}$, where n_{AF2} counts running standardized AF2 jobs. Reservation limits, exceptions when one queue position is available, deep-stall restrictions and admission of evaluations to otherwise unused queue positions follow Section C.8.1.

bioRxiv preprint doi: https://doi.org/10.64898/2026.09.22.753604; this version posted September 24, 2026. The copyright holder for this preprint (which was not certified by peer review) is the author/funder, who has granted bioRxiv a license to display the preprint in perpetuity. It is made available under a CC-BY 4.0 International license.

Algorithm S1 T-REX campaign control and asynchronous execution. Queue admission and worker starts are distinct. Required AF2 evaluation follows a separate admission policy; endpoint analysis is performed separately.

Require: fixed target, action space and measurement rules; W workers; $T_{\max}$; reporting denominator H
Ensure: campaign archive $\mathcal{D}$ and endpoint throughput N_{SU}/H

Initialize the campaign archive $\mathcal{D}$, queue $\mathcal{Q}$, workers $\mathcal{W}$, state s and controller clock.
while elapsed campaign wall time $< T_{\max}$, checked at iteration entry **do**
  Apply family-availability rules; collect fresh outputs, completions and failures; update execution state.
  Refresh the progress checkpoint and s when due.
  $b \leftarrow \text{ReserveBudget}(\mathcal{D}, W)$; $u \leftarrow 0$.
  if $F(\mathcal{W}) > 0$ **then**
    Update s using the state check described above; remove AF2 reservations and queued generation/redesign jobs rejected by the queue-admission checks.
  end if
  If $s =$ `deep_stall`, set $b \leftarrow \min\{b, 1\}$.
  if $F(\mathcal{W}) > 0$ **then**
    $\text{ReserveAF2}(\mathcal{D}, \mathcal{Q}, \mathcal{W}, F(\mathcal{W}), b, u, s)$.
  end if
  $\text{DispatchReady}(\mathcal{D}, \mathcal{Q}, \mathcal{W})$.
  if $K(\mathcal{Q}) > 0$ and ($\mathcal{D}$ contains a target ResultRecord or $F(\mathcal{W}) > 0$) **then**
    $\mathcal{V} \leftarrow \text{ReserveAF2}(\mathcal{D}, \mathcal{Q}, \mathcal{W}, K(\mathcal{Q}), b, u, s)$.
    $K_* \leftarrow K(\mathcal{Q})$.
    if $K_* > 0$ **then**
      Retire eligible expired cards; $\mathbf{d} \leftarrow \text{ReplayAllocation}(\mathcal{D})$.
      $E \leftarrow \text{ReduceEvidence}(\mathcal{D}, \mathcal{Q}, \mathcal{W})$; archive E.
      $(\mathcal{H}, f_P) \leftarrow \text{Planner}(E, \mathcal{D})$.
      $\mathcal{J} \leftarrow \text{BuildCheck}(E, \mathcal{H}, \mathcal{D})$, including eligible initial jobs and candidates added by fixed rules.
      if $\mathcal{H} \neq \varnothing$ and $\mathcal{J} \neq \varnothing$ **then**
        $S \leftarrow \text{Supervisor}(E, \mathcal{H}, \mathcal{J}, \mathbf{d}, \mathcal{D})$.
      else
        Set S to an invalid, abstaining response; archive the record without an LLM call.
      end if
      $\mathcal{P} \leftarrow \text{ResolvePolicy}(E, \mathcal{H}, \mathcal{J}, S, f_P, \mathcal{D})$.
      if $\mathcal{P}$ permits a usable fresh global order of returned candidate identifiers **then**
        $\mathcal{L} \leftarrow \text{SelectGlobal}(\mathcal{J}, \mathcal{P}, K_*, E, \mathcal{D})$.
      else
        $\mathcal{L} \leftarrow \text{SelectQuota}(\mathcal{J}, \mathcal{P}, \mathbf{d}, K_*, E, \mathcal{D})$.
      end if
      Archive launch approvals and selection records; update earlier active hypotheses from their linked results.
      Update s from E; withdraw jobs in $\mathcal{V}$ invalidated by deep-stall restrictions.
      Append approved, eligible job identifiers from $\mathcal{L}$ to $\mathcal{Q}$.
      Apply the separate idle-capacity AF2 admission procedure to $\mathcal{Q}$.
    end if
  end if
  $\text{DispatchReady}(\mathcal{D}, \mathcal{Q}, \mathcal{W})$; wait for the configured polling interval or idle backoff.
end while
for all busy workers in $\mathcal{W}$, in pool order **do**
  Wait up to 600 s per worker; parse completion, or recover available results and terminate on timeout.
end for
Separate endpoint analysis: apply the endpoint-inclusion and qualification rules, recompute final Foldseek clusters and report N_{SU}/H.

bioRxiv preprint doi: https://doi.org/10.64898/2026.09.22.753604; this version posted September 24, 2026. The copyright holder for this preprint (which was not certified by peer review) is the author/funder, who has granted bioRxiv a license to display the preprint in perpetuity. It is made available under a CC-BY 4.0 International license.

C.2. Software and permitted job configurations

C.2.1. Software versions

Table S4 lists the software versions, source revisions, model checkpoints and configurations used in the reported campaigns and analyses. Baseline settings and adaptive initialization are described in Section B.4.

Table S4 | **Software, model checkpoints and analysis configurations.** Versions and revisions identify the primary generation, evaluation, clustering, LLM-serving and visualization tools. Post-hoc evaluator inputs, settings and score aggregation are detailed in Table S19; LLM serving is detailed in Section C.5.

Component	Version or revision	Use and configuration
Proteina-Complexa (Didi et al., 2026b)	Version 1.0.0; commit `5ae24b055d82`	Official Beam workflow: width 4, four branches, `nsamples=4`, maximum batch size 16, 400 diffusion steps, candidate-filter limit 1,000 and sequence deduplication. This generator configuration also defined the adaptive controllers' fixed initial Complexa launch.
BindCraft (Pacesa et al., 2025)	Commit `b971db42ba6e`; ColabDesign 1.1.3	Fixed reference: official `default_4stage_multimer.json` with `default_filters.json`. Adaptive controllers: repository-provided `default_4stage_multimer_mpnn_hardtarget.json` with the same filter file; the fixed initial launch requested two designs passing BindCraft's native filters, subject to controller runtime limits, after which only permitted configurations could vary. The fixed campaign specification supplied the target PDB, chains, hotspots and default binder-length range.
PyRosetta (BindCraft) (Chaudhury et al., 2010)	2026.03 releasequarterly; build `5e498f1409c6`	BindCraft structure relaxation and native interface diagnostics using `FastRelax` and `InterfaceAnalyzerMover`.
BoltzGen (Stark et al., 2025)	Version 0.3.2; commit `31d9d9b9c722`	Official `protein-anything` protocol: the fixed reference requested 10,000 designs with budget 30 per launch; the adaptive controllers' initial launch used 16 designs and budget 4 so evidence could inform later allocation decisions.
ProteinMPNN (Dauparas et al., 2022)	Default checkpoint `v_48_020`	Parent-bound binder-sequence redesign; alternative permitted checkpoints and parameter bounds are listed in Table S5. The repository revision was not recorded.
Common AF2 qualification (Jumper et al., 2021; Evans et al., 2021)	ColabDesign 1.1.1.1, tree `844c76c09355`; AF2-Multimer `model_1_multimer_v3`	Source of qualification pLDDT, normalized iPAE and binder scRMSD; three recycles, generated-complex initial coordinates, binder template without inter-chain template contacts and no dropout.
Foldseek (Van Kempen et al., 2024)	Revision `8dc75c74ad0e`	Binder-chain clustering at TM0.6; TM0.5 and TM0.8 were sensitivity analyses.
MMseqs2 (Steinegger and Söding, 2017)	Revision `76da68ad7577`	Binder-sequence clustering at 70% identity and 0.8 coverage.
SMAC3 (Lindauer et al., 2022)	Version 2.4.0; ConfigSpace 1.2.2	Official `RandomForest` model and expected-improvement (`EI`) acquisition implementations over the common eight-family action space and parameter bounds. Model fitting required at least eight completed observations and two distinct objective values. Settings: 10 trees, feature ratio $5/6$, minimum split/leaf sizes three, maximum depth 20, bootstrap sampling, no log transform and $\xi = 0$.
Planner and Supervisor LLM (Team, 2026)	`Qwen/Qwen3.6-27B-FP8`; model revision `ec4160bf26124fa57e6451d070ee0c459a36d5b7`	Both roles used the same checkpoint with distinct prompts and output schemas; call settings are given in Section C.5.1.
LLM serving (Kwon et al., 2023)	vLLM 0.19.0; PyTorch 2.10.0	One H100 GPU; 65,536-token context, GPU memory utilization 0.90 and thinking disabled. The `xgrammar` and `transformers` revisions were not retained.

Continued on next page

bioRxiv preprint doi: https://doi.org/10.64898/2026.09.22.753604; this version posted September 24, 2026. The copyright holder for this preprint (which was not certified by peer review) is the author/funder, who has granted bioRxiv a license to display the preprint in perpetuity. It is made available under a CC-BY 4.0 International license.

Table S4 (continued)

Component	Version or revision	Use and configuration
ESMFold2 (Candido et al., 2026)	ESM 3.3.0; Biohub revision 67838dc8ac76; model revision 1ebf0e3481a5	Post-hoc sequence-only complex prediction with biohub/ESMFold2; overall ipTM and pLDDT diagnostics (Table S19).
Boltz-2 (Passaro et al., 2025)	Boltz 2.2.1; revision cb04aeccdd48	Post-hoc sequence-only complex prediction in empty-MSA mode; confidence diagnostics rather than the affinity head (Table S19).
ipSAE and actif-pTM (Dunbrack Jr, 2025; Varga et al., 2025)	Vendored ColabDesign implementation (ipSAE); study implementation (actif-pTM)	Post-hoc AF2 interface-confidence diagnostics: arithmetic mean of directional ipSAE values and actifpTM weighted by inter-chain distogram probabilities for distances below 8 Å (Table S19).
PRODIGY (Xue et al., 2016)	prodigy-prot 2.4.0	Post-hoc predicted binding free energy ΔG (kcal mol^{-1}) and derived K_{D} (M) at 25 °C, using package defaults (Figure 2c; Table S19).
DSSP secondary-structure assignment (Kabsch and Sander, 1983; McGibbon et al., 2015)	MDTraj 1.11.0	compute_dssp with simplified=True; helix, strand and coil fractions. No separate DSSP executable was used.
Molecular visualization (Pettersen et al., 2021)	UCSF ChimeraX 1.11	Molecular views in Figure 5a,c.

C.2.2. *Action space and parameter bounds*

All adaptive controllers used the same eight action families: six generation families (Complexa beam search, best-of-n, Feynman–Kac steering and MCTS, BindCraft and BoltzGen), ProteinMPNN redesign and AF2 structure evaluation. An action family identifies the tool and, for Complexa, the search variant. A *generator root* groups generation families implemented by the same generator: the four Complexa variants share one root, while BindCraft and BoltzGen each form a separate root.

Each job specifies an action family, a configuration (model and hyperparameter settings) and, for redesign or evaluation, an archived parent design. A *route* groups jobs with the same generation or redesign family and configuration; redesign routes also distinguish the family and configuration that produced the parent design. For example, ProteinMPNN redesign of Complexa and BindCraft outputs forms different routes even when the ProteinMPNN settings match. Standardized AF2 evaluation is attributed to the route that produced its input; route outcomes and costs are defined in Section C.4.2.

The action families and parameter bounds in Table S5 were fixed before each campaign. The Planner and Supervisor proposed and prioritized jobs within this space, while newly available parent designs changed which candidates could be constructed (Section C.6). The space also permits diagnostic AF2 refolding with changed model, recycle or initialization settings. When eligible, these candidates follow the same selection and R/E/X start-accounting rules as generation and redesign jobs, but cannot supply common qualification measurements. Required standardized AF2 evaluation uses fixed settings and separate deterministic admission and ordering (Section C.8.1). The reported allocation analyses concern generation and redesign starts (Section D.3.2).

The per-launch limits in Table S5 constrain validated family-specific workload settings, expressed as counts or products of configuration values. For BindCraft, the controller field `max_trajectories` is forwarded as `number_of_final_designs` and specifies the requested number of designs passing BindCraft's native filters, not a cap on attempted trajectories. Native BindCraft acceptance is distinct from standardized qualification and SU counting. Actual trajectory and output counts must be read from execution records; controller runtime limits can terminate a job before the requested count is reached. These workload settings do not measure GPU hours and are not comparable across families.

bioRxiv preprint doi: https://doi.org/10.64898/2026.09.22.753604; this version posted September 24, 2026. The copyright holder for this preprint (which was not certified by peer review) is the author/funder, who has granted bioRxiv a license to display the preprint in perpetuity. It is made available under a CC-BY 4.0 International license.

Table S5 | **Permitted generation, redesign and evaluation action space.** Numeric intervals are validation bounds rather than a discretized grid. The eight families contain 103 configuration fields: 38 integer-valued ranges, 51 continuous ranges and 14 categorical fields, counting shared Complexa controls separately for each family. Parent-bound jobs require an archived structure. The three AF2 fields permit diagnostic refolding and are included in these counts. Standardized qualification uses the fixed settings shown; diagnostic refolds cannot supply common qualification measurements. For BindCraft, the controller field `max_trajectories` is forwarded as `number_of_final_designs`: a requested native accepted-design count, not a cap on attempted trajectories.

Action family or role	Registered configuration fields and allowed ranges	Per-launch limit or policy
**A. Generation families**		
Complexa beam	`beam_width`: 2–16; `n_branch`: 2–8; `nsamples`: 1–8; `nsteps`: 100–500; `batch_size`: 1–16; `sc_scale_noise`: 0.05–0.50; `refinement_algorithm`: empty or `sequence_hallucination`; `n_greedy_iters`: 0–30; `enable_greedy_optimization`: false/true; `greedy_percentage`: 1.0–10.0; `filter_samples_limit`: 1–1000; plus the shared reward-term weights below.	`nsteps` × `nsamples` × `beam_width` × `n_branch` ≤ 65,536
Complexa best-of-n	`replicas`: 1–8; `nsamples`: 1–16; `nsteps`: 100–500; `batch_size`: 1–16; `sc_scale_noise`: 0.05–0.50; empty or `sequence_hallucination` refinement; `n_greedy_iters`: 0–30; greedy enable flag; `greedy_percentage`: 1.0–10.0; `filter_samples_limit`: 1–1000; plus the shared reward-term weights below.	`nsteps` × `nsamples` × `replicas` ≤ 65,536
Complexa FK steering	`beam_width`: 2–16; `n_branch`: 2–8; `temperature`: 0.05–0.50; `nsamples`: 1–8; `nsteps`: 100–500; `batch_size`: 1–16; `sc_scale_noise`: 0.05–0.50; empty or `sequence_hallucination` refinement; greedy controls and `filter_samples_limit`: 1–1000; plus the shared reward-term weights below.	`nsteps` × `nsamples` × `beam_width` × `n_branch` ≤ 65,536
Complexa MCTS	`n_simulations`: 5–80; `exploration_prob`: 0.10–0.80; `exploration_constant`: 0.50–2.0; `nsteps`: 100–500; `nsamples`: 1–8; `batch_size`: 1–16; `sc_scale_noise`: 0.05–0.50; empty or `sequence_hallucination` refinement; greedy controls and `filter_samples_limit`: 1–1000; plus the shared reward-term weights below.	`nsteps` × `nsamples` × `n_simulations` ≤ 131,072
All Complexa families: generator-internal reward weights	`reward_i_pae_weight`, `reward_min_ipae_weight`: $[-2, 0]$; `reward_plddt_weight`, `reward_min_ipsae_weight`, `reward_avg_ipsae_weight`, `reward_max_ipsae_weight`, `reward_i_ptm_weight`: $[0, 2]$; `reward_i_con_weight`: $[-2, 2]$.	Weights steer search, remain fixed within each job and do not enter the workload product
BindCraft	`max_trajectories`: 2–64; binder-length bounds `target_length_min`: 40–120 and `target_length_max`: 60–200; `weights_plddt`: 0.05–0.40; `weights_pae_inter`: 0.05–0.40; `weights_iptm`: 0.02–0.20; `weights_helicity`: $[-0.80, 0.20]$; `soft_iterations`: 20–200; `hard_iterations`: 1–20; `greedy_iterations`: 5–40; `num_seqs`: 4–64; `mpnn_fix_interface`: false/true.	Requested native accepted-design count ≤ 64; not a trajectory cap
BoltzGen	`protocol=protein-anything`; `num_designs`: 8–128; `budget`: 1–16; `diffusion_batch_size`: 1–16; `step_scale`: 0.5–2.0; `noise_scale`: 0–1.0.	`num_designs` × `budget` ≤ 256
**B. Parent-bound redesign and structure evaluation**		
ProteinMPNN redesign	`num_seq_per_target`: 1–32; `sampling_temp`: 0.05–0.50; `model_name`: `v_48_002`, `v_48_010`, `v_48_020` or `v_48_030`; `backbone_noise`: 0–0.30; `omit_AAs`: `X`, `C`, `CP`, `CW`, `FWY`, `G`, `P`, `GP`, `DE` or `KR`.	Up to 32 sequences; additional registry ceiling: 2,048 sequences; requires parent
AF2 structure evaluation	Registry: `num_recycles=0–6`; `use_initial_guess=0` or 1; `model_names` selects one multimer-v3 model 1–5 or the consecutive ensemble 1–2, 1–3, 1–4 or 1–5. The standardized role fixes `model_names=model_1_multimer_v3`, `num_recycles=3` and `use_initial_guess=1`.	Model count × recycles; standardized settings fixed as shown; requires parent

C.3. Campaign archive and runtime state

The campaign archive stores job results, settings, proposals and decisions, with links to structures, score files and logs. Each primary campaign retained nine append-only JSON Lines (JSONL) streams, with one JSON object per line, grouped by role in Figure S1a. Scientific measurements in the *EvidenceSummary* come from recorded job results. The controller combines these records with

bioRxiv preprint doi: https://doi.org/10.64898/2026.09.22.753604; this version posted September 24, 2026. The copyright holder for this preprint (which was not certified by peer review) is the author/funder, who has granted bioRxiv a license to display the preprint in perpetuity. It is made available under a CC-BY 4.0 International license.

information on queued and running jobs, designs awaiting AF2 evaluation, worker availability and time remaining for new launches (Section C.8).

Results and selections. The *ResultRecords* stream stores generation, redesign and evaluation outputs or failures, with measurements, worker cost, status and parent links. Each record has a unique identifier (`result_id`); one job can produce several records, including outputs collected before it finishes or a record retaining cost without a usable design (Section C.8.2). The *Panel selections* stream stores nominated design identifiers and their selection checks when emitted.

Within a *ResultRecord*, the parsers store measurements in `metrics`. The `bins` field stores annotations such as measurement sources, evaluation roles and chain labels; `artifacts` stores output paths and chain identifiers. A stored structure may still require standardized AF2 evaluation. Parent and action identifiers link that evaluation to the generation or redesign route that produced its input. The `tick_id` identifies the planning update associated with the job's start, so its results can first appear in a later *EvidenceSummary*. Online cluster assignments can change as results accumulate; previously archived `bins` remain unchanged (Section C.4.2).

Evidence and decisions. The *EvidenceSummary* stream stores cumulative qualification and online SU counts, recent results, outcomes and costs by route, clustering status and structural duplication, and recent planning decisions at each planning update (Section C.4; JSON example in Section D.2.1). The *HypothesisCards* stream stores Planner hypotheses, proposed tests and rule-based feedback; updates append records under the same hypothesis identifier (Section C.9). *ActionCandidates* stores constructed and checked candidate jobs. *SupervisorDecisions* stores allocation proportions, an optional ranking of a candidate subset, the capacity information supplied to the Supervisor, rationale and selection checks. *LLM-call records* retain model identifiers, prompt hashes, response validation status, latency, token use and failure or fallback information, together with selected prompt fields; full prompt text is not retained.

Launch and execution. The *LaunchDecisions* stream records deterministic approval or rejection after selection checks. Approval permits queue admission but does not establish that a job started. The *DispatchRecords* stream separately records confirmed starts, failed start attempts, temporary deferrals and failures to collect job outputs, with worker identifiers, attempt numbers and reasons (Section C.8.2). Post-start outputs, measurements and costs are stored as *ResultRecords*. Current queue membership and running jobs are tracked separately from these event records.

bioRxiv preprint doi: https://doi.org/10.64898/2026.09.22.753604; this version posted September 24, 2026. The copyright holder for this preprint (which was not certified by peer review) is the author/funder, who has granted bioRxiv a license to display the preprint in perpetuity. It is made available under a CC-BY 4.0 International license.

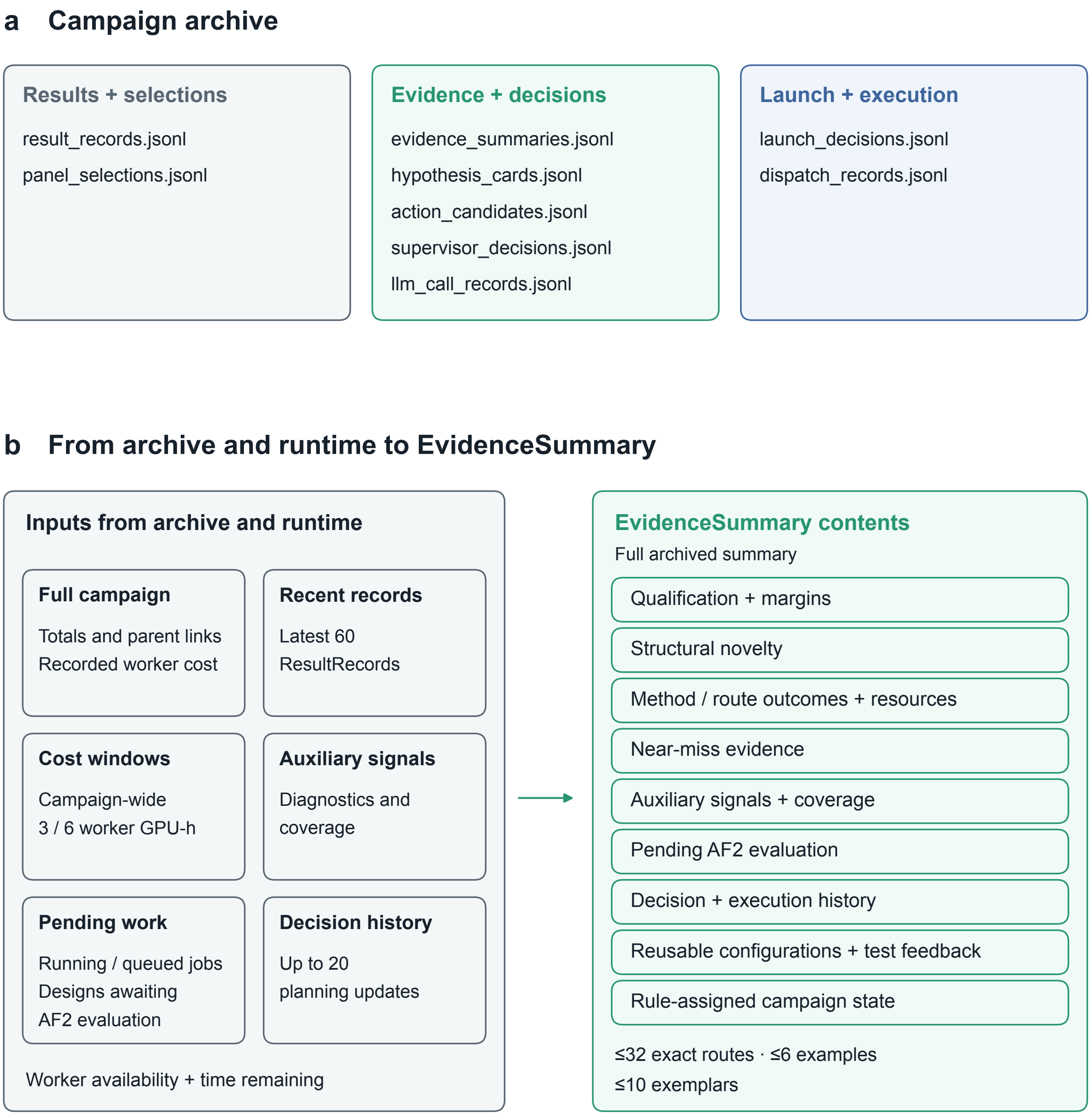

Figure S1 | **Campaign archive and *EvidenceSummary* construction.** **a**, The nine append-only record streams retained in the primary campaigns are grouped by role (Section C.3). ResultRecord stores measurements, parent links, worker cost, status and output paths. Raw logs and files remain in the run directory; LLMCallRecord retains a prompt hash and compact audit rather than full prompt text. **b**, Deterministic rules combine archived records with information on current jobs and resources to construct the full archived *EvidenceSummary* (arrow). The 3- and 6-worker-GPU-h windows use campaign-wide recorded worker cost. Figure S2 shows the smaller input supplied to the LLMs. Final qualification and SU counts are recomputed separately after applying endpoint-inclusion rules (Section B.3). Gray indicates results, selections and reduction inputs; green, evidence/decision records and summary contents; and blue, launch/execution records.

bioRxiv preprint doi: https://doi.org/10.64898/2026.09.22.753604; this version posted September 24, 2026. The copyright holder for this preprint (which was not certified by peer review) is the author/funder, who has granted bioRxiv a license to display the preprint in perpetuity. It is made available under a CC-BY 4.0 International license.

C.4. EvidenceSummary construction and derived measures

At each planning update, the controller combines archived results with queued and running jobs, designs awaiting AF2 evaluation and available worker capacity to construct an *EvidenceSummary*. It reports cumulative qualification and online SU counts, recent results, and route outcomes and costs (Figure S1b). Section C.5.2 describes the smaller input supplied to the LLMs; Section D.2.1 provides a JSON example.

C.4.1. Evidence contents and record windows

Measurements and missing values. Qualification summaries report median measurements and median distances from the qualification cutoffs, counts of pass, near-pass and failed measurements, and paired-criterion outcomes (Section B.1). In archive field names, `strict` denotes computational qualification. Online Foldseek TM0.6 assignments and duplication measures include clustering status and coverage. Auxiliary confidence, interface and geometry measurements retain their source and availability. Missing measurements are not imputed or treated as failures; `metric_availability` identifies which measurements are present.

Record windows and minimum observation counts. Cumulative qualification, SU and parent-history fields use all observed results. Recent qualification summaries and combinations of criterion outcomes use the latest 60 ResultRecords (Section C.3). General auxiliary summaries combine the latest 40 records per family with the latest 60 overall, including each record only once. They require at least three finite observations per measurement; the route-level auxiliary score has a separate requirement (Section C.4.3). The 3- and 6-worker-GPU-h windows select records backward from the newest result until their summed recorded cost reaches the specified amount, including the record that reaches or crosses it. If less cost has accumulated, all records are used. Each campaign-wide window is shared across routes.

The recent duplicate fraction is

$$f_{\mathrm{dup}} = 1 - \frac{N_{\mathrm{clusters}}}{N_{\mathrm{assigned}}}, \qquad N_{\mathrm{assigned}} > 0, \tag{S3}$$

where N_{assigned} counts records with an online Foldseek TM0.6 cluster assignment among the latest 60 ResultRecords, and N_{clusters} counts their distinct clusters. The fraction is unavailable when $N_{\mathrm{assigned}} = 0$. This recent duplication measure is separate from final qualified-binder clustering.

Near misses and counting units. A *deficit* is the nonnegative distance by which a measurement misses its qualification cutoff. The *near-pass tolerances* are 5 pLDDT points, 0.05 normalized iPAE and 0.3 Å scRMSD. A near miss has finite values for all three measurements and fails qualification, with at most one criterion outside its tolerance and no failed criterion more than ten corresponding tolerances from its cutoff. Near misses are clustered separately from qualified designs and receive no SU credit.

The campaign-state input `near_miss_count` counts distinct near-miss Foldseek clusters in the latest 60 ResultRecords, subject to the reliability checks in Section C.4.2. Method summaries count cumulative near-miss clusters; their recent near-miss yield counts clusters first observed in the latest 60 ResultRecords and attributed to each family. In contrast, `near_miss_count` and `near_miss_recent` in exact-route rows count individual near-miss ResultRecords; family rows in `route_values` sum these route counts. The same field name therefore has different counting units in campaign and route summaries.

bioRxiv preprint doi: https://doi.org/10.64898/2026.09.22.753604; this version posted September 24, 2026. The copyright holder for this preprint (which was not certified by peer review) is the author/funder, who has granted bioRxiv a license to display the preprint in perpetuity. It is made available under a CC-BY 4.0 International license.

Work awaiting completion and worker availability. The summary includes pending AF2 evaluations (`diagnostic_chain_backlog`), queued and running jobs (`pending_family_load`), failures, timeouts, evaluation-role outcomes and available workers. It also identifies usable redesign parents and parent–child lines flagged for non-improvement. The `dispatch_realization` and `execution_realization` fields distinguish proposals, selections and starts (Section C.3). Section D.2.1 illustrates how measurements, missing values and pending work appear in the summary and the LLM input.

Method summaries and reusable evidence. The `method_health` summary combines family-level attempts, completions, failures, costs and qualification/novelty outcomes to distinguish untried, delayed and unproductive methods. Its recent rates use new SU gains in the latest 60 ResultRecords. Direct rates divide those gains by the family's recorded cost in that window. Rates attributed through downstream evaluation also include its recorded cost and required ancestor costs outside the window, counting each ancestor once within the family. These method summaries are distinct from the family aggregates in `route_values`; they supply the recent family rates used in selection and concurrency checks (Sections C.7.2 and C.8.2).

A previously tested configuration summary (`recipes`) groups results by action family, configuration and outcome category, retaining counts, median measurements and representative result identifiers. Strategy-outcome summaries (`strategy_feedback`) retain outcomes, costs, qualification failures, near misses and parent–child history for each strategy, including negative and partial results. These are observations for reuse, not guarantees of future success; Section D.2.3 gives worked examples.

Routes, examples and planning history. The `route_values` summary records outcomes, costs, pending evaluation, quality scores and recent or cumulative SU/GPU-h for each route, with separately labeled family aggregates. Section C.4.2 defines route cost and SU attribution. Illustrative result records (`examples`) link individual outcomes to qualification measurements, deficits and parents where available. Configuration-linked designs (`exemplars`) are selected qualified or near-miss binders with their producing configurations and parent links, providing concrete evidence for follow-up tests. Each summary retains up to twenty planning-update summaries in `recent_ticks_history`; the LLM input retains ten (Section C.5.2). HypothesisCards, archived separately, describe proposed tests (Sections C.3 and C.9).

C.4.2. Online SU attribution and compute accounting

Route attribution and cost. Routes group jobs as defined in Section C.2.2. Standardized AF2 evaluation is attributed to the generation or redesign route that produced its input. Each online qualified cluster adds one SU to the route that produced its first qualified member in archive order. Other routes can contribute qualified members without another SU credit. Final attribution instead follows the job that produced the fixed representative (Section B.2).

Route cost includes generation or redesign, required evaluation and the recorded costs of ancestor designs. Each ancestor ResultRecord is charged once within a route, but can be charged to several routes; summing route costs therefore does not give campaign cost. A multi-output job's cost is divided among its ResultRecords. Recent route costs use the campaign-wide windows in Section C.4.1, including required ancestor costs even when the ancestor's record precedes the window. Table S6 distinguishes these costs from the reporting budget and other online time measures.

bioRxiv preprint doi: https://doi.org/10.64898/2026.09.22.753604; this version posted September 24, 2026. The copyright holder for this preprint (which was not certified by peer review) is the author/funder, who has granted bioRxiv a license to display the preprint in perpetuity. It is made available under a CC-BY 4.0 International license.

Table S6 | **Time and cost quantities used for reporting and control.** GPU-hours are time-accounting units, not measurements of GPU utilization or floating-point operations. These quantities have different definitions and must not be substituted for one another.

Quantity	Definition	Use
Primary reporting budget	Fixed $H = 144$ H100 worker-slot hours per primary campaign; each allocated worker is counted once.	Final N_{SU}/H; excludes the separate LLM server and generator-only post-cutoff evaluation under primary accounting.
Elapsed worker-slot time	Elapsed campaign wall time multiplied by configured worker count (`worker_wall_gpu_h_total`).	Campaign throughput objective supplied in the LLM input; includes elapsed idle slot time.
Recorded worker cost	Sum of recorded `ResultRecord.gpu_h` values (`worker_gpu_h_total`); includes partial outputs from running jobs, with newly accrued time divided among fresh ResultRecords.	Cumulative compute evidence and the cold-start rule; not the fixed reporting denominator.
Recent classifier exposure	Sum of record costs in the latest 60-result window; the productive-rate denominator is the larger of this sum and 0.5 GPU-hours.	Rate paired with the recent online SU gain (Section C.4.2).
Charged time without new SU	Recorded worker cost plus elapsed time on running worker slots, minus the reference cost defined in Section C.4.2; negative values replaced with zero. Running-slot time is omitted on updates recognizing new SU.	Stalled-state escalation and safeguards based on time without a new SU; not elapsed wall time. The busy-slot term can overlap partial-result time already charged.
Exact-route cost	Production, required evaluation and charged redesign ancestors, each counted once within a route; recent route views use the campaign-wide 3- and 6-GPU-hour result windows before route aggregation.	Candidate-specific efficiency; shared ancestors make costs non-additive across routes.

New online SU and clustering reliability. Recent gain counts online SU first represented in the latest 60 ResultRecords. At each update, the controller counts qualified clusters in all records and in records preceding that window. It subtracts the earlier-record count from the full count, replacing negative values with zero. Both counts use current cluster assignments, so the calculation does not compare summaries from different updates.

Productive-state classification requires Foldseek clustering status `ok`, coverage ≥ 0.999, an available duplicate fraction and no reported fallback from binder-chain to whole-complex clustering. Here, coverage is the fraction of qualified ResultRecords assigned to qualified structural clusters, not the number of clusters. Section C.4.5 specifies the additional progress and rate conditions. A positive near-miss count requires separate clustering with status `ok` or `cached_ok` and coverage ≥ 0.999 among near-miss records. Otherwise, the classifier receives zero while the original count, examples and clustering status remain available. This substitution disables the near-miss decision input; it does not establish that no near misses occurred.

Time without new online SU. At each update, the controller reconstructs a reference cost by scanning results in archive order. Whenever a qualified design is the first qualified member of its cluster encountered so far, the reference becomes the cumulative recorded worker cost through that result; it is zero before the first SU. If an update reports more online SU than any previous update,

bioRxiv preprint doi: https://doi.org/10.64898/2026.09.22.753604; this version posted September 24, 2026. The copyright holder for this preprint (which was not certified by peer review) is the author/funder, who has granted bioRxiv a license to display the preprint in perpetuity. It is made available under a CC-BY 4.0 International license.

the reference is instead the current recorded worker cost.

The stall-time signal is current recorded worker cost plus elapsed time on running worker slots, minus that reference, with negative values replaced by zero. Running time is summed from each running job's start and omitted on updates recognizing new SU. Partial-output costs already recorded are not deducted from this term, so time can be counted twice (Section C.8.2). This signal detects stalls using different accounting from the reporting budget (Table S6).

C.4.3. Online quality and auxiliary scores

Online quality score and route summaries. For a qualified design, the margins below measure how much better its measurements are than the qualification cutoffs. Each margin is divided by the corresponding near-pass tolerance:

$$\begin{aligned} u_p &= \max\{0, (\text{pLDDT} - 90)/5\}, \\ u_i &= \max\{0, (7/31 - \text{iPAE})/0.05\}, \\ u_s &= \max\{0, (1.5 - \text{scRMSD})/0.3\}. \end{aligned}$$

Each value contributes at most 3. Their sum is divided by 9 to give the design score q, between 0 and 1:

$$q = \{\min(u_p, 3) + \min(u_i, 3) + \min(u_s, 3)\}/9.$$

Within each route and online Foldseek cluster, the qualified design with all three measurements and the highest q is selected. Each cluster contributes one quality observation, even if another route received its SU credit. A later, higher-scoring design in the same cluster replaces that observation rather than adding one.

The route's qualified-cluster count is the number of selected designs. The median and 25th percentile of their q values are stored as `strict_quality_median` and `strict_quality_p25`. The percentile uses inclusive linear interpolation. With one selected design, both equal its score; with none, both are missing. Criterion-specific margin summaries use these same designs. A family summary copies the quality statistics of its route with the highest 25th percentile, breaking ties by the median and then the qualified-cluster count.

These summaries include all currently observed qualified clusters in a route, not only those in a recent window. The Planner and Supervisor receive them to compare routes with similar recent SU throughput (Section C.7). An increase in the median or 25th percentile can also permit additional standardized AF2 evaluations (Section C.8.1). Final attribution, post-hoc analyses and qualification-margin sensitivity instead use $m_{\min}$ (Sections B.2 and D.1.4). Neither score changes qualification or gives an unqualified design SU credit.

Auxiliary diagnostic-improvement score. This score summarizes whether auxiliary measurements meet or approach their quality thresholds, or have improved relative to earlier results. Table S7 gives the source-specific measurements, thresholds, near-pass tolerances, weights and acceptance-reference limits. These references do not add qualification criteria.

For each route and measurement, recent observations are its finite values in the campaign-wide 6-GPU-hour result window (Section C.4.1); prior observations are its finite values in earlier records. A measurement is used in this score with at least two recent observations, whether or not they meet the quality threshold. A single recent observation is used only if it meets that threshold.

bioRxiv preprint doi: https://doi.org/10.64898/2026.09.22.753604; this version posted September 24, 2026. The copyright holder for this preprint (which was not certified by peer review) is the author/funder, who has granted bioRxiv a license to display the preprint in perpetuity. It is made available under a CC-BY 4.0 International license.

The deficit is how far a measurement misses its quality threshold, or zero if the threshold is met. Dividing it by the corresponding near-pass tolerance gives the normalized deficit. Two values are then calculated for each measurement:

Current quality. Among the recent observations, those meeting the quality threshold contribute one, those missing it by no more than the tolerance contribute one-half, and the rest contribute zero. The sum of these contributions is divided by the number of recent observations.

Improvement. The controller compares the median normalized deficit in prior and recent observations. Only a decrease contributes: the decrease is divided by the larger of one and the prior median. Without prior observations, this term is zero.

For each measurement, the larger of these two values is multiplied by its Table S7 weight: 1.0 if a registered parameter directly addresses that measurement, or 0.6 otherwise. The route score is the largest weighted value across its measurements. It is zero if observations are insufficient or if no measurement yields a positive value. Zero therefore cannot distinguish missing evidence from observed values that yield no positive score, and does not by itself establish deterioration.

Scores lie between zero and one and are stored rounded to three decimal places. An action-family summary uses the largest score among its exact routes. The score can support further evaluation (Section C.8.1) or prevent premature route deferral (Section C.7), but cannot qualify a design or replace new-SU throughput as the ranking objective.

The general auxiliary summaries are separate from this route score. They require at least three finite observations and also check the acceptance-reference limits in Table S7. Values failing a limit are counted as failures even if they fall within the near-pass tolerance of the quality threshold. A dash means that no acceptance-reference limit applies. This additional check is not used in the route score.

Table S7 | **Reference values and weights for auxiliary measurements.** Quality thresholds and near-pass tolerances define the auxiliary diagnostic-improvement score; a near pass misses the quality threshold by no more than the tolerance. Acceptance-reference limits additionally constrain the general auxiliary summaries, not the route score. Limits use the direction indicated in the *Better* column; a dash means no additional limit. A weight of 1.0 identifies a measurement with a registered directly relevant parameter; 0.6 denotes corroborating evidence. These source-specific references cannot establish common qualification or SU credit.

Source	Measurement and scale	Better	Acceptance reference	Quality threshold	Near-pass tolerance	Weight
BindCraft	Interface energy (Rosetta energy units)	Lower	0	−56	9	0.6
	Shape complementarity	Higher	0.60	0.65	0.04	0.6
	Interface hydrogen-bond count	Higher	3	5	2	0.6
	Buried unsatisfied hydrogen-bond count	Lower	4	2	1	0.6
	Buried surface area (Å^2)	Higher	–	1,650	330	1.0
	Binder pLDDT (0–1)	Higher	0.80	0.90	0.03	1.0
	Hotspot RMSD (Å)	Lower	6.0	2.0	0.6	0.6
	Binder pTM	Higher	–	0.79	0.05	0.6
Complexa	ipTM	Higher	0.50	0.75	0.27	1.0
	Mean ipSAE	Higher	–	0.55	0.25	1.0
	Maximum ipSAE	Higher	–	0.60	0.26	1.0
	Minimum normalized iPAE	Lower	7/31	0.07	0.21	1.0
BoltzGen	Design-to-target ipTM	Higher	0.50	0.60	0.09	1.0
	Interface ipTM	Higher	0.50	0.60	0.09	0.6
	Design pTM	Higher	0.70	0.80	0.05	0.6
	Minimum design-to-target PAE (Å)	Lower	15	10	5	0.6

bioRxiv preprint doi: https://doi.org/10.64898/2026.09.22.753604; this version posted September 24, 2026. The copyright holder for this preprint (which was not certified by peer review) is the author/funder, who has granted bioRxiv a license to display the preprint in perpetuity. It is made available under a CC-BY 4.0 International license.

C.4.4. *Route status rules*

Each route has a general status (`status`) used for evaluation admission and route deferral, and a recent-productivity status (`marginal_status`) distinguishing continuing, delayed and declining yield. Both return the first matching rule below and identify pending evaluation separately. Family aggregates use the same rules, except that the 32-completion test is disabled and no qualified-cluster duplicate fraction is supplied. These statuses describe routes or families; Section C.4.5 defines whole-campaign states.

Window definitions. The *60-record window* contains the latest 60 campaign ResultRecords; the *3- and 6-GPU-hour windows* use campaign-wide recorded cost (Section C.4.1). A gain is new online SU credited to a route within the stated window. Recent near misses are individual near-miss records in the 60-record window, subject to the clustering checks in Section C.4.2. Route cost includes the applicable ancestor costs; lifetime rates use the full observed history.

Duplicate measures. Qualified/SU is the route's qualified-record count divided by its credited SU count; the ratio is unavailable when the SU count is zero. The route duplicate fraction uses Eq. (S3), with N_{assigned} and N_{clusters} counted over the route's cumulative qualified cluster-assigned records. A duplicate warning means qualified/SU ≥ 8 or duplicate fraction ≥ 0.80. Missing duplicate measures cannot satisfy their threshold tests.

Comparison rate. The best mature rate is the largest lifetime SU/GPU-h rate among exact routes with at least two credited SU and two route GPU-hours; zero if none qualifies. Missing rates are treated as zero only for the rule comparisons below.

AF2 structure-evaluation rows. AF2 evaluation rows bypass the generation/redesign classifications below. Standardized evaluation carries the archive label `plumbing`; the code reserves `advisory` for other evaluation roles, including an intentional refold of a parent model.

General route status (`status`).

1. `untried`: No positive accumulated route cost.
2. `awaiting_score_conversion`: No credited SU and at least one promising valid output awaiting standardized AF2 evaluation. Admission signals are defined in Section C.8.1.
3. `collapse_risk`: Either: a duplicate warning, at least one credited SU, at least eight qualified records and no 60-record or 6-GPU-hour SU gain; or qualified/SU ≥ 8, lifetime rate ≤ 0.30, and either no 6-GPU-hour gain or a positive 60-record gain.
4. `defer`: Either: neither a lifetime SU nor a recent near miss, with at least 3 route GPU-hours or 32 completed ResultRecords; or a best mature rate ≥ 0.50, at least 3 route GPU-hours, a lifetime rate below 35% of that best rate, and no 60-record gain, 6-GPU-hour gain or recent near miss. Completed ResultRecords here have `ok` or `no_artifacts` status; one job can produce several records.
5. `observed` (stale): At least one lifetime SU, but no 60-record gain, 6-GPU-hour gain or recent near miss, with at least 1 route GPU-hour in the 60-record window.
6. `diversify`: At least one lifetime SU and either qualified/SU ≥ 4 or duplicate fraction ≥ 0.55.

bioRxiv preprint doi: https://doi.org/10.64898/2026.09.22.753604; this version posted September 24, 2026. The copyright holder for this preprint (which was not certified by peer review) is the author/funder, who has granted bioRxiv a license to display the preprint in perpetuity. It is made available under a CC-BY 4.0 International license.

7. `promote`: At least one lifetime SU, a positive best mature rate and a lifetime rate at least 80% of that comparator. With no 60-record SU gain, qualified/SU ≥ 8 or duplicate fraction ≥ 0.70 prevents promotion.
8. `healthy`; otherwise `observed`: After the preceding rules, at least one lifetime SU or a recent near miss gives `healthy`; otherwise `observed`.

Recent-productivity status (`marginal_status`).

1. `untried`; `awaiting_score_conversion`: Apply the same first two tests as the general-status classification.
2. `under_tested`; otherwise `dry_low_quality`: For a route with zero lifetime SU, `under_tested` applies while accumulated route cost is below 3 GPU-hours and completions are below 32 ResultRecords, or while a recent near miss exists. Otherwise use `dry_low_quality`.
3. `productive_but_duplicate`: A duplicate warning and an SU gain in either the 60-record or 3-GPU-hour window.
4. `delayed_productive`; duplicate variant: An SU gain in the 6-GPU-hour window but none in either the 60-record or 3-GPU-hour window. A duplicate warning changes the label to `delayed_productive_duplicate`.
5. `dry_duplicate`: A duplicate warning, no 3-GPU-hour or 6-GPU-hour SU gain and recent rate decay: at least 1 route GPU-hour within the 3-GPU-hour window, with the larger SU/GPU-h rate from the 60-record and 3-GPU-hour windows below 35% of the positive lifetime rate.
6. `productive`: An SU gain in either the 60-record or 3-GPU-hour window.
7. `dry`; otherwise `observed`: The same recent-rate-decay test and no 6-GPU-hour SU gain give `dry`; all remaining routes are `observed`.

C.4.5. Campaign-state rules

The controller deterministically assigns one of seven campaign states from worker cost, recent results and new SU, duplication, near misses, criterion-specific deficits and time without new SU. State labels guide priorities and fallback allocation (Section C.7.1). `low_evidence` favors gathering more evidence; `productive` favors continuing productive routes; `productive_duplicate` adds diversification; and `strict_duplicate_collapse` favors diversification or alternative generator configurations. `rescue_rich` directs **Rescue** toward the limiting criterion, while `stalled` favors **Rescue** or **Explore** over exploitation and `deep_stall` strongly deprioritizes unproductive routes. Labels neither change qualification nor prescribe a particular job.

Criterion concentration is computed separately from the near-miss count. For criterion a, let d_a be its median nonnegative qualification deficit among available measurements in the latest 60 ResultRecords (`axis_stats`), and b_a its near-pass tolerance (Section C.4.1). The classifier uses

$$C_{\text{axis}} = \frac{\max_a (d_a/b_a)}{\sum_a (d_a/b_a)},$$

with value zero when no positive median deficit is available. Missing medians do not contribute. Thus the 0.50 cutoff below describes the concentration of scaled median deficits, not the fraction of near-miss designs failing one criterion. Scaling makes deficits in pLDDT, normalized iPAE and scRMSD comparable for this diagnostic.

Decision order and cold start. Cumulative recorded worker cost below 1.0 worker GPU-h or fewer than three recent ResultRecords returns `low_evidence`. Otherwise, the first matching rule

bioRxiv preprint doi: https://doi.org/10.64898/2026.09.22.753604; this version posted September 24, 2026. The copyright holder for this preprint (which was not certified by peer review) is the author/funder, who has granted bioRxiv a license to display the preprint in perpetuity. It is made available under a CC-BY 4.0 International license.

applies in this order: structural duplicate collapse; productive; productive with duplication; near-miss enrichment, with possible deep-stall escalation; stalled or deep stall; and default low evidence. This classification rule is separate from the initial-launch policy (Section B.4).

Structural duplicate collapse. This state requires reliable clustering as defined in Section C.4.2, at least 40 qualified designs, at least two SU clusters, at least eight qualified designs per SU on average, cumulative SU/GPU-h no greater than 0.30 and a recent SU gain no greater than one. When available, the ratio of Foldseek TM-score 0.8 to TM-score 0.6 cluster counts within the same recent qualified-design window must also be no greater than 1.30.

Productive and near-miss states. Productivity requires reliable clustering, an available duplicate fraction, a positive recent online SU gain and at least 0.04 new SU per worker GPU-h. This rate divides the 60-record gain by recorded worker cost in that window, with a minimum denominator of 0.5 GPU-h (Table S6). A duplicate fraction below 0.60 gives `productive`; otherwise it gives `productive_duplicate`. Near-miss enrichment requires at least three near-miss clusters in the 60-record window and $C_{\text{axis}} \geq 0.50$. If reached, this branch returns `deep_stall` after at least 12.0 worker GPU-h without new SU, and `rescue_rich` otherwise.

Stalled states. If no earlier rule applies, the stalled branch is entered when recent online SU gain is zero, clustering is unreliable, or the largest cluster contains at least 75% of assigned structures in the 60-record window without a productive rate. It returns `deep_stall` after at least 12.0 worker GPU-h without new SU and `stalled` otherwise. Missing stall-time information cannot trigger escalation; Section C.4.2 defines this signal. The 12.0-worker-GPU-h test does not override the earlier cold-start, collapse or productive rules. These fixed rules were calibrated as described in Section B.4; archived state labels describe their outcomes in the reported campaigns.

C.5. Language-model inputs, outputs and call handling

C.5.1. Model and call settings

Both roles used a local `Qwen3.6-27B-FP8` checkpoint served by vLLM on one H100 GPU; Table S4 specifies the model revision and software versions. The context limit was 65,536 tokens and GPU memory utilization was 0.90, with `enable_thinking=false` and `--trust-remote-code`. Calls used `max_tokens=3072`, `timeout_s=90` and OpenAI-client `max_retries=1`. Planner temperature was 0.2 with `prompt_variant=default`; Supervisor temperature was 0.0. Zero temperature did not ensure identical responses; Table S15, part B, reports controlled reruns.

Confidence below 0.55 was recorded without rejecting an otherwise valid response. Supervisor confidence below 0.65 could activate allocation bounds (Section C.7.1); this was a separate use of confidence. Each call recorded the model identifier, a 16-character SHA-256 prompt-hash prefix, output-format version, parse status, failure reason when present, and input/output token counts. These records distinguish LLM calls from deterministic advisory checks (Section C.5.4); Section C.3 describes their storage.

bioRxiv preprint doi: https://doi.org/10.64898/2026.09.22.753604; this version posted September 24, 2026. The copyright holder for this preprint (which was not certified by peer review) is the author/funder, who has granted bioRxiv a license to display the preprint in perpetuity. It is made available under a CC-BY 4.0 International license.

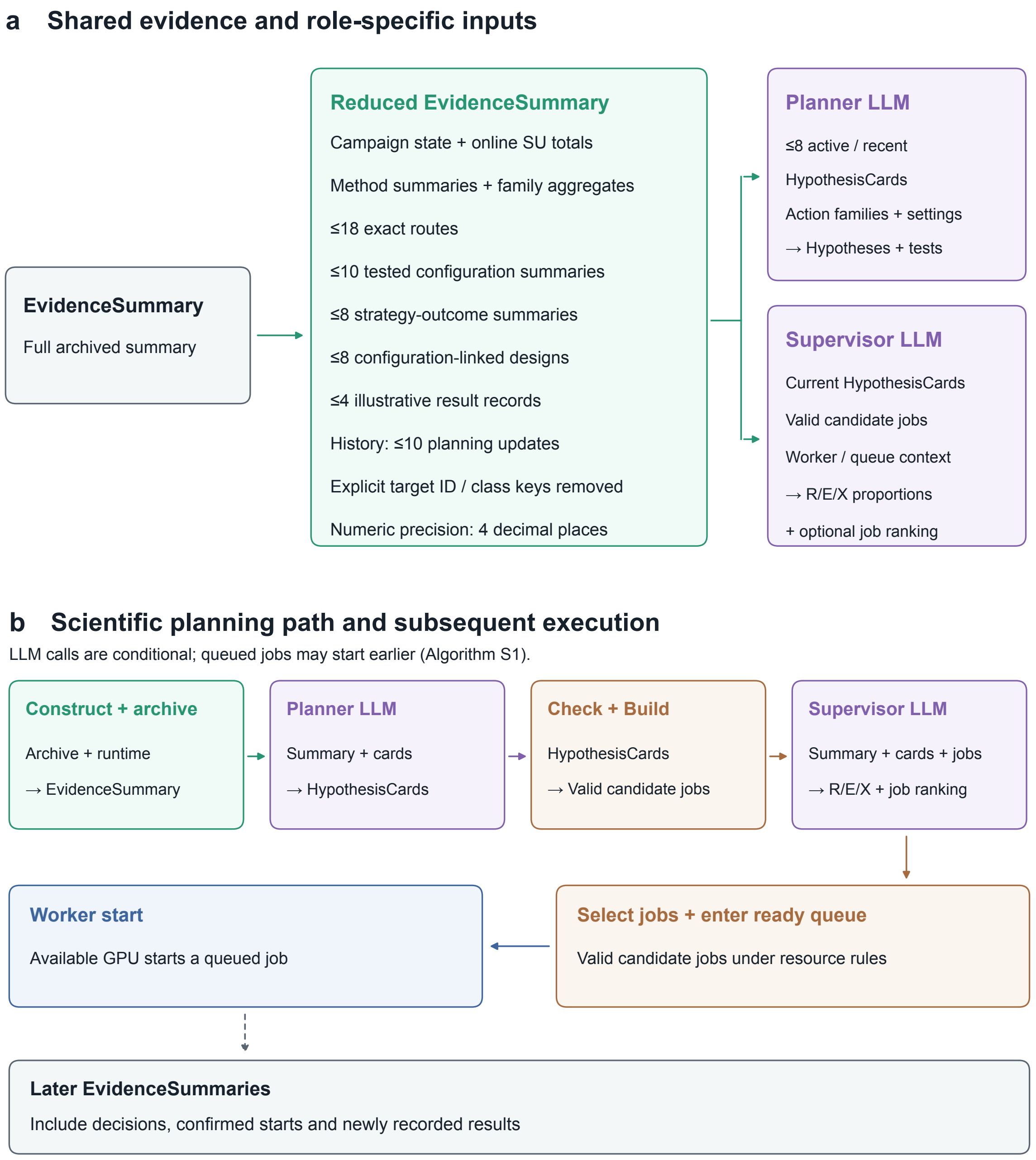

Figure S2 | **LLM inputs and the order of planning and execution.** **a**, The reduced *EvidenceSummary*, including campaign state, is shared by both LLMs (limits: Section C.5.2; route and collection definitions: Sections C.2.2 and C.4.1). The Planner additionally receives up to eight active or recent HypothesisCards, available action families, permitted settings and workload limits; the Supervisor receives current HypothesisCards, valid candidate jobs and selection context. Explicit target-identifier and target-class keys are removed from the evidence view. **b**, Purple denotes LLM proposals and prioritization, brown deterministic candidate construction and selection, and blue worker execution. Each EvidenceSummary is archived before conditional LLM calls (Algorithm S1). Queued jobs may start earlier; queue entry does not guarantee an immediate start. Later summaries include decisions, confirmed starts and newly recorded results. Read-only evidence refreshes before a job starts append no EvidenceSummary and call neither LLM.

bioRxiv preprint doi: https://doi.org/10.64898/2026.09.22.753604; this version posted September 24, 2026. The copyright holder for this preprint (which was not certified by peer review) is the author/funder, who has granted bioRxiv a license to display the preprint in perpetuity. It is made available under a CC-BY 4.0 International license.

C.5.2. Input contents and requested outputs

LLM input contents and size limits. An archived *EvidenceSummary* retained up to 32 exact-route rows and 20 planning-update summaries. A second reduction supplied both LLMs with cumulative online TM0.6 SU totals, all method summaries and family-level route rows, and at most 18 prioritized exact routes, ten tested configuration summaries (`recipes`), eight strategy-outcome summaries (`strategy_feedback`), eight configuration-linked designs (`exemplars`), four illustrative result records (`examples`) and ten update-history entries (Figure S2a; definitions in Section C.4.1). The Planner also received up to eight active or recent HypothesisCards, available action families, permitted settings and per-job workload limits. The Supervisor received current cards, valid candidate jobs and selection context, including available worker slots and recent starts.

Exact-route rows preceded family summaries. The reduction replaced the full parent-identifier list with a count and availability flag; concrete identifiers remained available in selected examples and for candidate construction. It removed explicit target-identifier and target-class keys recursively, omitted top-level missing scalars and empty strings, and retained empty evidence collections such as `recipes` and `joint_patterns` and method summaries with zero attempts. These omissions did not impute measurements.

Both dynamic prompts began with a short evidence digest followed by role-specific JSON. Section D.2.1 shows an archived EvidenceSummary excerpt and a reconstructed reduced-input excerpt, rather than a saved complete prompt. Neither role read arbitrary archive files or wrote measurements through this interface. Figure S2b places input preparation, calls and subsequent records within the planning update.

Requested outputs. Both roles requested one JSON object without server-enforced JSON formatting. Planner fields were `abstain`, `confidence`, `rationale` and `cards`, with optional `fail_reason`. A non-abstaining Planner response was requested to contain one to four cards, each citing evidence and specifying a claim, R/E/X affinities (relative weights used as priority hints), predicted changes in one or more qualification measurements, preservation constraints, proposed families and optional settings. Predicted changes shared the same ordered baseline references; Section C.9 defines their later evaluation. Section D.2.2 provides an archived card and its constructed candidate job.

The Supervisor returned a `mode_mixture` and a ranked `candidate_decisions` list, with abstention, confidence and rationale fields. Each returned candidate belonged to one priority, with unique global ranks from 1 to the number returned and unique positive ranks within each priority. Allocation values were finite, nonnegative and summed to one. The JSON below illustrates the requested fields for one candidate; bracketed strings are placeholders and the numerical values are illustrative. Section D.2.2 gives an actual prioritization example.

bioRxiv preprint doi: https://doi.org/10.64898/2026.09.22.753604; this version posted September 24, 2026. The copyright holder for this preprint (which was not certified by peer review) is the author/funder, who has granted bioRxiv a license to display the preprint in perpetuity. It is made available under a CC-BY 4.0 International license.

Illustrative Supervisor response (JSON).

```
{
  "abstain": false, "confidence": 0.6,
  "rationale": "<explanation of the proposed allocation>",
  "mode_mixture": {"rescue": 0.25, "explore": 0.50, "exploit": 0.25},
  "candidate_decisions": [{
    "candidate_id": "example_candidate", "mode": "explore",
    "global_rank": 1, "rank_in_mode": 1, "resource_class": "standard",
    "what": "<proposed computation>",
    "why": "<reason for its priority>",
    "evidence_refs": ["method_health"],
    "expected_signal": "<measurable outcome>",
    "stop_or_downgrade_if": "<condition for reducing priority>",
    "reasoning_trace": {
      "observed_signal": "<cited observation>",
      "inference": "<interpretation of that observation>",
      "action_implication": "<connection to the proposed computation>"
    }
  }]
}
```

Each card and decision requested three short explanation fields in `reasoning_trace`: `observed_signal`, `inference` and `action_implication`. These were reported explanations, not records of private model reasoning. A Supervisor response could rank only a subset of candidates, including none. Its resource classes supported auditing and tie-breaking; allocation bounds and execution remained deterministic (Sections C.7.1 and C.8.2).

Parsing and validation. Parsing extracted the first JSON object and normalized recognized field variants. Planner validation checked field types, evidence references, measurement directions and priority affinities; unavailable families were removed and candidate construction enforced setting bounds. Supervisor validation checked candidate/evidence references, allocation values, priority assignments, ranks and resource classes. Missing Supervisor explanation fields could be filled from returned references and explanation text. Planner repairs were deterministic. Only Supervisor errors involving duplicated candidates across priorities, invalid global ranks or unknown candidate identifiers permitted one corrective LLM call. Limited deterministic repair could then remove unknown candidate rows. Repaired outputs were revalidated; unusable responses followed Section C.7.1.

C.5.3. Prompt instructions

Each role receives a system prompt defining its task and a dynamic user prompt containing the current evidence and role-specific inputs (Figure S2a). The boxes below summarize the retained campaign implementation: system instructions are condensed, input bodies are schematic, and the corrective message is a template excerpt. They are not complete historical prompts, which were not archived. In the input schematics, bracketed strings stand for structured records or text supplied at runtime. Section C.5.2 specifies the requested output fields; Section D.2.2 provides archived responses and linked candidate jobs.

bioRxiv preprint doi: https://doi.org/10.64898/2026.09.22.753604; this version posted September 24, 2026. The copyright holder for this preprint (which was not certified by peer review) is the author/funder, who has granted bioRxiv a license to display the preprint in perpetuity. It is made available under a CC-BY 4.0 International license.

Shared scientific instructions (condensed)

Base decisions on supplied outcomes and constraints, without assumptions about target identity, target class or which method should perform best. Maximize new TM0.6 SU per elapsed worker-slot hour. For method and configuration comparisons, use recent exact-route outcomes and complete route costs before family summaries; use longer windows to account for delayed feedback.

Qualified duplicates add no new SU. Pending evaluation, unstarted jobs and missing measurements are unresolved or unavailable evidence, rather than measured failures. Auxiliary measurements can suggest follow-up tests but do not establish qualification or SU; keep them specific to their measurement source and do not average differently calibrated measurements into one reward.

Cite supplied evidence identifiers, use only available action families and permitted settings, and return one JSON object. Explain the observed signal, its interpretation and the proposed follow-up in three short `reasoning_trace` fields. These are reported explanations, not private model reasoning.

The campaign-level prompt objective used elapsed worker-slot time, including idle slot time; route comparisons used recorded full-route costs (Table S6). Before interpreting absence of a new SU as poor performance, the prompts suggested roughly 0.5 worker GPU-hours for low-cost, directly scored Complexa routes and 1.5–2 for delayed or higher-cost routes, including BindCraft, BoltzGen, ProteinMPNN redesign and Complexa MCTS. These were cost and feedback-delay guidance, not measured runtimes or hard timeouts.

Planner system instructions (condensed)

Propose one to four testable HypothesisCards from the latest evidence. Each card states a claim, cites evidence, proposes action families and settings, predicts qualification-measurement changes and specifies measurements to preserve. R/E/X affinities describe the purpose of the follow-up test; they do not assign a fixed method to each priority.

Predict changes only in pLDDT, iPAE or binder scRMSD: pLDDT increases, whereas iPAE and binder scRMSD decrease. Use the same ordered baseline references for all predicted changes in a card. Parent-bound jobs need concrete result identifiers; family summaries cannot substitute for parent designs.

Match the test to the observed weakness: sequence redesign or sequence hallucination for binder scRMSD; interface or backbone search for iPAE; and regeneration or pLDDT-directed search or refinement for low pLDDT with a plausible interface. Use past test outcomes and negative evidence to avoid repeating an unchanged, unproductive configuration. Preserve the other qualification measurements and obey registered setting bounds and workload limits.

The Planner's L0–L4 ordering was a preference, not a required sequence: L0, obtain initial coverage of generator roots (Section C.2.2); L1, change search or sampling settings within a family; L2, address a near-miss design or an unproductive parent–child line through parent-bound redesign or a justified same-family alternative; L3, adjust scalar reward weights with criterion-level evidence; L4, expand higher-cost search when recent productivity or repeated near misses justify it. L1/L2 changes tested a different scientific hypothesis rather than only increasing workload or repeating reward adjustments. The system prompt also explains action-family roles and diagnostic-to-setting relationships; the dynamic input supplies current availability and bounds.

bioRxiv preprint doi: https://doi.org/10.64898/2026.09.22.753604; this version posted September 24, 2026. The copyright holder for this preprint (which was not certified by peer review) is the author/funder, who has granted bioRxiv a license to display the preprint in perpetuity. It is made available under a CC-BY 4.0 International license.

Planner dynamic user prompt (schematic).

```
EVIDENCE DIGEST: <recent progress, costs and unresolved work>
HYPOTHESIS OUTCOMES: <status and results of retained cards>
{
  "evidence": "<reduced EvidenceSummary, including campaign state>",
  "active_hypotheses": "<up to eight active or recent HypothesisCards>",
  "seed_action_families": "<bootstrap family list, or an empty list>",
  "available_families_this_cluster": "<available action families>",
  "family_role_table": "<roles, parent needs and scoring status>",
  "allowed_params_per_family": "<permitted settings and domains>",
  "eval_budget_per_family": "<per-job workload limits>",
  "instructions": "<evidence-linked hypotheses and measurable tests>"
}
```

When provided, bootstrap families guide initial coverage. Prior deterministic advisory flags can be appended as `recent_critic_flags_last_tick`; despite the field name, these are not an additional Critic LLM call (Section C.5.4). Their presence depends on the call context. The prompt is an input for proposals, not permission to modify the archive or execute jobs.

Supervisor system instructions (condensed)

Use the current evidence, HypothesisCards and valid candidate jobs to propose R/E/X allocation proportions and optionally rank candidate jobs. Allocation values must be finite, nonnegative and sum to one; they concern future generation and redesign starts, not GPU-time fractions. Rank only supplied candidate identifiers. Each returned candidate belongs to one priority and has a unique global rank from 1 to the number returned; rank 1 expresses the immediate next-worker preference across priorities.

Prefer recent new SU per full exact-route cost. Among routes with reliable, approximately comparable recent efficiency, prefer the higher lower-quartile qualification-margin score, then its median (Section C.4.3). Treat sparse counts cautiously; quality does not compensate for a substantial efficiency disadvantage. Inspect source-specific diagnostics to select an evidence-supported follow-up, without counting native generator acceptance as qualification.

Keep proposed proportions consistent with immediate job priorities. Use campaign state, structural duplication, time without new SU, pending work and confirmed starts to interpret current needs. A job selected but not started is pending execution, not a failed scientific test. Required AF2 evaluation follows deterministic rules and is outside the LLM's R/E/X allocation.

Supervisor dynamic user prompt (schematic).

```
EVIDENCE DIGEST: <the same reduced evidence used by the Planner>
MIXTURE GUIDANCE: <state-dependent allocation guidance>
{
  "evidence": "<reduced EvidenceSummary, including campaign state>",
  "hypotheses": "<current HypothesisCards>",
  "candidates": "<valid candidate jobs with matched route evidence>",
  "selector_context": "<worker slots, pending jobs and recent starts>",
  "mode_mixture_ranges": "<state-dependent advisory ranges>",
  "instructions": "<allocation proportions and optional job ranking>"
}
```

bioRxiv preprint doi: https://doi.org/10.64898/2026.09.22.753604; this version posted September 24, 2026. The copyright holder for this preprint (which was not certified by peer review) is the author/funder, who has granted bioRxiv a license to display the preprint in perpetuity. It is made available under a CC-BY 4.0 International license.

Conditional Supervisor corrective message (template excerpt).

```
Your previous output rejected: <validation_error>.
Re-emit the JSON. Ensure each candidate_id appears in exactly ONE
mode and give every candidate_decision one unique contiguous
global_rank in 1..N, where 1 is the immediate next-worker choice.
Use only these candidate_id values: <supplied candidate_id list>.
```

This corrective message can be sent once for the specific ranking/reference errors described under Parsing and validation in Section C.5.2. It follows the original user prompt and rejected response, with the same Supervisor system prompt. Other unusable responses follow deterministic repair or fallback; the primary campaign controller uses the Planner and Supervisor LLM roles, rather than a separate Critic LLM.

Prompt guidance and execution rules. Quality and cost preferences are specified in Section C.7, using the scores in Section C.4.3. Campaign-state labels inform proposals, while candidate eligibility, allocation bounds and worker starts follow the rules in Sections C.4.5, C.6, C.7.1 and C.8. Prompts impose no fixed generator hierarchy and do not guarantee that a proposed job starts. Archived proposals and decisions record the responses; prompt instructions alone do not establish compliance.

C.5.4. Call conditions and advisory checks

The Planner's eight-card memory used the latest record for each card, prioritizing active cards and then recent supported or refuted cards; retired cards were excluded. Invalid, empty or abstaining output supplied no new cards, so construction used retained cards and candidates introduced by fixed rules. Refuted or retired cards could not generate candidates. Section C.9 defines card status and lifetime. The Supervisor was called with a non-empty card list and valid candidate pool; otherwise the controller recorded why it skipped the call. Invalid or abstaining responses used fallback (Section C.7.1).

Deterministic advisory check. When enabled, the check ran on valid, non-abstaining Planner output containing cards, before fallback to retained cards. It flagged proposed families whose prespecified runtime estimate exceeded the remaining wall time. It also flagged a configuration matching a retained failure recipe from the same family on at least two shared settings, including one beyond workload controls. All shared values had to match, the recipe needed at least two failed descendants, and its hash could not also appear among retained qualified recipes.

The check left current cards, candidates, selection and starts unchanged. Flags were stored as a zero-token, zero-latency `LLMCallRecord` and could inform a later Planner call; a subsequent clean record cleared earlier flags. Table S15, part A, reports these deterministic checks separately from model calls.

C.6. Candidate construction and validation

C.6.1. Validation of proposed jobs

Check + Build converts HypothesisCards into candidate jobs by resolving action families, proposed settings and required parents against the action registry and archive. Validation retains permitted settings, removes unsupported entries, clips numeric values to registered bounds and reduces

bioRxiv preprint doi: https://doi.org/10.64898/2026.09.22.753604; this version posted September 24, 2026. The copyright holder for this preprint (which was not certified by peer review) is the author/funder, who has granted bioRxiv a license to display the preprint in perpetuity. It is made available under a CC-BY 4.0 International license.

oversized workloads when repair is possible (Table S5). Unrepairable jobs remain ineligible. Parent-bound redesign and evaluation require a resolvable structure with valid target and binder chains. Deterministic rules can then add or replace settings as described below; executor defaults fill only fields left unspecified. Section C.8 describes the subsequent queue and start-time checks.

C.6.2. Complexa configuration additions and replacements

Measurement-directed defaults and refinement. The *primary measurement* is the measurement named by the first predicted change in the HypothesisCard (Section C.5.2); it defaults to iPAE if no change is listed. A card with predicted changes but no suggested settings receives sequence hallucination for primary binder scRMSD, or adaptive sampling noise for primary iPAE (`sc_scale_noise=0.30` if no adaptive value is available). A proposed configuration emptied by validation does not receive this default. Greedy optimization enabled, a positive greedy-iteration count or a specified `greedy_percentage` selects `refinement_algorithm=sequence_hallucination`. For nonempty configurations without reward weights, primary iPAE or binder scRMSD also selects this refinement in stalled, deep-stall, near-miss-enriched, productive-with-duplication or structural-duplicate-collapse states, or when trusted near misses are present. State and clustering-reliability rules are defined in Sections C.4.5 and C.4.2.

Reward-weight changes. For reward checks, a *substantive search change* is a specified sampling-noise, steering-temperature, beam-width/branch, sample-count, replica-count, MCTS-search or sequence-refinement control. Values equal to explicitly registered workload defaults do not count; diffusion steps, batch size, filter limit or reward weights alone are insufficient. The builder uses the largest observation count in the primary measurement's group, across qualification and auxiliary summaries. The groups are fold confidence for pLDDT (qualification pLDDT, auxiliary binder pLDDT and design pTM), interface confidence for iPAE (iPAE, minimum iPAE, mean/maximum ipSAE, ipTM and design-to-target ipTM), and geometry for binder scRMSD (scRMSD, shape complementarity, contact density, hotspot RMSD and buried surface area). Other primary measurements use their own counts.

Below 32 observations, reward weights are removed unless the proposal is Complexa MCTS with a substantive search change already present. If removal leaves no such change, the ordered additions below are attempted. With at least 32 observations, reward weights remain, but a proposal lacking a substantive search change receives one when available. Section D.2.2 shows a proposed weight removed by this check. Separately, Complexa best-of-n configurations with more than four replicas have batch size reduced to eight if it exceeds eight, retaining the requested sample and replica counts to limit memory use.

Adaptive sampling noise. Numeric additions use a fraction f of the registered range $[L, U]$, giving $L + (U - L)f$. Values are rounded to the nearest integer, with ties to even, when both bounds are integral and $U - L > 1$; otherwise to four decimal places. The resulting configuration is checked against the same domains and workload limits. For `sc_scale_noise`, g denotes fold confidence, interface confidence or geometry, using the measurements listed under Reward-weight changes. Within each group, F_g is the largest fraction of observations labeled fail for any measurement; S_g is the largest fraction labeled pass or near-pass. Each fraction uses that measurement's observation count as its denominator. The two maxima can come from different measurements and need not sum to one. Qualification summaries take precedence over auxiliary summaries for the same measurement; missing or zero-count summaries contribute zero.

bioRxiv preprint doi: https://doi.org/10.64898/2026.09.22.753604; this version posted September 24, 2026. The copyright holder for this preprint (which was not certified by peer review) is the author/funder, who has granted bioRxiv a license to display the preprint in perpetuity. It is made available under a CC-BY 4.0 International license.

Let r_{MPNN} be the largest auxiliary diagnostic-improvement score among ProteinMPNN redesign rows in `route_values`, or zero if absent (Section C.4.3). These substituted zeros disable evidence contributions rather than recording failures. If any F_g, S_g or r_{MPNN} is positive, the first matching row sets the initial fraction:

$$f_0 = \begin{cases} 0.85, & F_{\text{fold}} \geq 0.60 \text{ or } F_{\text{geometry}} \geq 0.70, \\ 0.65, & F_{\text{interface}} \geq 0.70 \text{ and } \max(S_{\text{fold}}, S_{\text{geometry}}) \geq 0.50, \\ 0.60, & F_{\text{interface}} \geq 0.60 \text{ or } F_{\text{geometry}} \geq 0.50, \\ 0.75, & \text{the primary measurement is pLDDT}, \\ 0.50, & \text{otherwise.} \end{cases}$$

The fraction is capped at 0.50 when $r_{\text{MPNN}} \geq 0.50$, or at 0.60 when $0.35 \leq r_{\text{MPNN}} < 0.50$. If $\max_g S_g \geq 0.70$ and $\max_g F_g < 0.50$, it is additionally capped at 0.45. Without a positive measurement or redesign signal, the fraction is 0.55, increased to 0.65 only in deep stall or after at least 12 worker GPU-hours without a new SU, using the stall-time signal in Section C.4.2.

Ordered search-setting additions. When the reward check requires a search change, the following rules add permitted settings that are still unspecified, without overwriting accepted values. Duplicate pressure means a productive-with-duplication or structural-duplicate-collapse state (Section C.4.5). In steps 3 and 4, a beam-width addition also attempts to add `n_branch` at fraction 0.33; step 5 changes beam width alone.

1. For Complexa MCTS, add `n_simulations`, `exploration_prob` and `exploration_constant` at fractions 0.20, 0.57 and 0.33, respectively, then stop.
2. For FK steering, add temperature at fraction 0.45 under duplicate pressure and 0.30 otherwise; for primary iPAE or binder scRMSD, also add the adaptive noise above, then stop.
3. For the remaining Complexa families under duplicate pressure, first try beam width at fraction 0.43, then replicas at 0.60; stop after the first successful addition.
4. For primary iPAE, try beam width at fraction 0.43, then adaptive noise. For primary binder scRMSD, try sequence hallucination and adaptive noise together. For primary pLDDT, try beam width at fraction 0.35, then sampling noise at fraction 0.35. Stop if the applicable branch adds a setting.
5. If none applies successfully, try beam width at fraction 0.35, replicas at 0.50 and sampling noise at 0.45 in that order, stopping after the first addition.

C.6.3. Additional candidates generated by fixed rules

Prespecified MCTS probe. The builder can supplement card-derived jobs with the candidates below. These additions remain subject to validation and selection; availability does not force a launch. Outside the low-evidence state, it can add a Complexa MCTS configuration adapted from Proteina-Complexa's inference-time search setup for difficult targets (Didi et al., 2026b). The rule requires at least six worker GPU-h without a new SU and either (i) a design that fails pLDDT but passes iPAE, or (ii) failure of at least half of standardized pLDDT or auxiliary binder-pLDDT observations, together with a pass-or-near-pass rate of at least 25% for standardized iPAE or auxiliary minimum iPAE, ipTM or mean ipSAE. It uses the stall-time signal in Section C.4.2.

The probe uses 400 diffusion steps, four samples, batch size 16, 20 MCTS simulations, exploration probability 0.5, exploration constant 1.0 and filter limit 100. Its iPAE and pLDDT reward weights are -1.0 and 1.0; `sequence_hallucination` uses greedy optimization, 15 iterations and `greedy_`

bioRxiv preprint doi: https://doi.org/10.64898/2026.09.22.753604; this version posted September 24, 2026. The copyright holder for this preprint (which was not certified by peer review) is the author/funder, who has granted bioRxiv a license to display the preprint in perpetuity. It is made available under a CC-BY 4.0 International license.

`percentage=5`. The same configuration and trigger apply across targets. Additions stop after at least 3 route GPU-h and two ResultRecords with `ok` or `no_artifacts` status if the MCTS summary has no SU, no recent near miss, auxiliary diagnostic-improvement score below 0.35 and no pending standardized AF2 evaluation. This check uses the family summary, or the highest-cost exact route if that summary is absent.

Previously evaluated configurations. If no valid candidate offers parent-bound ProteinMPNN redesign or AF2 refolding with changed model, recycle or initialization settings, the builder can reintroduce up to three archived generation configurations. It uses exact-route rows, excluding untried, evaluation-only and collapse-risk routes and configurations already represented. Routes with SU or near-miss evidence precede those with only auxiliary progress. Their SU/GPU-h rate is the first available rate from the 3-GPU-hour, 6-GPU-hour or eligible 60-record window, followed by the lifetime rate. Without recent SU or near misses, a route needs at least 0.10 SU/GPU-h and is excluded after at least 1 route GPU-hour in any recent window. After 12 worker GPU-hours without a new SU, it is also excluded if only lifetime evidence remains, or its rate comes from the 60-record window without a rate or positive SU count from either cost-based window. Window and route definitions are in Sections C.4.1 and C.4.4.

A route lacking SU and near-miss evidence can instead be reintroduced when its outputs require separate AF2 evaluation, none remains pending, its auxiliary diagnostic-improvement score is at least 0.35 and accumulated route cost is at most 6 GPU-hours. Its recent-productivity status must not be `dry_low_quality`, `dry_duplicate` or `collapse_risk`. General-status `defer` routes are eligible only through this auxiliary-score rule. For both paths, 60-record rates are used only when the route has neither recorded downstream AF2-evaluation cost nor an AF2 qualification role.

Candidates from other generator roots. The *dominant root* is the generator root (Section C.2.2) with the most accumulated generator-route GPU time, using exact-route rows when available and family summaries otherwise; the lexicographically larger root name breaks a tie. For this candidate-availability rule, current SU evidence means a positive SU count or rate in any route or family summary for that root in the 3- or 6-GPU-hour window. A 60-record count or rate also qualifies before 12 worker GPU-hours without a new SU, but only for routes without downstream AF2-evaluation cost or an AF2 qualification role. These windows and costs follow Sections C.4.1 and C.4.2.

When feasible, at least two other roots are represented among valid candidate jobs and queued or running jobs if any of the following holds:

1. The state is low evidence or stalled, cumulative and recent SU counts are zero, and at least 0.10 recorded worker GPU-h and one ResultRecord have accumulated; a dominant root exists and lacks current SU evidence.
2. The state is deep stall and the dominant root lacks current SU evidence.
3. The state is stalled or structural duplicate collapse, at least six worker GPU-h have passed without a new SU, and the dominant root lacks current SU evidence.

In either productive state, the smaller requirement is one other root when recent SU gain is positive, at least four of the preceding ten planning updates were deep-stall updates with zero recent SU gain, and cumulative throughput is at most 0.25 SU per worker GPU-h. Recent gain uses the 60-record window (Section C.4.2). An additional root must have an untried or under-tested family, a family awaiting AF2 evaluation, or a family with SU, recent near misses, positive SU/GPU-h or auxiliary

bioRxiv preprint doi: https://doi.org/10.64898/2026.09.22.753604; this version posted September 24, 2026. The copyright holder for this preprint (which was not certified by peer review) is the author/funder, who has granted bioRxiv a license to display the preprint in perpetuity. It is made available under a CC-BY 4.0 International license.

diagnostic-improvement score at least 0.35. The time-without-new-SU conditions use the stall-time signal, whereas cumulative throughput uses recorded worker cost (Table S6).

C.7. Supervisor prioritization and deterministic selection

Supervisor ranking instructions. The Supervisor ranks candidate jobs across **Rescue**, **Explore** and **eXploit** and proposes their desired allocation proportions. It is instructed to compare exact routes by recent SU/GPU-h, including required evaluation costs. When the lower of two rates is at least 85% of the higher, it should prefer the higher 25th percentile, then median, of the online quality score q (Section C.4.3). The prompt asks for caution with few observations or little compute; it imposes no additional minimum sample count for this comparison. Outside that rate range, quality does not override efficiency. A lower-cost route should precede a more expensive route when it yields comparable or greater SU/GPU-h with adequate quality.

The prompts also request more diversification after six worker GPU-hours without new SU and discourage treating near misses as sufficient progress after twelve. Following limited recovery from sustained deep stall, they request continued consideration of another generator root. These are instructions to the LLM; Sections C.6.3 and C.8.2 specify the separate candidate-availability and concurrency rules. Section D.2.2 shows an archived prioritization example.

C.7.1. Response handling and allocation bounds

Response handling. Table S8B distinguishes a usable Supervisor global order, valid allocation proportions without a global order, and fallback. A propagated fallback reason replaces the Supervisor proportions with the state-dependent values in part A and disables its global order. Retained cards can still supply candidates. If the response retains individual candidate decisions, their within-priority ranks and resource classes can still enter the fixed ordering described below. Candidate-specific failures affect that candidate alone. Confidence recording follows Section C.5.1.

Allocation bounds. Desired proportions are normalized and adjusted using either the basic or conditional bounds in Table S8C. Conditional bounds apply on fallback, in stalled or structural-duplicate-collapse states, when structural concentration is at least 0.50, when reported Supervisor confidence is below 0.65, or when at least 30% of the latest four available LLM-call records indicate fallback or non-OK parsing. Missing confidence and deep stall alone do not activate them. These bounds constrain desired shares of subsequent confirmed starts; global ordering and candidate availability can change realized start shares and GPU-time fractions.

Structural concentration is the larger available dominant-cluster fraction in the latest 60 ResultRecords: among all records with an online structural assignment, or among qualified records with a qualified-cluster assignment. The qualified fraction is used only with at least four assigned qualified records; these are records, not four distinct SU. Under conditional bounds, concentration ≥ 0.50 in either productive state sets the **eXploit** ceiling to 0.60 and the **Explore** interval to 0.20–0.40, retaining other bounds. In the near-miss-enriched state, concentration ≥ 0.70 raises the **Explore** ceiling to 0.50 while retaining the **Rescue** floor. These thresholds differ from the 0.75 stalled-state test in Section C.4.5.

bioRxiv preprint doi: https://doi.org/10.64898/2026.09.22.753604; this version posted September 24, 2026. The copyright holder for this preprint (which was not certified by peer review) is the author/funder, who has granted bioRxiv a license to display the preprint in perpetuity. It is made available under a CC-BY 4.0 International license.

Table S8 | **Fallback and allocation rules.** Part A gives the initial desired R/E/X shares when selection takes the fallback path; Part B resolves the selection policy from Planner and Supervisor outcomes; Part C gives the basic and conditional bounds. The normalized shares satisfy $R + E + X = 1$. Shares refer to confirmed starts under R/E/X control (Section C.2.2), not success probabilities or guaranteed start-time or GPU-time fractions.

Fallback and allocation rules

A. Prespecified fallback allocation proportions

Campaign state	**Rescue**	**Explore**	**eXploit**
Low evidence	0.30	0.35	0.35
Productive	0.25	0.10	0.65
Productive with duplication	0.35	0.10	0.55
Structural duplicate collapse	0.15	0.75	0.10
Near-miss-enriched	0.50	0.20	0.30
Stalled	0.35	0.45	0.20
Deep stall	0.30	0.50	0.20

B. Failure handling and selection-policy resolution

Resolved path	Selection behavior
Valid Supervisor global ranks; no propagated fallback reason	Use its allocation proportions and current global order. A shorter order ranks only the returned subset.
Valid proportions without a usable global order; no fallback reason	No usable global order; the adjusted proportions and carried allocation difference guide priority quotas.
Invalid or abstaining Supervisor	Use the state-dependent fallback proportions and quota selection; no global order is used.
Planner reason `planner_invalid`, `planner_abstain` or `planner_empty_cards`	Propagate these reasons even through a valid Supervisor response: use fallback proportions and disable global ordering. Retained within-priority ranks can still enter fixed pool ordering (Section C.7.2). Other Planner reason strings do not by themselves activate this propagation.
One candidate fails eligibility or a live start check	Reject or defer that candidate; other proposals retain their own checks. This event alone does not create global response fallback.
Low confidence with valid output	Retain the ordinary response path, audit confidence and apply the separate conditional-bound tests.

C. Bounds on the normalized desired allocation proportions

Campaign state	Basic bounds	Conditional bounds
Low evidence	$E \geq 0.05$	$E \geq 0.05$
Productive	$X \leq 0.95$, $E \geq 0.05$	$0.45 \leq X \leq 0.80$, $R \geq 0.10$, $0.05 \leq E \leq 0.20$
Productive with duplication	$X \leq 0.90$, $E \geq 0.05$	$0.40 \leq X \leq 0.70$, $R \geq 0.20$, $0.05 \leq E \leq 0.30$
Structural duplicate collapse	$X \leq 0.30$, $E \geq 0.45$	$X \leq 0.20$, $R \leq 0.30$, $E \geq 0.55$
Near-miss-enriched	$R \geq 0.10$	$R \geq 0.40$, $E \leq 0.30$
Stalled	$X \leq 0.35$, $R \geq 0.25$, $E \geq 0.25$	Same as basic bounds; conditional bounds always apply.
Deep stall	$X \leq 0.40$, $E \geq 0.35$	$X \leq 0.30$, $R \geq 0.15$, $E \geq 0.45$

Pending AF2 jobs and the Rescue-floor waiver. For an update with K_* additional queue positions, this check uses capacity $4\max(1, K_*)$. It counts distinct selected or started AF2 candidate jobs without a linked ResultRecord. Recorded dispatch or parsing failures remove candidates, except deferrals caused by the high-cost running-job limit. At a count/capacity ratio ≥ 0.95, the **Rescue** lower bound is waived. This count is separate from ready-queue length and the unevaluated-design count (Section C.8.1).

The controller clips out-of-range proportions to their bounds and redistributes the remaining

bioRxiv preprint doi: https://doi.org/10.64898/2026.09.22.753604; this version posted September 24, 2026. The copyright holder for this preprint (which was not certified by peer review) is the author/funder, who has granted bioRxiv a license to display the preprint in perpetuity. It is made available under a CC-BY 4.0 International license.

share proportionally among priorities not held at a bound; a zero remaining input weight gives equal shares. It repeats the correction for at most five passes. These adjustments change allocation targets without changing candidate validity. Queue capacity and worker availability remain separate quantities (Algorithm S1).

C.7.2. Candidate pools and ordering

Global order and remaining positions. A usable global order assigns unique positive ranks to the candidate identifiers returned by the current valid Supervisor response; it need not include every candidate. The selector considers that subset in global-rank order, then stable identifier order, subject to validation and high-cost limits. Family/route penalties and quotas do not reorder this subset. Remaining queue positions can be filled from other valid candidates using the fixed ordering below. Without a usable global order, allocation quotas determine how many positions each priority receives. Candidate priorities use Supervisor assignments when applicable and otherwise HypothesisCard affinities; candidates without an assignment can fill otherwise unused positions. Selection determines queue admission; ordering before worker starts follows Section C.8.2.

Family penalties. Family penalties place candidates later in quota selection and filling of remaining positions. They require qualified-structure clustering status `ok` with coverage ≥ 0.999; reports of no qualified designs or no structures also permit the check. Standardized AF2 evaluation is excluded. A positive recent family SU/GPU-h rate, direct or attributed through redesign/evaluation, removes the penalty; these 60-record method-summary rates are defined in Section C.4.1. Otherwise:

1. At least 3 recorded worker GPU-h with no direct or attributed SU and no trusted near miss gives a strong penalty.
2. At least 8 worker GPU-h gives a strong penalty when the best eligible comparison rate is at least 0.50 SU/GPU-h and the family's lifetime rate is below either 0.25 SU/GPU-h or 35% of that comparison rate. A trusted recent near miss reduces this to a weak penalty.
3. If neither stronger rule applies, at least 6 worker GPU-h without recent SU or a trusted recent near miss gives a weak penalty.

An eligible comparison family has at least two credited SU and effective cost $g \geq 2$ GPU-hours. Its unadjusted rate is the largest direct or attributed, recent or lifetime SU/GPU-h rate; g is its largest reported direct or attributed SU count divided by that rate. Multiplying the rate by $g/(g+2)$ reduces the influence of limited observations; the highest adjusted rate is the comparator above. Near-miss reliability follows Section C.4.2.

Route penalties and deferral. An exact route receives no penalty when its recent-productivity status is productive, productive with duplication or either delayed-productive variant. Otherwise, general status `diversify` gives a weak penalty; `defer`, `collapse_risk`, or recent-productivity status `dry`, `dry_duplicate` or `dry_low_quality` gives a strong penalty (Section C.4.4). On the quota path, a strong family penalty moves its candidates out of the initial pool if another candidate remains eligible for that pool. Strong route penalties then defer candidates when an alternative in the same priority remains. Under conditional allocation bounds, **Explore** candidates remain in the initial pool in both checks. Unfilled positions are considered first for remaining candidates, then family-deferred candidates, then route-deferred candidates, subject to the evaluation-backlog restriction below.

bioRxiv preprint doi: https://doi.org/10.64898/2026.09.22.753604; this version posted September 24, 2026. The copyright holder for this preprint (which was not certified by peer review) is the author/funder, who has granted bioRxiv a license to display the preprint in perpetuity. It is made available under a CC-BY 4.0 International license.

Fixed ordering within the pools. The ordering first compares recent family overrepresentation, family penalty and route penalty, in that order. A family is overrepresented when it supplies at least 70% of the latest ten available confirmed generation/redesign starts and either the state is structural duplicate collapse or it is productive with duplication with $f_{\mathrm{dup}} \geq 0.75$ (Eq. (S3)). After these penalties, unranked candidates use descending exact-route SU/GPU-h before the within-priority rank comparison; candidates with a Supervisor rank use route rate only to break equal ranks. Resource class, estimated cost class and stable identifier resolve remaining ties; cost classes are ordered low, diagnostic, standard and extended.

The route rate is the first available rate from the 3-GPU-hour, 6-GPU-hour or eligible 60-record window, then the lifetime rate (Section C.4.1). The 60-record rate is eligible only without downstream AF2-evaluation cost or an AF2 qualification role. For an unranked candidate with absent or zero route rate, a trusted recent family rate can substitute. Quota selection and filling of remaining positions initially limit a family to two candidates, increased to K_* under basic bounds in productive or low-evidence states. This limit can relax to fill unused positions; the separate high-cost limits in Section C.8.2 still apply.

C.7.3. Allocation across starts and quota safeguards

Carried allocation difference. For adjusted proportions $\mathbf{p} = (p_R, p_E, p_X)$, the controller reconstructs a carried allocation difference $\mathbf{d}$ from archived decisions and starts. At each archived update, it adds that update's proportions multiplied by the larger of its approved generation/redesign launch-intent count and linked confirmed-start count, then subtracts one for each confirmed start from the corresponding priority. Each component is clipped to $[-3, 3]$ after each update. An approved or queued job that never starts therefore does not count as a realized allocation. Global-order updates contribute their proportions and starts to the same reconstruction, although quotas do not control their immediate selection.

For a quota update with K_* positions, temporary allocation differences are $d_m + K_* p_m$ for $m \in \{R, E, X\}$, clipped to $[-3, 3]$. Each position is assigned to an eligible priority with the largest difference; ties after rounding to six decimals favor the larger p_m, then **eXploit**, **Rescue**, **Explore**. Each assignment subtracts one from that temporary difference. These provisional subtractions allocate the current quotas; subsequent reconstruction uses confirmed starts. Priorities without eligible candidates receive no quota, and unused positions can be filled through Section C.7.2.

Maintaining productive computation. On the quota path, one **eXploit** position can be preserved in productive, productive-with-duplication or near-miss-enriched states when stall time is at most 6 worker GPU-h, prior **eXploit** allocation has not been exceeded by more than one start, and an eligible candidate has positive route or recent family SU/GPU-h. Reused configurations labeled `dry_duplicate` without a positive 3-GPU-hour rate or a 3-/6-GPU-hour SU gain cannot qualify on route rate alone. Structural duplicate collapse prevents this safeguard. Stall time follows Section C.4.2.

With at least three positions, stall time at most 3 worker GPU-h and either campaign recent throughput ≥ 0.50 SU/GPU-h or an SU gain of at least two, the quota can preserve two **eXploit** positions. The gain is the increase above the previous highest cumulative SU count, or the 60-record gain when that increase is zero or unavailable. Both leading **eXploit** candidates must be outside the high-cost family, have low, diagnostic or standard estimated cost, avoid the reused-configuration exclusion above and have route or recent family throughput ≥ 0.50 SU/GPU-h. Required AF2 evaluations cannot supply these positions.

bioRxiv preprint doi: https://doi.org/10.64898/2026.09.22.753604; this version posted September 24, 2026. The copyright holder for this preprint (which was not certified by peer review) is the author/funder, who has granted bioRxiv a license to display the preprint in perpetuity. It is made available under a CC-BY 4.0 International license.

Retaining a cross-generator candidate. After ordinary selection, the quota path can retain one candidate added by the cross-generator rule in Section C.6.3. It fills an unused position or replaces a candidate that lacks a Supervisor within-priority rank of one. Such a candidate from BindCraft, BoltzGen or ProteinMPNN is not restored from the route-deferred pool when its family has at least 32 source ResultRecords without a linked standardized AF2 result and at least four with one, with no family or matching-route SU or near miss and no promising pending structure. This restriction applies both to the cross-generator safeguard and to ordinary filling from that pool. Start-time validity and capacity checks still follow Section C.8.2.

C.8. Evaluation admission and worker execution

Generation, redesign and standardized AF2 evaluation share the three worker GPUs. Evaluation admission determines which designs enter the common qualification protocol; queue management determines when their jobs start. Figure S2b and Algorithm S1 show these separate steps. Designs awaiting evaluation remain pending, without being counted as qualification failures.

C.8.1. Standardized AF2 evaluation: admission and capacity

Eligible outputs and admission signals. Section B.1.1 specifies which outputs can receive standardized AF2 evaluation. The controller schedules these evaluations alongside generation and redesign, using native measurements and previous route outcomes to prioritize inputs. These signals affect admission and ordering; qualification and SU credit still require the common evaluation rules. An *evaluation-source family* is the generation or redesign family that produced an evaluation input. A *native-like* BindCraft or Complexa output has generator-native pLDDT ≥ 90 on the 0–100 scale, normalized iPAE $\leq 7/31$ and binder RMSD or scRMSD < 1.5 Å. A weaker *proxy-promising* signal is defined separately for each tool:

- **BindCraft:** a generator-accepted output, or an output with all three native measurements and at most one criterion outside the near-pass tolerances in Section C.4.1.
- **BoltzGen:** at least two of design ipTM ≥ 0.80, design-to-target ipTM ≥ 0.65, interface ipTM ≥ 0.65, minimum design-to-target PAE ≤ 7 Å, structure confidence ≥ 0.75 and native-refold RMSD < 2.0 Å.
- **ProteinMPNN:** global or local per-residue negative log-likelihood ≤ 1.20, with sequence recovery—the fraction of parent residues retained—either unreported or between 0.15 and 0.85, inclusive.

An input receives the highest evaluation priority if it is native-like or its exact route has status `promote`, a recent SU gain, a positive selected route rate, or a recent near miss together with status `healthy` (Section C.4.4). This input-priority test is broader than the route status `promote`. The rate follows the window preference in Section C.7.2, including lifetime fallback when recent rates are unavailable. Within each route, inputs satisfying this test precede proxy-promising inputs; the remaining inputs follow, with `diversify` and then `defer/collapse_risk` given lower priority. Native-score ranking and stable identifiers resolve ties. Eligible route groups are visited in turn, taking at most one input from each per pass.

Cumulative allowance for each route. Each exact upstream route initially receives an allowance of four standardized AF2 evaluations. The cumulative allowance can advance to 8, 16, 32 and 64, then double further as evidence improves. Advancing from four to eight permits at most four

bioRxiv preprint doi: https://doi.org/10.64898/2026.09.22.753604; this version posted September 24, 2026. The copyright holder for this preprint (which was not certified by peer review) is the author/funder, who has granted bioRxiv a license to display the preprint in perpetuity. It is made available under a CC-BY 4.0 International license.

additional evaluations, subject to the capacity rules below. Native or proxy signals affect which inputs are evaluated first but do not bypass this progression.

Advancement uses the latest archived route summary's count of returned standardized AF2 ResultRecords, including unsuccessful outcomes. At a completed boundary, the controller compares that summary with the most recent earlier summary whose count is no greater than the preceding configured boundary: zero for the four-to-eight decision, or at most four for eight-to-sixteen. At least one of six measures must increase: route-attributed SU, individual near-miss result count, qualified-cluster count, 25th-percentile or median online quality score, or auxiliary diagnostic-improvement score (Sections C.4.1 and C.4.3). Score increases must exceed 10^{-6}; the diagnostic score is stored to three decimal places. Near-miss evidence requires the reliability check in Section C.4.2. Missing comparison values contribute zero to this test.

Without an increased signal, the allowance stays at the completed boundary; raw qualified-design count does not advance it. A partly completed block can finish its current allowance: five returned evaluations permit continuation to eight, not sixteen. Confirmed starts and evaluations already selected for admission are subtracted from the authorized total, preventing repeated admission from repeatedly advancing the same evidence. Above 64, the allowance continues doubling, but the comparison lookup still uses the largest configured boundary below the current count. The spare-capacity exception below can admit one further evaluation without advancing this allowance.

Family-level admission limits. An input is *evidence-backed* for these limits if it satisfies the highest-priority test above or is proxy-promising. Ordinary admission allows at most 24 currently unsupported evaluations per evaluation-source family, counting started and already selected jobs. A family's total approvals under the same planning-update identifier are compared with a cap of four for an unsupported candidate or sixteen for an evidence-backed candidate; these are not separate additive quotas. Route allowances still apply to both groups.

Limits based on recent starts. Once at least 20 starts have been recorded, reservation compares the number of standardized AF2 starts among the latest 40 available starts with a limit of 24, reduced to 20 in productive-with-duplication or structural-duplicate-collapse states. Before 20 starts, the corresponding limits are 12 and 10. If this budget is exhausted, available native-like inputs permit an exception of at most two positions, or one in deep stall; otherwise an available input satisfying either the proxy-promising or highest-priority test permits one. These counts limit reservations, not the fraction of GPU time spent on evaluation.

Queue reservations. AF2 jobs can be inserted at the front of the queue before dispatch and again during queue refill. The passes use free workers $F(\mathcal{W})$ and queue space $K(\mathcal{Q})$, respectively; the first can temporarily extend the queue beyond its target depth. Both passes share a per-loop ceiling of one, increased to two when the pending-output count used for this ceiling reaches 32. That count uses native-like or proxy-promising outputs when any are pending, otherwise all eligible unevaluated outputs. Each pass subtracts both earlier reservations in that loop and currently running AF2 jobs from the ceiling (Algorithm S1). An AF2 job already in the queue prevents another reservation.

Each pass also checks its available positions and eligible candidate jobs. The local limit is at most half those positions rounded up and leaves one unreserved when two or more are available. Its one-to-two increase requires 32 eligible candidates in that pass, so each pass admits at most one with the three-worker setup. During deep stall, the shared ceiling is one when an eligible output still awaits AF2 evaluation; native-like evidence is not required. Excess queued AF2 reservations are

bioRxiv preprint doi: https://doi.org/10.64898/2026.09.22.753604; this version posted September 24, 2026. The copyright holder for this preprint (which was not certified by peer review) is the author/funder, who has granted bioRxiv a license to display the preprint in perpetuity. It is made available under a CC-BY 4.0 International license.

cancelled when the refreshed state enters deep stall. A single free position can be used for evaluation when an unevaluated output exists; otherwise that position is left for generation or redesign. These rules reserve queue positions rather than assigning a GPU permanently to evaluation.

Filling spare queue positions. After generation and redesign selection, one AF2 job can be appended if queue space remains, the state is not deep stall and no standardized AF2 job is queued or running. This path first uses ordinary route allowances. If they provide no candidate, it can select one from a route whose allowance is exhausted, bypassing the cumulative unsupported-family limit as well. The per-update family limits and start checks still apply. This exception uses spare queue capacity; it neither establishes improved evidence nor doubles the route allowance.

C.8.2. Queue admission, starts and result collection

Queue refill. Candidate ordering and allocation follow Sections C.7.2 and C.7.3, with validation in Section C.6. Queue space $K(\mathcal{Q})$ and free workers $F(\mathcal{W})$ are defined in Algorithm S1. Planning can refill the queue while all workers are occupied after the first target ResultRecord arrives; it pauses beforehand if every worker is busy. Queued jobs can start before new planning calls and again after queue refill, at the two dispatch points in the algorithm.

Queue order and new evidence. Before dispatch, required AF2 jobs come first. Generation and redesign jobs then follow their recorded annotations: recent SU or near-miss evidence, diagnostic improvement, ordinary proposals, and finally reused configurations supported only by lifetime evidence. Within a group, jobs with selection records precede those without them; newer selection updates come first, with existing queue order breaking ties. This ordering uses annotations in candidate and selection records rather than recomputing route scores. New results or a state change alone do not remove ordinary queued jobs. The exception is jobs selected in low evidence before any result existed: once the first result arrives, these queued jobs are removed so planning can use the new evidence.

BindCraft concurrency limit. BindCraft is the high-cost family, with an ordinary limit of one running job. When selection has more than one queue position to fill, at least one position is withheld from BindCraft; this bound applies even if no alternative is available. The three-worker pool likewise permits at most two simultaneous BindCraft jobs.

For this concurrency check, current route evidence means a positive SU count in the 3- or 6-GPU-hour window, a positive 3-GPU-hour rate or a trusted recent near miss. Rows with neither downstream AF2 cost nor an AF2 qualification role can also use the 60-record SU count and, when the 3-GPU-hour rate is missing, its rate (Section C.4.1). The limit can increase from one to two under any of these conditions:

1. **Family evidence:** a positive recent direct or attributed SU/GPU-h rate or a trusted recent near miss in the method summary (Section C.4.1); alternatively, lifetime SU or near-miss evidence with a lifetime rate ≥ 0.25 SU/GPU-h, provided that evidence has not become stale.
2. **Route evidence:** the family's highest-rate route or family summaries provide at least one SU, at least 1.0 route GPU-h and current SU or near-miss evidence. Their rate, taking the larger current or lifetime value, must be at least 0.10 SU/GPU-h and 80% of the best current rate among competing summaries with current evidence. For tied highest-rate summaries, the largest count and cost and the presence of current evidence are used.

bioRxiv preprint doi: https://doi.org/10.64898/2026.09.22.753604; this version posted September 24, 2026. The copyright holder for this preprint (which was not certified by peer review) is the author/funder, who has granted bioRxiv a license to display the preprint in perpetuity. It is made available under a CC-BY 4.0 International license.

3. **Testing an alternative during a stall:** at least 32 ResultRecords, at least 0.75 worker GPU-h without new SU, and a best recent non-evaluation-family rate below 0.25 SU/GPU-h, in low evidence, stalled, deep stall or structural duplicate collapse.
4. **Repeated prioritization with delayed starts:** at least six BindCraft entries in archived Supervisor candidate decisions, a start-to-entry ratio ≤ 0.35, and at least one capacity-deferred or selected-but-unstarted job. The state must be low evidence, stalled, deep stall or structural duplicate collapse, or the campaign must have fewer than four SUs. Lifetime evidence must not be stale.

Lifetime evidence is stale when neither family nor route summaries retain current SU or trusted near-miss evidence and a previously productive summary has accumulated at least 1.0 recent route GPU-h. Recent windows follow Section C.4.1; route rates follow Section C.7.2. SU-based promotion requires the clustering check described there, and near misses use Section C.4.2. Conditions 2–4 are blocked after at least 6 recorded family GPU-h and more than one timeout without direct or attributed SU or trusted lifetime/recent near misses. Raising the limit permits another start but does not select a job; queued jobs do not count as running jobs.

Starts, deferrals and failures. Approved LaunchDecisions permit queue admission; DispatchRecords record starts and execution events (Section C.3). Before a start, the controller resolves required inputs and rechecks current constraints. A concurrency deferral returns the job to the queue without consuming a retry. Other transient start failures are retried at most three times, for four attempts in total, after all currently free workers have been considered. Permanent failures and exhausted retries remove the job. DispatchRecords retain terminal start failures and distinct capacity-deferral reasons; intermediate transient retries need not each produce a record. A later runtime or output-collection failure does not erase an earlier confirmed start.

Incremental and final result collection. Running BindCraft jobs are checked for new outputs at the interval in Section C.8.3. Only previously unarchived ResultRecords are appended. Worker time since the preceding addition of results is divided among the new records; final parsing collects any remaining outputs. Thus a running job can already contribute recorded results and cost. Re-reading archived outputs does not append them again.

If final parsing yields no new design after earlier results, or no output at all, a synthetic completion record retains an uncharged remainder ≥ 0.005 GPU-hours or a failed outcome. It represents cost and outcome rather than another design. Successful re-reading of outputs already archived through another path adds no second charge. A successful, empty parse below that threshold instead records an output-collection failure without a ResultRecord. Final-parsing exceptions are recorded separately; after at least 0.005 elapsed GPU-hours, a failure record retains the full elapsed job cost, which can overlap earlier partial charges. Table S6 distinguishes these records from other compute-accounting quantities.

C.8.3. Worker time limits and failure handling

Polling and planning failures. Active controller iterations normally wait 5 s between polls; an iteration with no planned, queued, dispatched or running jobs waits 120 s. A cached campaign-state calculation can be reused for up to 60 s when result counts, worker availability, running-worker cost rounded down in 0.25-GPU-hour increments, and queued/running job counts by family are unchanged. A failed state check retains the previous state. A full planning-update exception preserves

bioRxiv preprint doi: https://doi.org/10.64898/2026.09.22.753604; this version posted September 24, 2026. The copyright holder for this preprint (which was not certified by peer review) is the author/funder, who has granted bioRxiv a license to display the preprint in perpetuity. It is made available under a CC-BY 4.0 International license.

already appended records, waits 60 s and restarts the loop before further admission or dispatch. Incremental BindCraft collection runs at a default 600-s interval, bounded below by 300 s.

Progress checkpoints. A separate checkpoint saves elapsed campaign time and monitoring evidence at a default 60-s interval, including when all workers are occupied. When native-like or proxy-promising designs await standardized AF2 evaluation and a standardized AF2 job is running, the checkpoint recomputes current evidence and can update the campaign state. Otherwise, it reuses the latest archived summary and updates elapsed time. Checkpoints neither append an EvidenceSummary to the JSONL history nor call an LLM.

Job timeouts. Limits are elapsed seconds on one worker slot. Complexa Beam, best-of-n and FK jobs have 5,400-s ceilings; MCTS has a 9,000-s ceiling. Standardized AF2, ProteinMPNN and BoltzGen ceilings are 2,160, 900 and 10,800 s. When a limit is exceeded, the controller attempts to collect available outputs and terminates the unfinished job. These ceilings limit overruns rather than specifying expected runtimes or selection costs.

BindCraft instead uses a progress watchdog. After a 5,400-s grace period, the first check starts a no-progress timer. An increase in scoreable output count resets it; more than 5,400 s without such progress triggers termination. Growth of intermediate files alone does not reset the timer.

Disabling repeatedly unsuccessful families. A family becomes unavailable after at least two timeout ResultRecords and 3 recorded worker GPU-h without qualifying or near-miss metric evidence in its attributed results, provided another available generation family not requiring a parent remains. Standardized AF2 is excluded. The controller prevents further candidates from that family and removes its queued jobs, while running jobs continue. Disablement itself records neither a timeout nor a new worker start.

Campaign time limit and shutdown. The 48-h controller limit is checked at loop entry and includes saved elapsed time if a campaign resumes. Planning and dispatch can finish within an iteration that has already begun. Section B.3 defines the separate reporting denominator of 144 H100 worker GPU-h.

At shutdown, busy workers are visited in pool order and each receives up to 600 s to finish. Normal completion is parsed before visiting the next worker; a timeout triggers attempted output recovery followed by termination. The scheduler can end the allocation before all waits finish. Final endpoint inclusion follows Section B.3 rather than these runtime waits.

C.9. Hypothesis feedback

Hypothesis feedback tests the measurement changes predicted in *HypothesisCards* (Section C.5.2). At the end of each planning update, deterministic rules evaluate previously active cards using results available at the start of that update. Updated cards are appended under the same hypothesis identifier for subsequent planning (Section C.3), without changing qualification or SU credit. These tests assess numerical predictions, not causal explanations. In the worked example in Section D.2.2, eight descendants qualified but none supported the card's specific predictions.

Results and comparison baselines. A result is linked to a card through the candidate job that produced it, including downstream evaluation jobs carrying the same hypothesis link. Each candidate

bioRxiv preprint doi: https://doi.org/10.64898/2026.09.22.753604; this version posted September 24, 2026. The copyright holder for this preprint (which was not certified by peer review) is the author/funder, who has granted bioRxiv a license to display the preprint in perpetuity. It is made available under a CC-BY 4.0 International license.

uses one comparison baseline shared by all predicted measurements: the first resolvable result among the card's ordered baseline references, followed by its cited evidence. Downstream evaluation can inherit this baseline; otherwise, a job without a recorded comparison baseline uses its execution parent. Thus the comparison baseline need not be the design supplied as input to the job. Results without a resolvable baseline are excluded. Counts are recomputed from all linked result–baseline pairs, rather than adding the same evidence again at each update.

Predicted improvement. Let d_{baseline} and d_{result} denote the measurement's nonnegative qualification deficits (Section C.4.1). For $d_{\text{baseline}} > 0.01$, the relative deficit reduction

$$(d_{\text{baseline}} - d_{\text{result}})/d_{\text{baseline}}$$

must meet the minimum specified in the card. When $d_{\text{baseline}} \leq 0.01$, support instead requires the specified absolute improvement in the favorable direction, defaulting to 1.0 in that measurement's units if the value is absent or zero. The 0.01 tolerance is applied in each measurement's units.

Result-level evidence. A result supports a card when every predicted change meets its improvement requirement and no checked limit on worsening measurements is violated. For each such limit, a baseline deficit ≤ 0.01 must remain ≤ 0.01; otherwise, the result deficit must not exceed the baseline deficit multiplied by one plus the card's permitted relative increase. A limit is checked only when both baseline and result measurements are available. Skipping a missing comparison does not establish that the measurement was preserved.

A result contradicts a card when every predicted measurement has a baseline deficit > 0.01 and a relative deficit reduction ≤ 0.05. Both support and contradiction require the predicted direction to match the measurement's favorable direction. Missing predicted measurements prevent either finding, with one exception: a missing baseline measurement can receive support when the linked standardized AF2 result passes the full qualification rule. Other predicted changes and preservation limits still apply. Support and contradiction are counted independently; results satisfying neither test are neutral.

Card status. In the reported campaigns, each supporting result contributed one point; two points marked a card as supported. Otherwise, at least three evaluated result–baseline pairs and a contradictory fraction ≥ 0.70 marked it as refuted (archived as `contradicted`). Neutral records remain in that denominator. Support takes precedence if both thresholds are met. A card meeting neither threshold remains active until its specified lifetime is reached, counted from creation in planning updates (default ten; allowed range one to twenty), and is then retired. Expired cards with no linked results are retired before the Planner call; other status updates follow the end-of-update evaluation above. Supported, refuted and retired states are terminal.

D. Supplementary experiments and campaign analyses

The analyses below examine throughput and robustness (Figure 2), archived follow-up decisions (Figure 3), allocation and recovery (Figure 4), and post-hoc confidence and structural diversity (Figures 2c and 5).

bioRxiv preprint doi: https://doi.org/10.64898/2026.09.22.753604; this version posted September 24, 2026. The copyright holder for this preprint (which was not certified by peer review) is the author/funder, who has granted bioRxiv a license to display the preprint in perpetuity. It is made available under a CC-BY 4.0 International license.

D.1. Throughput and robustness

D.1.1. Repeated campaigns

Across the three independently initialized campaigns per controller on CD45, SC2RBD and CbAgo, every T-REX campaign produced more final TM0.6 SUs than every PUCT or ϵ-greedy campaign on the same target (Figure 2d; Table S9). Section B.7 specifies the replication design. Figure S3 shows the online T-REX trajectories and separately marks the final recomputed counts; online counts can change when clustering is updated (Section C.4.2).

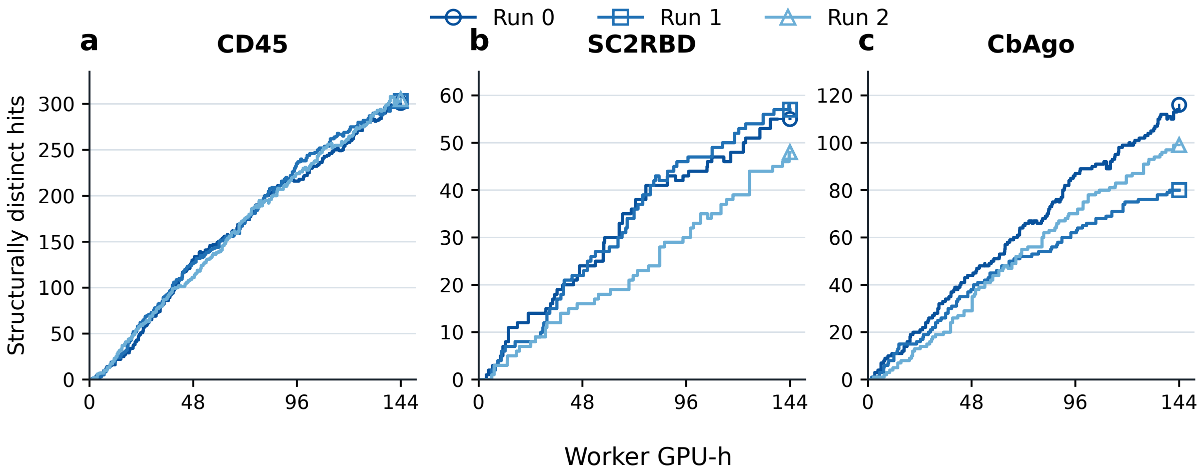

Figure S3 | **Online SU counts across three T-REX runs.** Each panel shows three independently initialized campaigns for one target under the prespecified 144 H100 worker-GPU-h denominator. Solid steps show the archived online TM0.6 SU counts, which can decrease when Foldseek clustering is updated; open symbols show final recomputed TM0.6 SU counts. Figure 2d and Supplementary Table S9 compare the corresponding adaptive controllers.

Table S9 | **Repeated adaptive-controller campaigns.** Final TM0.6 SU counts for three independently initialized campaigns of each adaptive controller (Run 0, Run 1 and Run 2 use controller seeds 0, 1 and 2, respectively) using the same campaign setup and prespecified denominator of 144 H100 worker GPU-h; the summary column is mean ± sample standard deviation (s.d.) after dividing each count by 144 worker GPU-h.

Target	Controller	Run 0	Run 1	Run 2	Mean ± s.d. rate
CD45	T-REX	301	303	305	2.104 ± 0.014
CD45	PUCT	69	122	27	0.505 ± 0.331
CD45	ϵ-greedy	27	19	28	0.171 ± 0.034
SC2RBD	T-REX	55	57	48	0.370 ± 0.033
SC2RBD	PUCT	40	37	38	0.266 ± 0.011
SC2RBD	ϵ-greedy	20	25	21	0.153 ± 0.018
CbAgo	T-REX	116	80	99	0.683 ± 0.125
CbAgo	PUCT	54	61	50	0.382 ± 0.039
CbAgo	ϵ-greedy	9	30	13	0.120 ± 0.077

D.1.2. Retrospective checkpoints and shorter-budget comparisons

Reconstructing accumulation curves. The curves in Figure 2a use final qualification and TM0.6 cluster assignments for the designs included under Section B.3. Adaptive curves assign each cluster to the earliest archived *EvidenceSummary* containing a qualified member; generator-only curves use the completion events specified in Table S10. PUCT HER2-AAV records arriving after the last summary are assigned to the 144-worker-GPU-h cutoff. A final horizontal segment holds the last assigned count constant to that cutoff without adding clusters. These retrospectively reconstructed

bioRxiv preprint doi: https://doi.org/10.64898/2026.09.22.753604; this version posted September 24, 2026. The copyright holder for this preprint (which was not certified by peer review) is the author/funder, who has granted bioRxiv a license to display the preprint in perpetuity. It is made available under a CC-BY 4.0 International license.

curves can differ from online cluster counts. Because the recorded events differ across methods, intermediate checkpoints describe accumulation rather than precise cross-method completion times.

At checkpoint B worker GPU-h, $N(B)$ counts final clusters assigned an event at or before B, and $N(B)/B$ gives the checkpoint rate (Table S10). Cluster assignments remain fixed; these are observations from campaigns run to 144 worker GPU-h, not campaigns independently stopped and finalized at B. At 10 worker GPU-h, T-REX had the largest count on six targets and tied BindCraft-only on HER2-AAV; it led on all seven at 50, 100 and 144 worker GPU-h. The across-target geometric-mean T-REX/PUCT count ratios were 3.94, 3.10, 2.73 and 2.43, respectively.

Table S10 | **Retrospective TM0.6 structure-unique accumulation at fixed compute checkpoints.** Entries give the cumulative number of final TM0.6 SUs assigned by the recorded curve event time, with SU per worker GPU-h in parentheses; bold marks the largest count within a target and checkpoint. The checkpoints are read from campaigns run to 144 worker GPU-h without new stopping, shutdown or clustering procedures at the earlier budgets. Adaptive curves use archived *EvidenceSummaries*; Complexa-only uses individual design completions; BindCraft-only uses output completions; and BoltzGen-only uses iteration completions. For the generator-only baselines, any required standardized AF2 evaluation occurred after the generation cutoff and is excluded from the generation budget. The event types differ across methods, so intermediate checkpoints do not measure comparable per-design completion times.

Target	Method	10 GPU-h	50 GPU-h	100 GPU-h	144 GPU-h
CD45	T-REX	**23 (2.30)**	**139 (2.78)**	**231 (2.31)**	**301 (2.09)**
	PUCT	3 (0.30)	23 (0.46)	51 (0.51)	69 (0.48)
	ϵ-greedy	4 (0.40)	15 (0.30)	21 (0.21)	27 (0.19)
	Complexa-only	6 (0.60)	22 (0.44)	39 (0.39)	50 (0.35)
	BindCraft-only	1 (0.10)	1 (0.02)	2 (0.02)	2 (0.01)
	BoltzGen-only	0 (0.00)	2 (0.04)	2 (0.02)	5 (0.03)
BetV1	T-REX	**5 (0.50)**	**27 (0.54)**	**43 (0.43)**	**64 (0.44)**
	PUCT	2 (0.20)	11 (0.22)	21 (0.21)	34 (0.24)
	ϵ-greedy	1 (0.10)	8 (0.16)	12 (0.12)	17 (0.12)
	Complexa-only	0 (0.00)	7 (0.14)	12 (0.12)	17 (0.12)
	BindCraft-only	0 (0.00)	1 (0.02)	3 (0.03)	3 (0.02)
	BoltzGen-only	0 (0.00)	0 (0.00)	11 (0.11)	15 (0.10)
CbAgo	T-REX	**11 (1.10)**	**44 (0.88)**	**86 (0.86)**	**116 (0.81)**
	PUCT	1 (0.10)	14 (0.28)	33 (0.33)	54 (0.38)
	ϵ-greedy	3 (0.30)	9 (0.18)	9 (0.09)	9 (0.06)
	Complexa-only	0 (0.00)	0 (0.00)	2 (0.02)	2 (0.01)
	BindCraft-only	8 (0.80)	24 (0.48)	40 (0.40)	52 (0.36)
	BoltzGen-only	0 (0.00)	20 (0.40)	29 (0.29)	37 (0.26)
HER2-AAV	T-REX	**9 (0.90)**	**64 (1.28)**	**144 (1.44)**	**204 (1.42)**
	PUCT	6 (0.60)	15 (0.30)	29 (0.29)	52 (0.36)
	ϵ-greedy	8 (0.80)	24 (0.48)	27 (0.27)	27 (0.19)
	Complexa-only	4 (0.40)	20 (0.40)	34 (0.34)	42 (0.29)
	BindCraft-only	**9 (0.90)**	41 (0.82)	79 (0.79)	104 (0.72)
	BoltzGen-only	0 (0.00)	0 (0.00)	0 (0.00)	2 (0.01)
SC2RBD	T-REX	**5 (0.50)**	**26 (0.52)**	**43 (0.43)**	**55 (0.38)**
	PUCT	1 (0.10)	12 (0.24)	25 (0.25)	40 (0.28)
	ϵ-greedy	3 (0.30)	17 (0.34)	20 (0.20)	20 (0.14)
	Complexa-only	0 (0.00)	0 (0.00)	0 (0.00)	0 (0.00)
	BindCraft-only	3 (0.30)	7 (0.14)	14 (0.14)	22 (0.15)
	BoltzGen-only	0 (0.00)	3 (0.06)	6 (0.06)	6 (0.04)
PDL1	T-REX	**53 (5.30)**	**229 (4.58)**	**379 (3.79)**	**482 (3.35)**
	PUCT	25 (2.50)	103 (2.06)	189 (1.89)	233 (1.62)
	ϵ-greedy	20 (2.00)	51 (1.02)	65 (0.65)	65 (0.45)
	Complexa-only	45 (4.50)	143 (2.86)	219 (2.19)	281 (1.95)
	BindCraft-only	27 (2.70)	108 (2.16)	173 (1.73)	231 (1.60)

Continued on next page

bioRxiv preprint doi: https://doi.org/10.64898/2026.09.22.753604; this version posted September 24, 2026. The copyright holder for this preprint (which was not certified by peer review) is the author/funder, who has granted bioRxiv a license to display the preprint in perpetuity. It is made available under a CC-BY 4.0 International license.

Supplementary Table continued

Target	Method	10 GPU-h	50 GPU-h	100 GPU-h	144 GPU-h
	BoltzGen-only	0 (0.00)	92 (1.84)	128 (1.28)	158 (1.10)
IL7RA	T-REX	**31 (3.10)**	**108 (2.16)**	**182 (1.82)**	**239 (1.66)**
	PUCT	7 (0.70)	38 (0.76)	67 (0.67)	95 (0.66)
	ϵ-greedy	13 (1.30)	37 (0.74)	40 (0.40)	40 (0.28)
	Complexa-only	19 (1.90)	67 (1.34)	125 (1.25)	167 (1.16)
	BindCraft-only	16 (1.60)	79 (1.58)	132 (1.32)	178 (1.24)
	BoltzGen-only	0 (0.00)	0 (0.00)	124 (1.24)	149 (1.03)

Bayesian-optimization comparison. The independently stopped 100-worker-GPU-h SMAC3 campaigns produced 329 final TM0.6 SUs across seven targets, compared with 1,108 at the retrospective 100-worker-GPU-h checkpoints of T-REX; T-REX had the larger count on every target (Table S11). SMAC3 stopped after approximately 25 h on four workers (setup in Section B.5.2); T-REX checkpoints come from three-worker campaigns planned for 144 worker GPU-h. The comparison matches worker compute but does not establish the outcome of independently stopping T-REX at 100 worker GPU-h.

Table S11 | **No-LLM SMAC3 Bayesian-optimization comparator at 100 worker GPU-h.** Each target–method cell gives the TM0.6 SU count, with SU per worker GPU-h in parentheses. SMAC3 values are archived endpoints from independently stopped campaigns that used four H100 workers for 25 h and ended at 99.85–100.00 worker GPU-h. Values for T-REX, PUCT, ϵ-greedy and the strongest generator-only reference are retrospective 100-GPU-h checkpoints from Supplementary Table S10. The SMAC3 policy and execution setup are specified in Section B.5.2. The strongest generator-only column selects the largest of Complexa-only, BindCraft-only and BoltzGen-only at the same checkpoint, with the family shown in parentheses. The final row pools counts across seven targets. Bold marks the largest count per target.

Target	T-REX	SMAC3 Bayesian opt.	PUCT	ϵ-greedy	Strongest generator-only
CD45	**231 (2.31)**	11 (0.11)	51 (0.51)	21 (0.21)	39 (0.39; Complexa)
BetV1	**43 (0.43)**	19 (0.19)	21 (0.21)	12 (0.12)	12 (0.12; Complexa)
CbAgo	**86 (0.86)**	35 (0.35)	33 (0.33)	9 (0.09)	40 (0.40; BindCraft)
HER2-AAV	**144 (1.44)**	29 (0.29)	29 (0.29)	27 (0.27)	79 (0.79; BindCraft)
SC2RBD	**43 (0.43)**	21 (0.21)	25 (0.25)	20 (0.20)	14 (0.14; BindCraft)
PDL1	**379 (3.79)**	173 (1.73)	189 (1.89)	65 (0.65)	219 (2.19; Complexa)
IL7RA	**182 (1.82)**	41 (0.41)	67 (0.67)	40 (0.40)	132 (1.32; BindCraft)
Total	**1,108**	329	415	194	535

D.1.3. Compute accounting sensitivity

LLM call time and serving cost. Across 3,125 archived model calls, end-to-end latency totalled 84,533 s (23.48 h; mean, 27.05 s per call), equivalent to 6.99% of the aggregate nominal campaign wall time ($7 \times 48 = 336$ h). LLM serving used a separate GPU while worker jobs could continue, so this sum measures neither added campaign duration nor delays in queue refill or worker idle time. The primary worker-budget definition and endpoint-inclusion rules are given in Section B.3.

A total-compute sensitivity added the full 48-h LLM-server reservation to each campaign's reconstructed worker time: 336 H100 GPU-h across seven campaigns, or 14.3 times the aggregate call latency. This charges approximately 192 H100 GPU-h per T-REX campaign while retaining its three-worker execution. T-REX remained ahead of PUCT on every target under this accounting (1.03–3.27-fold in TM0.6 SU per total H100 GPU-h; geometric mean, 1.82-fold; Table S12a).

A second sensitivity retained the full server charge and added archived post-cutoff AF2 evaluation time to each generator-only baseline before selecting the strongest adjusted baseline per target. Online T-REX evaluation was already included in worker time; Complexa supplied the common AF2

bioRxiv preprint doi: https://doi.org/10.64898/2026.09.22.753604; this version posted September 24, 2026. The copyright holder for this preprint (which was not certified by peer review) is the author/funder, who has granted bioRxiv a license to display the preprint in perpetuity. It is made available under a CC-BY 4.0 International license.

measurements inline and required no separate post-cutoff evaluation charge. T-REX again led on every target (1.07–4.52-fold; geometric mean, 1.90-fold; Table S12b).

Table S12 | **Compute-accounting sensitivities.** **a**, For T-REX, total charged time adds the full nominal 48 H100 GPU-h reservation of the separate vLLM server to reconstructed worker time; this is a total-compute charge, not a four-worker rerun. PUCT used no LLM server. **b**, The same full T-REX server charge is retained, and archived post-cutoff standardized AF2 evaluation time is added to every generator-only baseline before the strongest adjusted baseline is selected for each target. Online T-REX evaluation is already included in worker time; Complexa supplied the common AF2 qualification measurements inline and therefore had no separate post-cutoff AF2 charge. The T-REX counts, total charges and adjusted rates in part a also apply to part b. Rates use the primary TM0.6 SU counts. Rate ratios divide T-REX throughput by comparator throughput; values above one favor T-REX.

a. Full LLM-server charge versus PUCT

T-REX: worker charge plus the full 48-H100-GPU-hour server reservation

Target	T-REX SU	Worker GPU-h	Total GPU-h	SU/total GPU-h
CD45	301	143.8	191.8	1.570
BetV1	64	144.0	192.0	0.333
CbAgo	116	144.0	192.0	0.604
HER2-AAV	204	144.0	192.0	1.063
SC2RBD	55	144.0	192.0	0.286
PDL1	482	143.8	191.8	2.513
IL7RA	239	144.0	192.0	1.245

PUCT: no LLM-server charge

Target	PUCT SU	Total GPU-h	SU/total GPU-h	Rate ratio
CD45	69	143.7	0.480	3.27
BetV1	34	143.9	0.236	1.41
CbAgo	54	144.0	0.375	1.61
HER2-AAV	52	144.0	0.361	2.94
SC2RBD	40	144.0	0.278	1.03
PDL1	233	143.6	1.623	1.55
IL7RA	95	144.0	0.660	1.89

Geometric-mean throughput ratio: 1.82

b. Full server charge and post-cutoff baseline-evaluation charge

Target	Generator-only baseline	Baseline SU	Post-cutoff AF2 GPU-h	Total GPU-h	SU/total GPU-h	Rate ratio
CD45	Complexa-only	50	0.000	144.0	0.347	4.52
BetV1	Complexa-only	17	0.000	144.0	0.118	2.82
CbAgo	BindCraft-only	52	4.710	148.7	0.350	1.73
HER2-AAV	BindCraft-only	104	8.066	152.1	0.684	1.55
SC2RBD	BindCraft-only	22	1.234	145.2	0.151	1.89
PDL1	Complexa-only	281	0.000	144.0	1.951	1.29
IL7RA	Complexa-only	167	0.000	144.0	1.160	1.07

Geometric-mean throughput ratio: 1.90

D.1.4. Diversity and qualification-margin sensitivity

T-REX retained the highest cluster throughput on all seven targets under both MMseqs2 sequence clustering at 70% identity and Foldseek structural clustering at TM0.5, TM0.6 and TM0.8 (Figure 2b; Tables S13 and S14). These analyses use the same qualified design pools and the clustering settings in Section B.2; sequence clustering is applied before structural deduplication.

bioRxiv preprint doi: https://doi.org/10.64898/2026.09.22.753604; this version posted September 24, 2026. The copyright holder for this preprint (which was not certified by peer review) is the author/funder, who has granted bioRxiv a license to display the preprint in perpetuity. It is made available under a CC-BY 4.0 International license.

Table S13 | **MMseqs2 sequence-cluster throughput among qualified designs at 70% identity.** Target set: CD45, BetV1, CbAgo, HER2-AAV, SC2RBD, PDL1 and IL7RA. Binder-chain sequences from all designs satisfying the common qualification rule were clustered before Foldseek deduplication. Each rate divides the sequence-cluster count by the prespecified denominator of 144 H100 worker GPU-h. Foldseek TM0.6 SU is shown for structural context but does not define the sequence-cluster count. Bold and underline mark the best and second-best values within each target and numeric column.

Target	Method	Qualified	Foldseek SU	Seq. clusters	Seq. clusters/worker GPU-h
CD45	T-REX	**1,936**	**301**	**808**	**5.61**
CD45	PUCT	<u>248</u>	<u>69</u>	<u>130</u>	<u>0.90</u>
CD45	ϵ-greedy	132	27	63	0.44
CD45	Complexa-only	220	50	81	0.56
CD45	BindCraft-only	13	2	2	0.01
CD45	BoltzGen-only	22	5	22	0.15
BetV1	T-REX	**244**	**64**	**116**	**0.81**
BetV1	PUCT	<u>120</u>	<u>34</u>	<u>48</u>	<u>0.33</u>
BetV1	ϵ-greedy	69	17	32	0.22
BetV1	Complexa-only	42	17	17	0.12
BetV1	BindCraft-only	3	3	3	0.02
BetV1	BoltzGen-only	16	15	16	0.11
CbAgo	T-REX	**503**	**116**	**207**	**1.44**
CbAgo	PUCT	177	<u>54</u>	83	0.58
CbAgo	ϵ-greedy	31	9	14	0.10
CbAgo	Complexa-only	3	2	2	0.01
CbAgo	BindCraft-only	<u>182</u>	52	64	0.44
CbAgo	BoltzGen-only	114	37	<u>96</u>	<u>0.67</u>
HER2-AAV	T-REX	**859**	**204**	**504**	**3.50**
HER2-AAV	PUCT	310	52	78	0.54
HER2-AAV	ϵ-greedy	173	27	42	0.29
HER2-AAV	Complexa-only	156	42	57	0.40
HER2-AAV	BindCraft-only	<u>630</u>	<u>104</u>	<u>121</u>	<u>0.84</u>
HER2-AAV	BoltzGen-only	2	2	2	0.01
SC2RBD	T-REX	**252**	**55**	**100**	**0.69**
SC2RBD	PUCT	<u>163</u>	<u>40</u>	<u>70</u>	<u>0.49</u>
SC2RBD	ϵ-greedy	93	20	33	0.23
SC2RBD	Complexa-only	0	0	0	0.00
SC2RBD	BindCraft-only	101	22	28	0.19
SC2RBD	BoltzGen-only	8	6	8	0.06
PDL1	T-REX	**6,203**	**482**	**2,064**	**14.33**
PDL1	PUCT	920	233	775	5.38
PDL1	ϵ-greedy	360	65	137	0.95
PDL1	Complexa-only	<u>2,897</u>	<u>281</u>	927	6.44
PDL1	BindCraft-only	2,033	231	399	2.77
PDL1	BoltzGen-only	1,412	158	<u>1,326</u>	<u>9.21</u>
IL7RA	T-REX	**1,178**	**239**	**531**	**3.69**
IL7RA	PUCT	513	95	164	1.14
IL7RA	ϵ-greedy	152	40	75	0.52
IL7RA	Complexa-only	<u>1,086</u>	167	392	2.72
IL7RA	BindCraft-only	1,047	<u>178</u>	248	1.72
IL7RA	BoltzGen-only	656	149	<u>481</u>	<u>3.34</u>

bioRxiv preprint doi: https://doi.org/10.64898/2026.09.22.753604; this version posted September 24, 2026. The copyright holder for this preprint (which was not certified by peer review) is the author/funder, who has granted bioRxiv a license to display the preprint in perpetuity. It is made available under a CC-BY 4.0 International license.

Table S14 | **Sensitivity to the Foldseek clustering threshold.** Target set: CD45, BetV1, CbAgo, HER2-AAV, SC2RBD, PDL1 and IL7RA. Each rate divides the number of qualified structural clusters by the prespecified denominator of 144 H100 worker GPU-h; Qualified is the number of designs before Foldseek clustering. Bold and underline mark the best and second-best rate within each target and TM threshold; ties share a mark.

Target	Method	Qualified	TM0.5	TM0.6	TM0.8
CD45	T-REX	1,936	**200 (1.389)**	**301 (2.090)**	**471 (3.271)**
CD45	PUCT	248	<u>47 (0.326)</u>	<u>69 (0.479)</u>	<u>87 (0.604)</u>
CD45	ϵ-greedy	132	20 (0.139)	27 (0.188)	33 (0.229)
CD45	Complexa-only	220	37 (0.257)	50 (0.347)	70 (0.486)
CD45	BindCraft-only	13	2 (0.014)	2 (0.014)	2 (0.014)
CD45	BoltzGen-only	22	4 (0.028)	5 (0.035)	15 (0.104)
BetV1	T-REX	244	**47 (0.326)**	**64 (0.444)**	**78 (0.542)**
BetV1	PUCT	120	<u>27 (0.188)</u>	<u>34 (0.236)</u>	<u>36 (0.250)</u>
BetV1	ϵ-greedy	69	16 (0.111)	17 (0.118)	17 (0.118)
BetV1	Complexa-only	42	16 (0.111)	17 (0.118)	17 (0.118)
BetV1	BindCraft-only	3	3 (0.021)	3 (0.021)	3 (0.021)
BetV1	BoltzGen-only	16	13 (0.090)	15 (0.104)	16 (0.111)
CbAgo	T-REX	503	**81 (0.562)**	**116 (0.806)**	**150 (1.042)**
CbAgo	PUCT	177	36 (0.250)	<u>54 (0.375)</u>	60 (0.417)
CbAgo	ϵ-greedy	31	9 (0.062)	9 (0.062)	9 (0.062)
CbAgo	Complexa-only	3	2 (0.014)	2 (0.014)	2 (0.014)
CbAgo	BindCraft-only	182	<u>39 (0.271)</u>	52 (0.361)	61 (0.424)
CbAgo	BoltzGen-only	114	31 (0.215)	37 (0.257)	<u>64 (0.444)</u>
HER2-AAV	T-REX	859	**137 (0.951)**	**204 (1.417)**	**316 (2.194)**
HER2-AAV	PUCT	310	44 (0.306)	52 (0.361)	56 (0.389)
HER2-AAV	ϵ-greedy	173	27 (0.188)	27 (0.188)	27 (0.188)
HER2-AAV	Complexa-only	156	28 (0.194)	42 (0.292)	55 (0.382)
HER2-AAV	BindCraft-only	630	<u>68 (0.472)</u>	<u>104 (0.722)</u>	<u>119 (0.826)</u>
HER2-AAV	BoltzGen-only	2	2 (0.014)	2 (0.014)	2 (0.014)
SC2RBD	T-REX	252	**34 (0.236)**	**55 (0.382)**	**77 (0.535)**
SC2RBD	PUCT	163	<u>31 (0.215)</u>	<u>40 (0.278)</u>	<u>47 (0.326)</u>
SC2RBD	ϵ-greedy	93	16 (0.111)	20 (0.139)	21 (0.146)
SC2RBD	Complexa-only	0	0 (0.000)	0 (0.000)	0 (0.000)
SC2RBD	BindCraft-only	101	16 (0.111)	22 (0.153)	28 (0.194)
SC2RBD	BoltzGen-only	8	4 (0.028)	6 (0.042)	7 (0.049)
PDL1	T-REX	6,203	**270 (1.875)**	**482 (3.347)**	**957 (6.646)**
PDL1	PUCT	920	147 (1.021)	233 (1.618)	402 (2.792)
PDL1	ϵ-greedy	360	46 (0.319)	65 (0.451)	80 (0.556)
PDL1	Complexa-only	2,897	<u>157 (1.090)</u>	<u>281 (1.951)</u>	<u>592 (4.111)</u>
PDL1	BindCraft-only	2,033	135 (0.938)	231 (1.604)	357 (2.479)
PDL1	BoltzGen-only	1,412	70 (0.486)	158 (1.097)	578 (4.014)
IL7RA	T-REX	1,178	**153 (1.062)**	**239 (1.660)**	**382 (2.653)**
IL7RA	PUCT	513	67 (0.465)	95 (0.660)	120 (0.833)
IL7RA	ϵ-greedy	152	30 (0.208)	40 (0.278)	65 (0.451)
IL7RA	Complexa-only	1,086	101 (0.701)	167 (1.160)	304 (2.111)
IL7RA	BindCraft-only	1,047	<u>106 (0.736)</u>	<u>178 (1.236)</u>	240 (1.667)
IL7RA	BoltzGen-only	656	79 (0.549)	149 (1.035)	<u>377 (2.618)</u>

The qualification-margin analysis instead holds the final TM0.6 clusters fixed and retains those whose selected representative has $m_{\min} \geq \tau$ (Figure S4; Section B.2). Each representative maximizes $m_{\min}$ within its cluster, so a retained cluster contains at least one design meeting all three tightened margins. Cluster membership is not recomputed after filtering. At $\tau = 0$, the qualified pool recovers the primary count while retaining the original strict scRMSD cutoff.

Across 63 target–margin combinations ($\tau = 0, 0.05, \ldots, 0.40$), T-REX exceeded PUCT in 50, tied in ten, including seven zero–zero ties, and was lower in three, each by one SU. Thus, the throughput advantage persisted across most tested margin cutoffs, with ties and reversals as fewer clusters remained. This margin-retention analysis is distinct from reclustering at alternative TM thresholds.

bioRxiv preprint doi: https://doi.org/10.64898/2026.09.22.753604; this version posted September 24, 2026. The copyright holder for this preprint (which was not certified by peer review) is the author/funder, who has granted bioRxiv a license to display the preprint in perpetuity. It is made available under a CC-BY 4.0 International license.

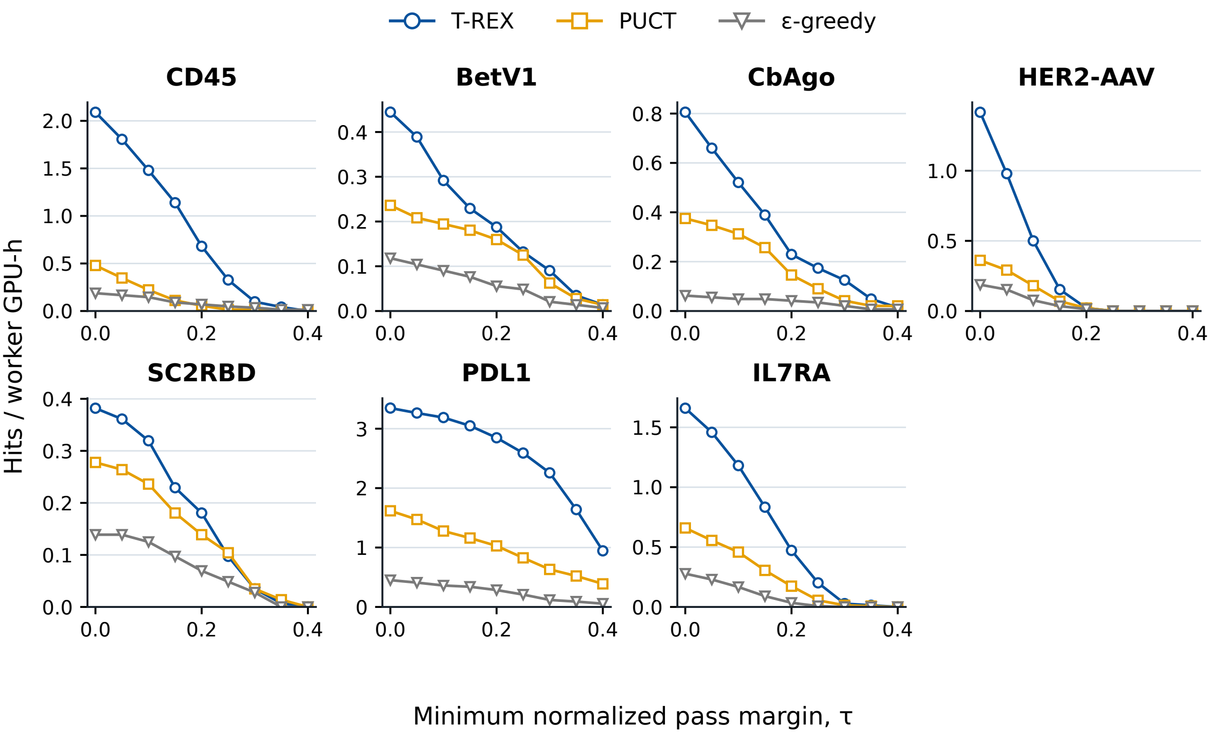

Figure S4 | **Sensitivity to jointly tightening the three qualification criteria.** Fixed final Foldseek TM0.6 clusters per worker GPU-h whose selected representative meets the requirement that the normalized pLDDT, iPAE and scRMSD pass margins are all at least τ; $\tau = 0$ recovers the primary qualification rule. Rates use the prespecified denominator of 144 H100 worker GPU-h.

D.2. Evidence-linked decisions and worked examples

D.2.1. Evidence at an archived planning update

This example follows HER2-AAV planning update 6 from archived evidence to the reduced LLM input. Update numbers identify planning steps, not elapsed time. The JSON excerpts here and in Section D.2.2 retain field names, nesting, nulls and booleans; descriptive aliases (`example_design`, `rescue_hypothesis`, `rescue_candidate`) replace archive identifiers. Selected measurements are rounded to four decimals; omitted fields do not imply missing measurements.

bioRxiv preprint doi: https://doi.org/10.64898/2026.09.22.753604; this version posted September 24, 2026. The copyright holder for this preprint (which was not certified by peer review) is the author/funder, who has granted bioRxiv a license to display the preprint in perpetuity. It is made available under a CC-BY 4.0 International license.

Archived EvidenceSummary excerpt.


```
{
  "tick_id": "update_6",
  "target_id": "HER2-AAV",
  "state_label": "low_evidence",
  "strict_count": 0,
  "run_su_count": 0,
  "near_miss_count": 2,
  "completed_children": 36,
  "pending_children": 3,
  "axis_stats": {
    "pLDDT": {"n": 20, "pass_count": 4},
    "iPAE": {"n": 20, "pass_count": 0},
    "binder_scRMSD": {"n": 20, "pass_count": 8}
  },
  "diagnostic_chain_backlog": {
    "by_family": {
      "boltzgen": {
        "accepted_artifacts": 16,
        "completed_refilters": 4,
        "pending_chain_candidates": 12
      }
    }
  },
  "foldseek_su_status": "no_strict",
  "foldseek_su_coverage": null,
  "near_miss_dedup_status": "ok",
  "near_miss_dedup_coverage": 1.0,
  "exemplars": [
    {
      "result_id": "example_design",
      "kind": "near_miss",
      "dominant_deficit_axis": "iPAE",
      "metrics": {"pLDDT": 90.8924, "iPAE": 0.2397, "binder_scRMSD": 0.7215}
    }
  ]
}
```


Qualification and pending evaluation. The campaign had no qualified designs or online SUs, two near-miss clusters and the deterministically assigned campaign state `low_evidence` (Section C.4.5). The illustrated near miss passed pLDDT (90.8924) and scRMSD (0.7215 Å) but its normalized iPAE (0.2397) exceeded the cutoff. The LLM later incorrectly described scRMSD as failing; qualification used the recorded measurements (Section D.2.2). Qualified-pool clustering had no structures (`no_strict`, `null` coverage), whereas near-miss clustering was complete (`ok`, coverage 1.0). Of sixteen BoltzGen designs, four had completed standardized AF2 evaluation and twelve awaited evaluation under the admission rules in Section C.8.1; their qualification outcomes were still unknown.

Criterion summaries and structural duplication. Each criterion had twenty measurements. For pLDDT, normalized iPAE and binder scRMSD, respectively, pass/near-pass/fail counts were 4/8/8, 0/5/15 and 8/2/10; medians were 85.7853, 0.3446 and 2.2067 Å. These criterion-specific categories follow Section C.4.1. Paired pLDDT–iPAE counts were zero both-pass, eight both-fail, zero pass/fail or fail/pass, and one both-near-pass. Mixed near-pass combinations are omitted from these five patterns, so their counts need not sum to twenty. Among recent cluster-assigned records, $f_{\text{dup}} = 0.75$

bioRxiv preprint doi: https://doi.org/10.64898/2026.09.22.753604; this version posted September 24, 2026. The copyright holder for this preprint (which was not certified by peer review) is the author/funder, who has granted bioRxiv a license to display the preprint in perpetuity. It is made available under a CC-BY 4.0 International license.

(Eq. (S3)) and the largest cluster contained 20% of records. This structural duplication measure includes unqualified designs and does not confer SU credit.

Recorded results and compute. The 36 *ResultRecords* comprised sixteen Complexa Beam outputs, sixteen BoltzGen outputs and four standardized AF2 evaluations; all had `ok` status. The family-level `attempts` and `completions` therefore counted records, not job launches (Section C.3). Their recorded costs were 0.3216, 0.1362 and 0.0363 worker GPU-h, respectively, totaling 0.4941 worker GPU-h. The three `pending_children` were running jobs. Elapsed worker-slot time was 1.0003 GPU-h, whereas the stall-time signal was 0.9673 worker GPU-h and six updates had passed without an SU. This signal combines recorded cost and running-worker time, with a zero cost reference before the first SU (Section C.4.2); it differs from elapsed campaign time.

Complexa Beam contributed both near-miss clusters. All three qualification measurements were available for Complexa Beam and AF2-evaluated outputs, but absent from native BoltzGen outputs. A Complexa Beam `recipes` entry grouped eight descendants under a near-miss outcome, with beam width 4, four branches and four samples; the illustrated design came from the initial Complexa Beam job. Auxiliary and alternative-model measurements provided context without affecting qualification or SU credit (Section C.4.3).

Four outcome types within one campaign. These records illustrate the distinctions in Figure 1b: a later Complexa FK steering job timed out without outputs or measurements; twelve BoltzGen designs awaited evaluation; and the illustrated Complexa Beam design missed the iPAE cutoff. Between updates 11 and 12, qualified designs increased from 42 to 43 while online SUs remained at four, with complete, successful qualified-pool clustering. This last case added qualification without increasing the online SU count.

Reduced evidence input to the LLMs. The following excerpt was reconstructed using the input-reduction rules in Section C.5.2; it is not a saved historical prompt. The target identifier is omitted, the 36 parent identifiers become a count and availability flag, and cumulative objective fields are grouped in `objective_summary`. Parent identifiers remained available for candidate construction. HypothesisCards and, for the Supervisor, candidate jobs were supplied separately. The excerpt shows selected evidence fields rather than either role's complete input.


```
{
  "tick_id": "update_6",
  "state_label": "low_evidence",
  "parent_artifact_result_count": 36,
  "parent_artifacts_available": true,
  "objective_summary": {
    "live_su_tm06": 0,
    "raw_strict_count": 0,
    "completed_worker_gpu_h_total": 0.4941,
    "worker_wall_gpu_h_total": 1.0003
  }
}
```


D.2.2. From proposal to execution and feedback

The trace continues from update 6 in Section D.2.1, following one proposal through selection, execution and feedback. JSON excerpts show archived HypothesisCard and ActionCandidate fields,

bioRxiv preprint doi: https://doi.org/10.64898/2026.09.22.753604; this version posted September 24, 2026. The copyright holder for this preprint (which was not certified by peer review) is the author/funder, who has granted bioRxiv a license to display the preprint in perpetuity. It is made available under a CC-BY 4.0 International license.

using the same descriptive aliases; they are not raw LLM responses.

1. **Planner proposal.** The card proposed a **Rescue** test: decrease normalized iPAE and binder scRMSD while preserving pLDDT. Two other cards proposed BindCraft and Complexa best-of-n exploration. The excerpt shows the cited evidence and suggested settings; Section C.5.2 defines the Planner output.


```
{
  "hypothesis_id": "rescue_hypothesis",
  "evidence_refs": ["current_evidence", "beam_route", "example_design"],
  "config_delta_suggestions": {
    "complexa_beam": {
      "beam_width": 8,
      "n_branch": 4,
      "nsamples": 4,
      "nsteps": 400,
      "reward_i_ptm_weight": 1.5,
      "sc_scale_noise": 0.3
    }
  }
}
```


2. **Candidate construction and validation.** Check + Build removed `reward_i_ptm_weight`: the largest observation count among interface measurements was 20, below the required 32 (Section C.6.2). The remaining settings passed family-availability, configuration, workload and resource checks. The cited design served as the comparison baseline; it was not supplied as a parent structure to this generation job.


```
{
  "candidate_id": "rescue_candidate",
  "hypothesis_ids": ["rescue_hypothesis"],
  "baseline_result_id": "example_design",
  "parent_result_id": null,
  "config_delta": {
    "beam_width": 8,
    "n_branch": 4,
    "nsamples": 4,
    "nsteps": 400,
    "sc_scale_noise": 0.3
  }
}
```


3. **Supervisor prioritization.** The Supervisor ranked Complexa Beam **Rescue** first, BindCraft **Explore** second and Complexa best-of-n **Explore** third. Its confidence of 0.50 activated the conditional allocation bounds, but the proposed **Rescue**/**Explore**/**eXploit** shares remained $0.40/0.60/0.00$ after adjustment; no fallback was used (Section C.7.1). These are desired shares of subsequent starts, not GPU-time fractions.
4. **Queue admission.** One queue position was available for selection, although all three workers were running jobs. The selector followed the Supervisor's current ranking across priorities and admitted the first-ranked job; the other two were not selected (Section C.7.2). The recorded `launched` status denotes queue admission, with execution confirmed separately.

bioRxiv preprint doi: https://doi.org/10.64898/2026.09.22.753604; this version posted September 24, 2026. The copyright holder for this preprint (which was not certified by peer review) is the author/funder, who has granted bioRxiv a license to display the preprint in perpetuity. It is made available under a CC-BY 4.0 International license.

5. **Independent evaluation and confirmed starts.** At the same update, the evaluation-admission rules admitted a previously generated BindCraft design for standardized AF2 evaluation (Section C.8.1). DispatchRecords confirmed that this evaluation and the selected Complexa Beam job passed start-time checks and started. At update 7, the campaign recorded its first qualified design and online SU, from the BindCraft evaluation. The campaign state was `productive_duplicate`, and recorded cost totaled 1.1468 worker GPU-h. This first SU did not come from the selected Complexa job.
   The BindCraft route had one standardized AF2 evaluation, cost 0.3491 route GPU-h, and general/recent-productivity statuses `healthy/productive` (Section C.4.4). With one qualified cluster, its quality-score median and lower quartile both equaled 0.3670; its auxiliary diagnostic-improvement score was 1.0 (Section C.4.3).
6. **Recorded outputs.** Links to the producing candidate job identified 32 ResultRecords with `ok` status, of which eight met all three qualification criteria. Their update identifier refers to the producing job's start, not result arrival (Section C.3). These are result counts, distinct from jobs, SUs and hypothesis-support points.
7. **Hypothesis feedback.** At update 9, the card had 32 evaluated descendants, zero support points and zero contradiction points. Qualification did not establish the predicted changes relative to the comparison baseline (Section C.9). Because baseline scRMSD already passed and the card specified no absolute improvement threshold, support required the default 1 Å decrease from 0.7215 Å—an unattainable change. The already-passing criterion also prevented contradiction points under the rule requiring contradiction of every prediction. The card remained active and was retired at update 16, after its ten-update lifetime.

D.2.3. Archived decision traces and use of earlier results

The three cases in Figure 3 were selected retrospectively to illustrate follow-up tests prompted by an unsuccessful test, repeated near misses or a parent-specific failure. For every case described here, we linked the proposal, candidate job and launch decision to a confirmed job start and subsequent results. Qualification counts and displayed pLDDT, iPAE and scRMSD values came from standardized AF2 records; online TM0.6 SU gains came from the linked campaign summaries. The HER2-AAV and CbAgo panels compare route-level evidence and output (Figure 3a,b), whereas PDL1 follows a direct ProteinMPNN parent–descendant line (Figure 3c). Batch membership and route-level SU attribution were assessed separately: a job could yield multiple designs, and a route summary could include multiple jobs.

For visualization, output complexes were rigidly aligned on the target to the corresponding evidence or parent complex. The illustrated PDL1 descendant differed from its parent at 35 of 58 residues and had a target-aligned binder Cα RMSD of 0.714 Å relative to its parent. Qualification scRMSD uses the same target-based alignment but compares a generated complex with its AF2 prediction, rather than a parent with its redesigned descendant.

Within-campaign evidence reuse across seven targets. These retrospectively selected cases include unsuccessful reuse and do not estimate success rates. Counts distinguish qualified designs from final Foldseek TM0.6 representatives directly linked to the job; online configuration-level outcomes are identified separately (Section C.4.2). Rates and duplicate fractions describe the online evidence available at the time of the proposal (Section C.4). Card support and refutation assess numerical predictions, not causal explanations; retirement ends a card's lifetime without either finding (Section C.9).

bioRxiv preprint doi: https://doi.org/10.64898/2026.09.22.753604; this version posted September 24, 2026. The copyright holder for this preprint (which was not certified by peer review) is the author/funder, who has granted bioRxiv a license to display the preprint in perpetuity. It is made available under a CC-BY 4.0 International license.

CD45: continuing a productive route and reusing it for Rescue. A Complexa route with adjusted generator-internal reward weights had a recent rate of 2.32 SU/GPU-h, whereas the default route had no recent SU and a duplicate fraction of 0.81. The controller continued the productive Complexa Beam route with its recorded refinement and interface-related weights. A later Rescue proposal cited the same route and weight pair for an interface-limited near miss, testing whether that configuration remained useful for a related case. The continued-production job yielded 27 qualified designs and three final representatives; the later Rescue job yielded eleven qualified designs and one final representative.

SC2RBD: increasing diversity within a productive generator. The BindCraft route requesting eight native accepted designs had yielded 1.40 recent SU/GPU-h, but all recently clustered designs occupied one structural cluster. A route requesting sixteen native accepted designs subsequently yielded four SUs across four clusters. The proposed test increased the requested native accepted-design count from eight to sixteen while retaining the interface-weight configuration, then reused that configuration after the narrower route became redundant again. The initial job yielded nineteen qualified descendants and three final representatives; the later repeat yielded ten qualified descendants and one final representative.

PDL1: testing an intermediate sampling setting. A backbone sampling-noise value of 0.30 had the highest recent rate, 6.36 SU/GPU-h, but a duplicate fraction of 0.84. A value of 0.20 had lower yield and greater structural duplication, whereas the tested 0.40 configuration had reduced productivity. The controller tested 0.35, within the permitted bounds, while retaining the other route settings to examine a possible trade-off between production and diversification. The job yielded thirty qualified designs and five final representatives.

HER2-AAV: revising a hypothesis after an unsuccessful test. Complexa's native metrics for FK steering indicated strong interface confidence (median ipTM 0.77) but lower fold confidence (median pLDDT 83.4). An earlier pLDDT reward-weight test yielded no qualified designs, and its card was refuted. A subsequent proposal tested sequence–backbone compatibility by retaining FK steering and switching to sequence hallucination (Figure 3a). Five designs qualified. The first outcome summary for the same route and configuration reported three online SUs; this count is not attributable to the individual job. Feedback counted all 32 evaluable designs as support under the card-specific improvement and preservation tests, which are distinct from qualification.

BetV1: revisiting a generator with earlier structural diversity. Complexa had become duplicate-rich. BindCraft had produced 33 SUs earlier with greater structural diversity, although it had produced none in the recent window. The controller revisited BindCraft requesting eight native accepted designs and using positive helicity, ipTM and pLDDT weights to test whether another generator family could restore novelty. The job yielded seventeen qualified designs and two final representatives. Its HypothesisCard was retired at the lifetime limit.

BetV1: unsuccessful reuse of an earlier configuration. An interface-confidence Rescue proposal cited an earlier configuration that had produced qualified designs. The controller reused Complexa Beam with sequence hallucination, the maximum permitted interface-confidence reward weight and backbone sampling noise 0.50 for another near miss. None of sixteen linked designs qualified. The

bioRxiv preprint doi: https://doi.org/10.64898/2026.09.22.753604; this version posted September 24, 2026. The copyright holder for this preprint (which was not certified by peer review) is the author/funder, who has granted bioRxiv a license to display the preprint in perpetuity. It is made available under a CC-BY 4.0 International license.

HypothesisCard was retired at the lifetime limit. Thus, reuse of a previously productive configuration did not guarantee qualification.

CbAgo: repeating the highest-yield active route. An FK steering route with a negative iPAE reward weight had the highest recent rate among active configurations, 2.72 SU/GPU-h, and had previously yielded a high proportion of qualified designs. The controller relaunched that route with its recorded refinement and interface-related weights to test whether it continued to add structural clusters. This job, distinct from the one in Figure 3b, yielded eight qualified designs and one final representative.

IL7RA: continuing production despite a high duplicate fraction. BindCraft had recent throughput of 1.46 SU/GPU-h and a cumulative online count of 57 SUs despite a duplicate fraction of 0.80. The controller continued the route requesting two native accepted designs while other jobs tested alternatives. Both linked designs qualified and supported the card's numerical predictions.

D.3. Controller behavior and intervention

D.3.1. LLM use and interface checks

Model calls and output validation. The seven primary campaigns recorded 3,125 LLM calls: 1,719 Planner and 1,406 Supervisor calls (Table S15, part A). Seven Supervisor responses failed structured-output validation, representing 0.22% of all model calls. All seven were parsed as JSON, but six cited evidence references outside the allowed set and one used an invalid resource class. These rejected responses triggered deterministic fallback; Sections C.5.2 and C.7.1 describe validation and response handling.

Selection paths and confirmed starts. Model-call validation and fallback during selection have different denominators. We counted unique planning updates in *SupervisorDecision* records and linked each confirmed generation or redesign start through its *LaunchDecision* to the selecting update. Of 1,719 updates, 1,398 used the current Supervisor candidate ranking and 321 (18.7%) used fallback. These comprised 313 updates without hypotheses or candidates for a Supervisor call, seven with rejected Supervisor responses and one carrying a fallback reason from an empty Planner-card list despite a valid Supervisor response. The counts include updates that started no generation or redesign job.

All 1,454 generation or redesign starts linked to a recorded selection: 1,446 to updates using the Supervisor ranking and eight (0.55%) to fallback. The 3,160 separately admitted AF2 starts were excluded from this denominator. The selection path does not establish that the LLM proposed a job: fixed construction rules can add candidates to either path (Sections C.6 and C.7).

Controlled interface checks. The Planner tests used five synthetic evidence situations: productive work, concentrated near misses, stall, ambiguous evidence and qualified but structurally redundant output. Supervisor tests used the same situations except ambiguous evidence, with five calls per situation for each role. Checks required valid parsed outputs; normalized Supervisor allocation proportions also had to satisfy case-specific expectations: productive, $X \geq 0.45$ and $E \leq 0.30$; near-miss-enriched, $R \geq 0.40$; stalled, $E \geq 0.25$ and $X \leq 0.45$; structurally redundant, $E \geq 0.25$. All output-validity and allocation checks passed (Table S15, part B).

bioRxiv preprint doi: https://doi.org/10.64898/2026.09.22.753604; this version posted September 24, 2026. The copyright holder for this preprint (which was not certified by peer review) is the author/funder, who has granted bioRxiv a license to display the preprint in perpetuity. It is made available under a CC-BY 4.0 International license.

Repeatability and sampling-sensitivity tests used one fixed productive-evidence case with five candidate jobs: two **eXploit**, two **Rescue** and one **Explore**. Each test made ten Supervisor calls, at temperature 0 for repeatability and at temperatures 0.00–0.45 in 0.05 increments for sampling sensitivity. Within each test, all pairs of valid responses were compared using within-priority first-ranked-candidate agreement, Kendall correlation and the sum of absolute allocation-share differences, as defined in Table S15, part B. Three sampling-sweep responses omitted one Rescue candidate, so the reported agreement does not establish identical full rankings. These checks assess output validity and repeatability for the tested inputs, without validating explanations or establishing biological accuracy, campaign-wide reliability or a causal benefit of LLM reasoning.

Table S15 | **LLM calls and controlled interface checks.** Part A counts model calls, including valid Planner abstentions, but excludes skipped calls and deterministic advisory records. Part B uses synthetic inputs. Agreement is the fraction of pairwise comparisons within priorities with the same first-ranked candidate. Kendall correlation requires identical candidate sets with at least two jobs. Allocation difference is the sum of absolute differences across the R/E/X shares. Fixed handling of invalid or unavailable jobs is described in Sections C.5.2, C.6 and C.8.2.

A. Model calls and structured-output validation

Target	Model calls	Planner	Supervisor	Validation failures
CD45	468	267	201	0
BetV1	355	214	141	0
CbAgo	698	391	307	0
HER2-AAV	273	148	125	0
SC2RBD	179	90	89	0
PDL1	687	363	324	3
IL7RA	465	246	219	4
Total	3,125	1,719	1,406	7 (0.22%)

The model-call count includes 201 valid Planner abstentions. The archives also contain 313 skipped Supervisor calls and 1,517 deterministic advisory records, excluded from that count. Model calls used 114,105,382 read and 4,430,504 write tokens. Latency and resource-accounting sensitivity are reported in Section D.1.3 and Table S12.

B. Controlled interface checks

Check	Evidence	Result
Standard cases	Five Planner and four Supervisor cases, five repeats each	Planner 25/25 and Supervisor 20/20 valid; all case-specific allocation checks passed.
Repeatability	One fixed Supervisor case, ten calls at temperature 0	10/10 valid; within-priority first-candidate agreement and minimum eligible Kendall correlation 1.00; maximum summed allocation-share difference 0.00.
Sampling sensitivity	Same case at temperatures 0.00–0.45	10/10 valid; within-priority first-candidate agreement and minimum eligible Kendall correlation 1.00; maximum summed allocation-share difference 0.10.

D.3.2. Campaign-wide allocation and realized action-space use

Figure 4a groups 1,454 confirmed generation and redesign starts by campaign-state label and recorded R/E/X priority. Within-state percentages use all generation and redesign starts in that state as the denominator; required AF2 evaluations are excluded. The main-text percentages correspond to 48/65 **Explore** starts in low evidence, 41/80 **Rescue** starts across stall and deep stall combined, and 930/1,133 **eXploit** starts in productive-with-duplication states. No generation or redesign start was recorded in structural duplicate collapse. The state rules are defined in Supplementary Section C.4.5; panels c,d instead use the retrospective annotations described below.

Evidence-focus annotation. Each start was linked to structured decision text by target and candidate-job identifier, retaining repeated starts as separate events. The four annotation categories

bioRxiv preprint doi: https://doi.org/10.64898/2026.09.22.753604; this version posted September 24, 2026. The copyright holder for this preprint (which was not certified by peer review) is the author/funder, who has granted bioRxiv a license to display the preprint in perpetuity. It is made available under a CC-BY 4.0 International license.

were interface confidence, fold confidence, backbone consistency and structural redundancy. For linked decisions, manual annotations covering 27 planning decisions (35 starts) took precedence over text matching. Otherwise, case-insensitive matching first examined the proposed-job description (`what`). Only when this field matched no category did matching examine the combined evidence and rationale fields (`observed_signal`, `inference`, `why` and `action_implication`). One matched category assigned a single focus; matches to multiple categories assigned a multi-focus label. Starts with no match or no linked decision remained unassigned.

Panels c,d include the 1,305 starts assigned a single focus, excluding multi-focus and unassigned starts. The Figure 4 label *backbone consistency* denotes structural self-consistency, including wording about scRMSD, pose error or backbone drift (Section B.1). These annotations describe cited evidence, not independently diagnosed design limitations; annotation was not blinded and inter-rater agreement was not assessed.

Supplementary Table S16 reports measured qualification failures and structural redundancy separately from these annotations of decision text. Associations among campaign state, evidence focus and priority are descriptive: deterministic state rules, candidate construction and allocation bounds also influence realized starts.

Table S16 | **Target-level qualification and structural-redundancy profiles in the seven primary T-REX campaigns.** Each percentage is the fraction of standardized AF2-evaluated designs that failed the named criterion; designs can fail more than one criterion. Qualified designs per final TM0.6 SU reports structural repetition after the common qualification rule and final Foldseek clustering.

Target	Evaluated	iPAE fail (%)	pLDDT fail (%)	scRMSD fail (%)	Qualified	Final TM0.6 SUs	Qualified/SU
CD45	6,291	67.6	25.7	25.4	1,936	301	6.43
BetV1	2,310	84.0	72.9	51.7	244	64	3.81
CbAgo	5,170	89.1	62.6	43.5	503	116	4.34
HER2-AAV	3,733	73.0	62.8	14.8	859	204	4.21
SC2RBD	1,906	79.3	82.5	42.3	252	55	4.58
PDL1	9,099	26.3	29.5	10.5	6,203	482	12.87
IL7RA	3,163	51.0	50.1	24.5	1,178	239	4.93

Follow-up job types. For Figure 4d, jobs were classified in the following order. ProteinMPNN jobs were assigned to candidate redesign, explicit repeats of archived routes to recorded-route replay, and the first start of a generation family within each target campaign to first method use. For the remaining jobs, case-insensitive matching used the proposed-job description, or the recorded configuration when that description was absent. A match to `sequence[_ -]?hallucination` assigned candidate redesign; otherwise, `reward`, `weight`, `penalty` or `objective` assigned scoring-weight adjustment. All remaining jobs were assigned sampling or search adjustment. This order makes the categories mutually exclusive; the text-based categories describe proposed jobs and do not establish that every matched job changed scoring weights or sampling settings.

Realized action-space use. The seven primary T-REX campaigns used all six registered generation families and ProteinMPNN redesign, spanning 161 target-specific configurations and 1,454 confirmed generation or redesign starts (Supplementary Table S17). The table pools direct SU attribution across targets; Supplementary Figure S5 shows the target-level counts. Each final SU is attributed to the family that produced its selected representative, rather than all jobs that contributed to its ancestry. This attribution does not measure causal contribution or cost-normalized efficiency. Permitted configuration ranges are given in Supplementary Table S5.

bioRxiv preprint doi: https://doi.org/10.64898/2026.09.22.753604; this version posted September 24, 2026. The copyright holder for this preprint (which was not certified by peer review) is the author/funder, who has granted bioRxiv a license to display the preprint in perpetuity. It is made available under a CC-BY 4.0 International license.

Table S17 | **Realized generation and redesign action space in the seven primary T-REX campaigns.** Distinct recorded configuration settings are counted separately for each target and action family; the same settings used for two targets therefore count twice. Confirmed starts exclude standardized AF2 evaluation. Final TM0.6 SUs are attributed to the action family that directly produced each representative after evaluation, qualification and clustering. One start can return multiple designs and can therefore receive credit for more than one SU.

Action family	Targets	Configurations	Confirmed starts	Final TM0.6 SUs
Complexa Beam	7	59	633	869
Complexa FK steering	7	33	462	322
Complexa MCTS	3	22	68	48
Complexa best-of-n	7	15	54	23
BindCraft	7	12	170	191
BoltzGen	7	13	25	3
ProteinMPNN redesign	6	7	42	5
Total	7	161	1,454	1,461

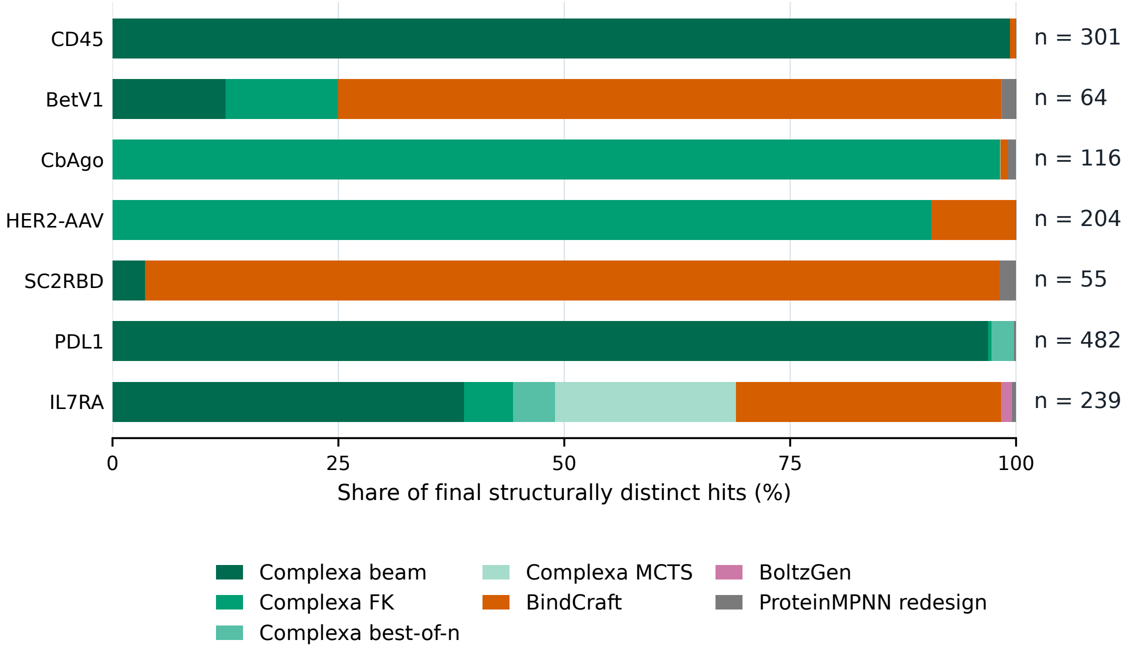

Figure S5 | **Action-family attribution of final T-REX TM0.6 structure-unique units by target.** Each bar partitions the target's final Foldseek TM0.6 clusters according to the generation or redesign family that directly produced the selected representative; labels at right give the total number of structure-unique units (SUs). Each cluster was counted once, and the standardized AF2 job that evaluated an output received no direct credit. A generation or redesign job could produce several designs and could therefore contribute more than one SU. The plot describes output attribution, not causal contribution or per-start efficiency. Supplementary Table S17 reports the corresponding counts pooled across targets.

bioRxiv preprint doi: https://doi.org/10.64898/2026.09.22.753604; this version posted September 24, 2026. The copyright holder for this preprint (which was not certified by peer review) is the author/funder, who has granted bioRxiv a license to display the preprint in perpetuity. It is made available under a CC-BY 4.0 International license.

D.3.3. Intervention without explicit Rescue prioritization

To test explicit **Rescue** prioritization, we ran one intervention campaign per target with a planned budget of 100 worker GPU-h. The action space, structured evidence, resource limits and campaign-state thresholds were retained. Planner and Supervisor priorities were restricted to **Explore** and **eXploit**; fallback allocation and priority accounting also used only these two priorities. Candidates assigned **Rescue** by deterministic rules were relabeled **Explore**. Rescue terminology was removed from LLM instructions, and the near-miss-enriched state was given the neutral label `blocker_rich` without changing its definition.

Recovery and time in stall. Figure 4b uses planning records within the first 100 worker GPU-h of each policy. After retaining the last record at each worker-time value, each transition into stall or deep stall began a new entry, including a transition from stall to deep stall (state definitions in Section C.4.5). Recovery was the first later planning record at which the highest online TM0.6 SU count observed so far exceeded its value at entry, even if the state had changed. Recovery times therefore have planning-record resolution. Entries without recovery by cutoff remained in the recovery-fraction denominator. Recovery-time medians used only recovered entries, without adjusting for unrecovered entries.

Full T-REX recovered in 26 of 27 entries, compared with 14 of 20 under the intervention; one and six entries, respectively, had no observed recovery. These entries are repeated observations within campaigns and can share a recovery observation: the 26 and 14 recovered entries corresponded to 21 and nine distinct target–recovery-time pairs, respectively.

To calculate stall occupancy, each state label was carried to the next planning record, with the last label extended to 100 worker GPU-h. The total duration assigned to stall or deep stall, divided by the summed window durations across targets, gave pooled occupancies of 3.8% for full T-REX and 27.7% for the intervention.

Endpoint comparison. Supplementary Table S18 compares final intervention SU counts with full-T-REX counts from the retrospectively reconstructed accumulation curves in Section D.1.2, rather than the online counts used for recovery. Each full-T-REX curve was read at the worker time reached by its corresponding intervention, capped at 100 worker GPU-h. Full T-REX was planned for 144 worker GPU-h, so this is a comparison at the same observed worker time, not between campaigns run with the same planned budget.

Table S18 | **Intervention removing explicit Rescue priority.** The intervention was planned for 100 H100 worker GPU-h; its columns report final TM0.6 SU counts and rates. Full-T-REX counts come from the reconstructed accumulation curves in Section D.1.2, read at the corresponding observed worker time, from campaigns planned for 144 H100 worker GPU-h. Both rates divide by the intervention's observed worker time, using unrounded values. Each target has one campaign per condition; the comparison is descriptive.

		Rescue priority removed		Full T-REX checkpoint	
Target	Observed GPU-h	SU	SU/GPU-h	SU	SU/GPU-h
CD45	99.742	225	2.256	231	2.316
BetV1	99.991	56	0.560	43	0.430
CbAgo	99.988	49	0.490	86	0.860
HER2-AAV	99.977	181	1.810	144	1.440
SC2RBD	99.968	2	0.020	43	0.430
PDL1	99.963	311	3.111	378	3.781
IL7RA	99.951	235	2.351	182	1.821

Full T-REX had higher counts at this checkpoint for four targets and lower counts for three. The

bioRxiv preprint doi: https://doi.org/10.64898/2026.09.22.753604; this version posted September 24, 2026. The copyright holder for this preprint (which was not certified by peer review) is the author/funder, who has granted bioRxiv a license to display the preprint in perpetuity. It is made available under a CC-BY 4.0 International license.

comparison thus does not establish a uniform throughput benefit. Removing **Rescue** could also change how often stalls occurred and which stalls entered the recovery analysis. The intervention jointly changed LLM instructions, priority allocation and state-label wording; it does not isolate an allocation coefficient or the contribution of LLM reasoning.

D.4. Post-hoc characterization of binder designs

D.4.1. Additional prediction models and metrics

We characterized the fixed representatives defined in Section B.2 using additional model predictions and scores. The set contained 3,766 representatives across six methods and seven targets, with one representative per final Foldseek TM0.6 cluster within each method–target pair. All had finite results for every measurement in Table S19. Harmonized AF2 re-evaluated complexes with common settings, and actifpTM used the same AF2-Multimer configuration (Evans et al., 2021; Varga et al., 2025). Both therefore used the same predictor family as qualification. ESMFold2 and Boltz-2 instead re-predicted complexes from target and binder sequences alone (Candido et al., 2026; Passaro et al., 2025). These post-hoc analyses did not guide allocation or change qualification, clustering or SU counts.

The AF2-derived interaction prediction score from aligned errors (ipSAE) is a higher-is-better interface-confidence score, distinct from the lower-is-better normalized iPAE used for qualification (Dunbrack Jr, 2025). We report the arithmetic mean of its two directional values; actifpTM weights interface confidence by predicted contact probabilities. PRODIGY supplied predicted binding free energy ΔG and the dissociation constant K_{D} derived from it (Xue et al., 2016). These are predictions of confidence or affinity, rather than experimental binding measurements. Table S19 gives evaluator inputs, settings, units and score aggregation.

Figure 2c shows selected post-hoc thresholds; Supplementary Figure S6a–d reports all 24 thresholds across harmonized AF2, actifpTM, PRODIGY, ESMFold2 and Boltz-2. Each threshold was applied separately to the same fixed representative set, and passing counts were pooled across the seven targets. T-REX retained the largest pooled count at every threshold. These pooled counts reflect both the size of each method's qualified representative pool and the fraction passing each threshold. The thresholds summarize related measurements, including ΔG and its derived K_{D}, and are not independent validation experiments.

bioRxiv preprint doi: https://doi.org/10.64898/2026.09.22.753604; this version posted September 24, 2026. The copyright holder for this preprint (which was not certified by peer review) is the author/funder, who has granted bioRxiv a license to display the preprint in perpetuity. It is made available under a CC-BY 4.0 International license.

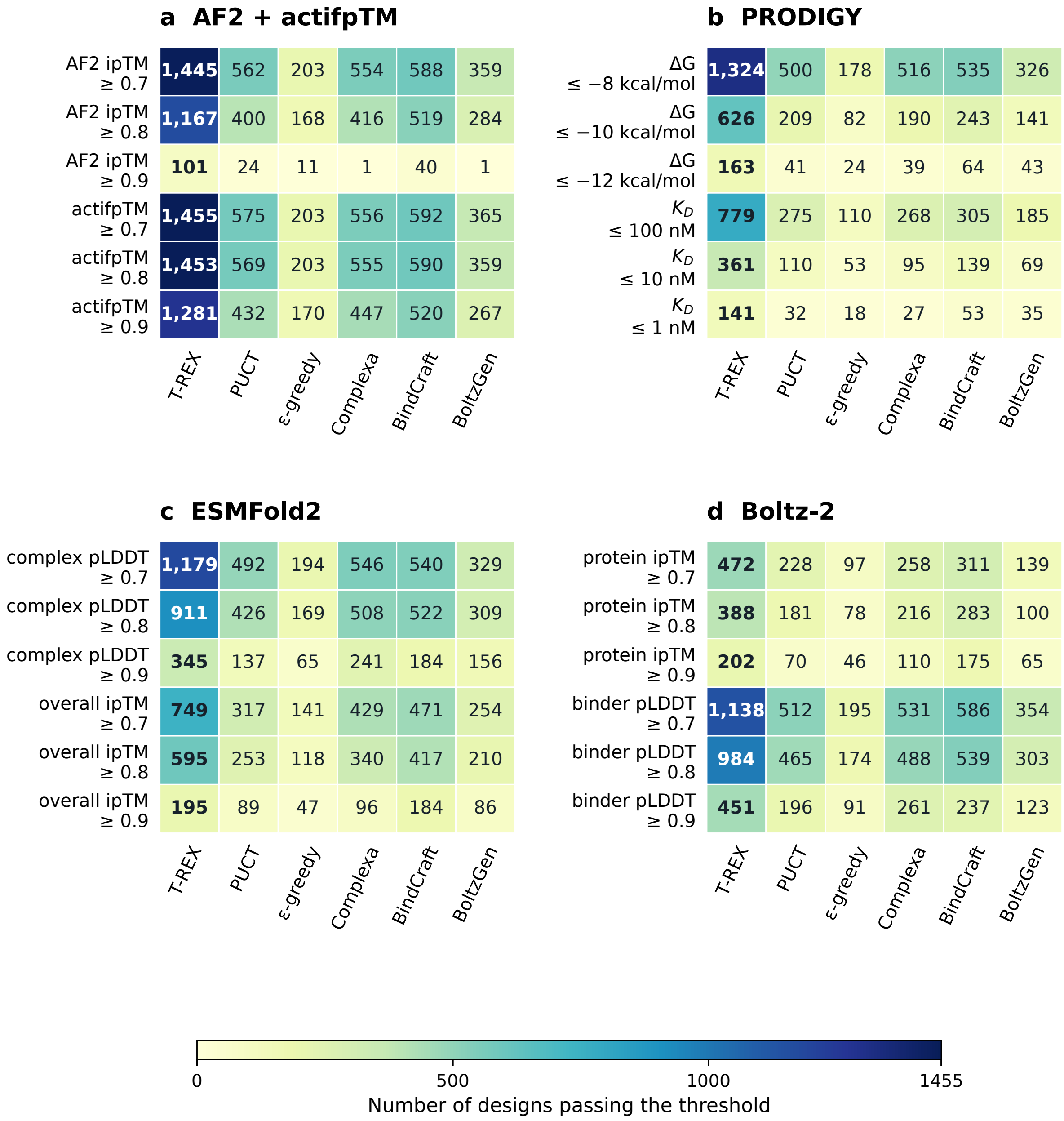

Figure S6 | **Fixed cluster representatives retained across post-hoc evaluation thresholds.** Counts of fixed representatives from final Foldseek TM0.6 clusters passing thresholds for **a**, harmonized AF2 ipTM and actifpTM; **b**, PRODIGY predicted ΔG (kcal mol^{-1}) and derived K_{D} (nM); **c**, ESMFold2 complex mean pLDDT and package-reported overall ipTM; and **d**, Boltz-2 protein ipTM and binder pLDDT. ESMFold2 and Boltz-2 pLDDT thresholds use the reported 0–1 scale. Counts are pooled across seven targets. Each threshold was applied separately to the same representative set. All four panels share one count scale; bold values mark the largest count at each threshold. Complexa, BindCraft and BoltzGen denote generator-only baselines. These post-hoc diagnostics did not affect allocation, qualification or SU counting.

bioRxiv preprint doi: https://doi.org/10.64898/2026.09.22.753604; this version posted September 24, 2026. The copyright holder for this preprint (which was not certified by peer review) is the author/funder, who has granted bioRxiv a license to display the preprint in perpetuity. It is made available under a CC-BY 4.0 International license.

Table S19 | **Post-hoc evaluator inputs, settings and outputs.** All 3,766 fixed representatives across six methods and seven targets had finite results for every listed measurement. Software versions and model revisions are listed in Table S4.

Evaluator	Input and settings	Reported measurements
Harmonized AF2	Representative complex (original qualification complex when recorded; otherwise representative PDB), with the common AF2 qualification settings in Table S4 and a fixed input sequence. The effective seed and AF2 weight hash were not stored per result.	Interface predicted template-modeling score (ipTM) and arithmetic mean of the two directional ipSAE values. The latter differs from the maximum over directions reported by the original ipSAE method.
actifpTM	Same representative complex and AF2-Multimer configuration as harmonized AF2; calculated from AF2 outputs by the study implementation.	Interface pTM weighted by AF2-predicted probabilities of inter-chain distances below 8 Å (from the distogram). The reported score uses probability weights, not the binary contact variant.
PRODIGY	Harmonized AF2-Multimer predicted complex; target/binder chain selection and package defaults, without parameter overrides.	Predicted ΔG (kcal mol^{-1}) and derived K_{D} (M), using the default 25 °C conversion. The two values are monotonically related.
ESMFold2	Target and binder sequences only; 20 loops, 100 sampling steps, one diffusion sample and seed equal to the representative's fixed integer evaluation index.	Package-reported overall ipTM and mean per-residue pLDDT across the complex (0–1 scale). The symmetric mean of target-to-binder and binder-to-target pair-chain ipTM entries was retained as an auxiliary measurement; Figure S6c and the shortlists in Section D.4.2 use overall ipTM.
Boltz-2	Target and binder sequences only; empty multiple-sequence-alignment (MSA) mode, 200 sampling steps, three recycling steps, one diffusion sample and full PAE output. The launcher did not set an explicit random seed.	Package-reported protein ipTM, mean PAE over all target–binder residue pairs averaged across both directions (Å), and binder mean pLDDT (0–1 scale). The affinity head was not used to assess these protein–protein candidates.

D.4.2. Metric-specific shortlists

Tables S20–S22 summarize fixed TM0.6 representatives from each method–target pair under six separate rankings: minimum qualification margin, AF2 ipTM, mean directional ipSAE, ESMFold2 overall ipTM, actifpTM and PRODIGY predicted affinity. Each ranking selects its own shortlist; ΔG and K_{D} describe the same affinity-ranked shortlist. From N available representatives, each list retains $n = \min(10, N)$ without padding. Margins are defined in Section B.2, and post-hoc metrics in Section D.4.1. These summaries describe metric-specific selection from pools of different sizes, not one common multi-metric shortlist. Because the ranking scores also summarize the selected designs, this is not independent validation.

Selection and tie-breaking. The margin ranking orders designs by decreasing $m_{\min}$, then mean margin, then pLDDT, iPAE and scRMSD margins, in that order. Each confidence ranking uses decreasing values of its named score, then $m_{\min}$ and mean margin. The affinity ranking uses increasing K_{D}, then ΔG, followed by decreasing $m_{\min}$ and mean margin. Remaining ties are resolved by ascending lexicographic order of the stable record identifier. All rankings use values before display rounding.

bioRxiv preprint doi: https://doi.org/10.64898/2026.09.22.753604; this version posted September 24, 2026. The copyright holder for this preprint (which was not certified by peer review) is the author/funder, who has granted bioRxiv a license to display the preprint in perpetuity. It is made available under a CC-BY 4.0 International license.

Table S20 | **Qualification-margin and AF2-confidence shortlists.** Each metric selects its own top $n = \min(10, N)$ representatives from the method–target pool of size N and reports median [best, worst]; larger values rank higher. Within each target and metric, bold and underline mark the highest and second-highest distinct unrounded medians among lists of ten designs; ties share a mark. Identical rounded values can carry different marks. Shorter lists carry a dagger and are excluded from highlighting; – denotes an empty pool. Selection rules are given in Section D.4.2.

Target	Method	N	$n/10$	Minimum margin $m_{\min}$	AF2 ipTM	AF2 mean directional ipSAE
CD45	T-REX	301	10/10	**0.359** [0.395, 0.328]	**0.872** [0.887, 0.865]	**0.714** [0.729, 0.711]
CD45	PUCT	69	10/10	0.225 [0.364, 0.199]	0.824 [0.876, 0.819]	0.622 [0.715, 0.594]
CD45	ϵ-greedy	27	10/10	<u>0.289</u> [0.497, 0.224]	<u>0.838</u> [0.880, 0.832]	<u>0.648</u> [0.746, 0.589]
CD45	Complexa-only	50	10/10	0.172 [0.241, 0.134]	0.809 [0.845, 0.801]	0.599 [0.676, 0.567]
CD45	BindCraft-only	2	2/10†	0.216 [0.321, 0.111]	0.811 [0.837, 0.784]	0.585 [0.639, 0.531]
CD45	BoltzGen-only	5	5/10†	0.170 [0.326, 0.020]	0.780 [0.856, 0.722]	0.528 [0.665, 0.436]
BetV1	T-REX	64	10/10	**0.351** [0.438, 0.330]	**0.898** [0.915, 0.887]	**0.793** [0.823, 0.765]
BetV1	PUCT	34	10/10	<u>0.345</u> [0.415, 0.296]	<u>0.896</u> [0.912, 0.882]	<u>0.784</u> [0.817, 0.752]
BetV1	ϵ-greedy	17	10/10	0.279 [0.404, 0.161]	0.867 [0.905, 0.837]	0.707 [0.817, 0.650]
BetV1	Complexa-only	17	10/10	0.113 [0.243, 0.038]	0.816 [0.838, 0.789]	0.588 [0.671, 0.532]
BetV1	BindCraft-only	3	3/10†	0.106 [0.173, 0.069]	0.838 [0.862, 0.780]	0.610 [0.694, 0.555]
BetV1	BoltzGen-only	15	10/10	0.186 [0.363, 0.070]	0.867 [0.898, 0.852]	0.675 [0.774, 0.615]
CbAgo	T-REX	116	10/10	**0.382** [0.412, 0.332]	<u>0.862</u> [0.888, 0.853]	<u>0.682</u> [0.726, 0.659]
CbAgo	PUCT	54	10/10	0.306 [0.424, 0.287]	**0.868** [0.872, 0.859]	**0.688** [0.711, 0.645]
CbAgo	ϵ-greedy	9	9/10†	0.256 [0.498, 0.019]	0.847 [0.890, 0.746]	0.633 [0.778, 0.437]
CbAgo	Complexa-only	2	2/10†	0.019 [0.030, 0.008]	0.760 [0.767, 0.754]	0.490 [0.495, 0.486]
CbAgo	BindCraft-only	52	10/10	0.298 [0.416, 0.250]	0.855 [0.890, 0.840]	0.654 [0.750, 0.615]
CbAgo	BoltzGen-only	37	10/10	<u>0.307</u> [0.415, 0.280]	0.841 [0.854, 0.832]	0.645 [0.695, 0.604]

Continued on next page

bioRxiv preprint doi: https://doi.org/10.64898/2026.09.22.753604; this version posted September 24, 2026. The copyright holder for this preprint (which was not certified by peer review) is the author/funder, who has granted bioRxiv a license to display the preprint in perpetuity. It is made available under a CC-BY 4.0 International license.

Supplementary Table S20 continued

Target	Method	N	$n/10$	Minimum margin $m_{\min}$	AF2 ipTM	AF2 mean directional ipSAE
HER2-AAV	T-REX	204	10/10	0.182 [0.228, 0.167]	0.921 [0.924, 0.919]	**0.754** [0.771, 0.744]
HER2-AAV	PUCT	52	10/10	0.180 [0.235, 0.151]	0.909 [0.928, 0.908]	0.740 [0.767, 0.721]
HER2-AAV	ϵ-greedy	27	10/10	0.154 [0.243, 0.114]	0.906 [0.918, 0.901]	0.722 [0.755, 0.701]
HER2-AAV	Complexa-only	42	10/10	0.071 [0.150, 0.057]	0.890 [0.902, 0.886]	0.671 [0.713, 0.652]
HER2-AAV	BindCraft-only	104	10/10	**0.190** [0.211, 0.178]	**0.923** [0.929, 0.919]	0.739 [0.776, 0.733]
HER2-AAV	BoltzGen-only	2	$2/10^{\dagger}$	0.027 [0.040, 0.014]	0.855 [0.875, 0.835]	0.544 [0.587, 0.501]
SC2RBD	T-REX	55	10/10	0.301 [0.387, 0.267]	0.873 [0.889, 0.864]	0.732 [0.773, 0.712]
SC2RBD	PUCT	40	10/10	**0.301** [0.361, 0.279]	**0.881** [0.899, 0.867]	0.742 [0.776, 0.729]
SC2RBD	ϵ-greedy	20	10/10	0.286 [0.342, 0.203]	0.870 [0.884, 0.844]	0.724 [0.770, 0.674]
SC2RBD	Complexa-only	0	$0/10^{\dagger}$	–	–	–
SC2RBD	BindCraft-only	22	10/10	0.291 [0.332, 0.275]	0.879 [0.892, 0.870]	**0.747** [0.784, 0.714]
SC2RBD	BoltzGen-only	6	$6/10^{\dagger}$	0.127 [0.231, 0.032]	0.834 [0.860, 0.824]	0.601 [0.675, 0.576]
PDL1	T-REX	482	10/10	0.495 [0.525, 0.489]	**0.896** [0.904, 0.892]	**0.788** [0.795, 0.783]
PDL1	PUCT	233	10/10	**0.501** [0.538, 0.482]	0.891 [0.902, 0.888]	0.761 [0.790, 0.753]
PDL1	ϵ-greedy	65	10/10	0.421 [0.503, 0.394]	0.883 [0.895, 0.875]	0.757 [0.791, 0.741]
PDL1	Complexa-only	281	10/10	0.446 [0.486, 0.410]	0.870 [0.884, 0.865]	0.722 [0.762, 0.715]
PDL1	BindCraft-only	231	10/10	0.481 [0.507, 0.466]	0.894 [0.902, 0.890]	0.785 [0.803, 0.777]
PDL1	BoltzGen-only	158	10/10	0.500 [0.540, 0.484]	0.888 [0.900, 0.885]	0.759 [0.796, 0.743]
IL7RA	T-REX	239	10/10	**0.294** [0.377, 0.272]	0.881 [0.904, 0.878]	**0.727** [0.770, 0.723]
IL7RA	PUCT	95	10/10	0.265 [0.380, 0.246]	0.876 [0.910, 0.874]	0.710 [0.732, 0.700]
IL7RA	ϵ-greedy	40	10/10	0.190 [0.251, 0.167]	0.864 [0.882, 0.847]	0.671 [0.711, 0.644]
IL7RA	Complexa-only	167	10/10	0.230 [0.277, 0.205]	0.869 [0.879, 0.862]	0.692 [0.714, 0.675]
IL7RA	BindCraft-only	178	10/10	0.282 [0.308, 0.261]	**0.884** [0.899, 0.875]	0.722 [0.755, 0.713]
IL7RA	BoltzGen-only	149	10/10	0.205 [0.306, 0.191]	0.873 [0.907, 0.866]	0.646 [0.695, 0.633]

bioRxiv preprint doi: https://doi.org/10.64898/2026.09.22.753604; this version posted September 24, 2026. The copyright holder for this preprint (which was not certified by peer review) is the author/funder, who has granted bioRxiv a license to display the preprint in perpetuity. It is made available under a CC-BY 4.0 International license.

Table S21 | **ESMFold2 and actifpTM confidence shortlists.** Each metric selects its own top $n = \min(10, N)$ representatives from the method–target pool of size N and reports median [best, worst]; larger values rank higher. ESMFold2 uses the package-reported overall ipTM. Within each target and metric, bold and underline mark the highest and second-highest distinct unrounded medians among lists of ten designs; ties share a mark. Identical rounded values can carry different marks. Shorter lists carry a dagger and are excluded from highlighting; – denotes an empty pool. Selection rules are given in Section D.4.2.

Target	Method	N	$n/10$	ESMFold2 overall ipTM	actifpTM
CD45	T-REX	301	10/10	**0.813** [0.906, 0.778]	**0.959** [0.974, 0.958]
CD45	PUCT	69	10/10	0.716 [0.827, 0.685]	<u>0.946</u> [0.961, 0.934]
CD45	ϵ-greedy	27	10/10	0.733 [0.819, 0.610]	0.941 [0.958, 0.919]
CD45	Complexa-only	50	10/10	<u>0.744</u> [0.864, 0.699]	0.933 [0.954, 0.931]
CD45	BindCraft-only	2	2/10†	0.542 [0.779, 0.305]	0.896 [0.924, 0.868]
CD45	BoltzGen-only	5	5/10†	0.207 [0.528, 0.082]	0.924 [0.937, 0.858]
BetV1	T-REX	64	10/10	<u>0.932</u> [0.955, 0.920]	<u>0.965</u> [0.978, 0.957]
BetV1	PUCT	34	10/10	**0.932** [0.951, 0.927]	**0.966** [0.971, 0.955]
BetV1	ϵ-greedy	17	10/10	0.897 [0.946, 0.847]	0.944 [0.962, 0.922]
BetV1	Complexa-only	17	10/10	0.868 [0.917, 0.644]	0.910 [0.927, 0.898]
BetV1	BindCraft-only	3	3/10†	0.786 [0.919, 0.648]	0.927 [0.946, 0.906]
BetV1	BoltzGen-only	15	10/10	0.858 [0.921, 0.814]	0.943 [0.963, 0.923]
CbAgo	T-REX	116	10/10	<u>0.353</u> [0.636, 0.231]	**0.943** [0.952, 0.936]
CbAgo	PUCT	54	10/10	**0.360** [0.714, 0.248]	0.940 [0.951, 0.932]
CbAgo	ϵ-greedy	9	9/10†	0.107 [0.301, 0.072]	0.919 [0.947, 0.842]
CbAgo	Complexa-only	2	2/10†	0.201 [0.317, 0.086]	0.902 [0.911, 0.893]
CbAgo	BindCraft-only	52	10/10	0.242 [0.722, 0.206]	<u>0.941</u> [0.961, 0.925]
CbAgo	BoltzGen-only	37	10/10	0.140 [0.441, 0.118]	0.927 [0.952, 0.914]

Continued on next page

bioRxiv preprint doi: https://doi.org/10.64898/2026.09.22.753604; this version posted September 24, 2026. The copyright holder for this preprint (which was not certified by peer review) is the author/funder, who has granted bioRxiv a license to display the preprint in perpetuity. It is made available under a CC-BY 4.0 International license.

Supplementary Table S21 continued

Target	Method	N	$n/10$	ESMFold2 overall ipTM	actifpTM
HER2-AAV	T-REX	204	10/10	0.942 [0.958, 0.933]	0.973 [0.976, 0.972]
HER2-AAV	PUCT	52	10/10	0.942 [0.953, 0.937]	0.972 [0.976, 0.968]
HER2-AAV	ϵ-greedy	27	10/10	0.915 [0.947, 0.906]	0.970 [0.973, 0.967]
HER2-AAV	Complexa-only	42	10/10	0.937 [0.956, 0.926]	0.956 [0.961, 0.949]
HER2-AAV	BindCraft-only	104	10/10	**0.953** [0.961, 0.944]	**0.975** [0.977, 0.973]
HER2-AAV	BoltzGen-only	2	2/10†	0.515 [0.934, 0.096]	0.924 [0.943, 0.905]
SC2RBD	T-REX	55	10/10	0.906 [0.922, 0.888]	**0.956** [0.968, 0.943]
SC2RBD	PUCT	40	10/10	0.895 [0.914, 0.884]	0.956 [0.967, 0.948]
SC2RBD	ϵ-greedy	20	10/10	0.868 [0.934, 0.771]	0.950 [0.960, 0.935]
SC2RBD	Complexa-only	0	0/10†	–	–
SC2RBD	BindCraft-only	22	10/10	**0.910** [0.928, 0.816]	0.950 [0.964, 0.947]
SC2RBD	BoltzGen-only	6	6/10†	0.789 [0.862, 0.096]	0.909 [0.931, 0.889]
PDL1	T-REX	482	10/10	0.943 [0.955, 0.937]	**0.961** [0.967, 0.960]
PDL1	PUCT	233	10/10	0.928 [0.940, 0.925]	0.956 [0.959, 0.953]
PDL1	ϵ-greedy	65	10/10	0.930 [0.942, 0.921]	0.954 [0.957, 0.952]
PDL1	Complexa-only	281	10/10	0.934 [0.943, 0.929]	0.952 [0.956, 0.951]
PDL1	BindCraft-only	231	10/10	**0.946** [0.951, 0.939]	0.959 [0.961, 0.958]
PDL1	BoltzGen-only	158	10/10	0.941 [0.949, 0.933]	0.951 [0.960, 0.948]
IL7RA	T-REX	239	10/10	0.922 [0.945, 0.906]	0.957 [0.966, 0.956]
IL7RA	PUCT	95	10/10	0.927 [0.954, 0.914]	0.955 [0.964, 0.954]
IL7RA	ϵ-greedy	40	10/10	0.891 [0.925, 0.878]	0.944 [0.947, 0.939]
IL7RA	Complexa-only	167	10/10	0.922 [0.950, 0.909]	0.951 [0.957, 0.948]
IL7RA	BindCraft-only	178	10/10	0.930 [0.945, 0.923]	**0.960** [0.969, 0.957]
IL7RA	BoltzGen-only	149	10/10	**0.933** [0.955, 0.926]	0.947 [0.959, 0.945]

bioRxiv preprint doi: https://doi.org/10.64898/2026.09.22.753604; this version posted September 24, 2026. The copyright holder for this preprint (which was not certified by peer review) is the author/funder, who has granted bioRxiv a license to display the preprint in perpetuity. It is made available under a CC-BY 4.0 International license.

Table S22 | **PRODIGY predicted-affinity shortlists.** ΔG and K_D report median [best, worst] for the same K_D-ranked top $n = \min(10, N)$ representatives from the method–target pool of size N; smaller values rank higher. These are predicted affinities. Within each target and metric, bold and underline mark the lowest and second-lowest distinct unrounded medians among lists of ten designs; ties share a mark. Identical rounded values can carry different marks. Shorter lists carry a dagger and are excluded from highlighting; – denotes an empty pool. Selection rules are given in Section D.4.2.

Target	Method	N	$n/10$	PRODIGY ΔG (kcal mol^{-1})	PRODIGY K_D (M)
CD45	T-REX	301	10/10	**-12.90**<br>[-13.40, -12.00]	**3.6e-10**<br>[1.5e-10, 1.5e-09]
CD45	PUCT	69	10/10	-11.50<br>[-13.00, -10.90]	3.7e-09<br>[3.0e-10, 1.0e-08]
CD45	ϵ-greedy	27	10/10	-9.95<br>[-13.70, -9.30]	5.2e-08<br>[9.1e-11, 1.4e-07]
CD45	Complexa-only	50	10/10	-12.05<br>[-12.80, -11.30]	1.4e-09<br>[3.9e-10, 4.9e-09]
CD45	BindCraft-only	2	2/10†	-9.90<br>[-10.30, -9.50]	6.8e-08<br>[2.7e-08, 1.1e-07]
CD45	BoltzGen-only	5	5/10†	-11.00<br>[-12.20, -9.70]	8.7e-09<br>[1.1e-09, 8.3e-08]
BetV1	T-REX	64	10/10	-10.90<br>[-13.80, -10.30]	1.1e-08<br>[7.4e-11, 2.7e-08]
BetV1	PUCT	34	10/10	-11.50<br>[-14.10, -10.30]	3.7e-09<br>[4.8e-11, 2.6e-08]
BetV1	ϵ-greedy	17	10/10	-9.75<br>[-12.40, -8.50]	7.0e-08<br>[8.1e-10, 5.7e-07]
BetV1	Complexa-only	17	10/10	-8.35<br>[-9.30, -7.80]	7.5e-07<br>[1.6e-07, 1.8e-06]
BetV1	BindCraft-only	3	3/10†	-8.60<br>[-12.60, -6.00]	5.1e-07<br>[5.5e-10, 4.0e-05]
BetV1	BoltzGen-only	15	10/10	**-13.00**<br>[-17.80, -11.40]	**3.1e-10**<br>[9.0e-14, 4.6e-09]
CbAgo	T-REX	116	10/10	-11.05<br>[-12.70, -10.70]	7.5e-09<br>[4.7e-10, 1.5e-08]
CbAgo	PUCT	54	10/10	-10.65<br>[-11.70, -10.40]	1.5e-08<br>[2.7e-09, 2.5e-08]
CbAgo	ϵ-greedy	9	9/10†	-9.70<br>[-11.90, -8.70]	7.1e-08<br>[2.0e-09, 4.2e-07]
CbAgo	Complexa-only	2	2/10†	-9.10<br>[-9.40, -8.80]	2.4e-07<br>[1.3e-07, 3.5e-07]
CbAgo	BindCraft-only	52	10/10	-10.90<br>[-12.80, -10.60]	9.7e-09<br>[4.0e-10, 1.8e-08]
CbAgo	BoltzGen-only	37	10/10	**-13.60**<br>[-16.10, -13.30]	**9.9e-11**<br>[1.5e-12, 1.9e-10]

Continued on next page

bioRxiv preprint doi: https://doi.org/10.64898/2026.09.22.753604; this version posted September 24, 2026. The copyright holder for this preprint (which was not certified by peer review) is the author/funder, who has granted bioRxiv a license to display the preprint in perpetuity. It is made available under a CC-BY 4.0 International license.

Supplementary Table S22 continued

Target	Method	N	$n/10$	PRODIGY ΔG (kcal mol^{-1})	PRODIGY K_D (M)
HER2-AAV	T-REX	204	10/10	**-15.95**<br>[-17.10, -15.20]	**2.2e-12**<br>[2.9e-13, 7.5e-12]
HER2-AAV	PUCT	52	10/10	-13.55<br>[-15.00, -12.70]	1.2e-10<br>[9.4e-12, 4.8e-10]
HER2-AAV	ϵ-greedy	27	10/10	-12.60<br>[-15.60, -12.00]	5.6e-10<br>[3.5e-12, 1.6e-09]
HER2-AAV	Complexa-only	42	10/10	-13.75<br>[-15.70, -13.10]	9.4e-11<br>[2.9e-12, 2.4e-10]
HER2-AAV	BindCraft-only	104	10/10	-14.50<br>[-15.40, -13.70]	2.3e-11<br>[5.3e-12, 8.7e-11]
HER2-AAV	BoltzGen-only	2	2/10†	-10.30<br>[-11.40, -9.20]	8.7e-08<br>[4.7e-09, 1.7e-07]
SC2RBD	T-REX	55	10/10	-12.30<br>[-13.60, -11.90]	9.1e-10<br>[1.1e-10, 1.7e-09]
SC2RBD	PUCT	40	10/10	**-12.85**<br>[-16.20, -12.20]	**4.0e-10**<br>[1.3e-12, 1.1e-09]
SC2RBD	ϵ-greedy	20	10/10	-12.25<br>[-13.60, -11.00]	1.1e-09<br>[1.1e-10, 9.3e-09]
SC2RBD	Complexa-only	0	0/10†	–	–
SC2RBD	BindCraft-only	22	10/10	-12.25<br>[-13.90, -11.60]	1.1e-09<br>[6.6e-11, 3.3e-09]
SC2RBD	BoltzGen-only	6	6/10†	-12.30<br>[-13.80, -10.60]	1.0e-09<br>[7.2e-11, 1.7e-08]
PDL1	T-REX	482	10/10	**-13.15**<br>[-14.40, -12.70]	**2.4e-10**<br>[2.7e-11, 5.1e-10]
PDL1	PUCT	233	10/10	-11.65<br>[-12.70, -11.20]	2.8e-09<br>[4.5e-10, 5.8e-09]
PDL1	ϵ-greedy	65	10/10	-11.50<br>[-12.70, -10.70]	3.7e-09<br>[4.8e-10, 1.5e-08]
PDL1	Complexa-only	281	10/10	-12.00<br>[-12.90, -11.70]	1.5e-09<br>[3.6e-10, 2.5e-09]
PDL1	BindCraft-only	231	10/10	-11.80<br>[-12.40, -11.40]	2.2e-09<br>[8.1e-10, 4.1e-09]
PDL1	BoltzGen-only	158	10/10	-12.35<br>[-14.50, -11.10]	9.0e-10<br>[2.3e-11, 7.6e-09]
IL7RA	T-REX	239	10/10	-11.45<br>[-12.70, -11.30]	4.0e-09<br>[4.5e-10, 5.1e-09]
IL7RA	PUCT	95	10/10	-11.15<br>[-13.50, -10.80]	7.1e-09<br>[1.2e-10, 1.2e-08]
IL7RA	ϵ-greedy	40	10/10	-10.85<br>[-12.60, -9.70]	1.1e-08<br>[5.9e-10, 7.8e-08]
IL7RA	Complexa-only	167	10/10	-11.25<br>[-12.70, -11.00]	5.5e-09<br>[4.7e-10, 9.2e-09]
IL7RA	BindCraft-only	178	10/10	-11.45<br>[-12.60, -10.90]	4.2e-09<br>[5.8e-10, 1.0e-08]
IL7RA	BoltzGen-only	149	10/10	**-11.55**<br>[-13.30, -10.90]	**3.3e-09**<br>[1.8e-10, 1.0e-08]

D.4.3. Secondary structure and binding-position diversity

Secondary-structure annotation and examples. For Figure 5a,b, we annotated binder secondary structure in the 1,461 final T-REX TM0.6 representatives with MDTraj 1.11.0 (`compute_dssp`, `simplified=True`) (Kabsch and Sander, 1983; McGibbon et al., 2015). DSSP states H, G and

bioRxiv preprint doi: https://doi.org/10.64898/2026.09.22.753604; this version posted September 24, 2026. The copyright holder for this preprint (which was not certified by peer review) is the author/funder, who has granted bioRxiv a license to display the preprint in perpetuity. It is made available under a CC-BY 4.0 International license.

I count as helix, and E and B as strand; each fraction uses all binder residues as its denominator, and remaining assignments count as other. The helix-rich and β-rich examples maximize helix and strand fractions, respectively, with ties favoring the smaller complementary fraction. The mixed helix/strand example maximizes the smaller of the two fractions, with ties favoring their larger sum. Remaining ties use ascending record-identifier order. Molecular views in Figure 5a,c were rendered with UCSF ChimeraX 1.11 (Pettersen et al., 2021).

To compare β-rich output across methods, we selected all qualified designs with at least 20% strand before Foldseek TM0.6 clustering within each method–target pool. This counts clusters within the β-rich subset, rather than β-rich members of the fixed representative set. T-REX was the only method with such designs on all seven targets and yielded 308 clusters (0.306 per worker GPU-h), compared with 95 (0.094 per worker GPU-h) for BoltzGen-only, the next-highest method (Supplementary Table S23). T-REX yielded more clusters than PUCT on six targets and more than the highest-yielding generator-only method for each target on five. These annotations did not change the primary endpoint.

Table S23 | **β-rich qualified designs and structural clusters by target.** A binder is β-rich when at least 20% of its residues are assigned to the strand category by MDTraj's simplified three-state DSSP implementation. Entries give qualified β-rich designs / TM0.6 clusters after reclustering that subset within each method–target pool. The final row gives pooled counts across the seven targets; pooled rates in the text use the fixed reporting budget of $7 \times 144 = 1{,}008$ worker GPU-h per method. These post-hoc annotations did not affect allocation, qualification or SU credit.

Target	T-REX	PUCT	ϵ-greedy	Complexa only	BindCraft only	BoltzGen only
Entries: qualified β-rich designs / TM0.6 clusters						
CD45	89 / 24	3 / 1	1 / 1	21 / 7	0 / 0	21 / 4
BetV1	22 / 8	0 / 0	0 / 0	13 / 4	0 / 0	5 / 4
CbAgo	38 / 12	6 / 3	0 / 0	0 / 0	2 / 1	61 / 14
HER2-AAV	637 / 167	95 / 17	35 / 8	148 / 40	107 / 25	0 / 0
SC2RBD	5 / 2	4 / 3	2 / 1	0 / 0	2 / 1	1 / 1
PDL1	1,495 / 90	238 / 32	27 / 3	479 / 24	46 / 5	313 / 65
IL7RA	15 / 5	4 / 1	23 / 3	6 / 3	4 / 3	23 / 7
All seven	2,301 / 308	350 / 57	88 / 16	667 / 78	161 / 35	424 / 95

Contact-pattern dissimilarity. To compare contact patterns, we defined a target–binder residue contact as any heavy-atom pair within 6 Å. Each contact feature combined target position with the contacting binder residue's class: hydrophobic, A/V/L/I/M/C; aromatic, F/Y/W; polar, S/T/N/Q/H; positive, K/R; negative, D/E; glycine, G; proline, P; or other. Repeated contacts with the same position–class pair contributed one element to the design's feature set. For two nonempty sets F_i and F_j, dissimilarity was $1 - |F_i \cap F_j|/|F_i \cup F_j|$, ranging from zero for identical patterns to one for no shared features. CbAgo example selection used target residue number and identity to identify positions; the all-target comparison used the sequence alignment described below.

Target-relative binder positions. For Figure 5c, we first aligned predicted CbAgo complexes to a common target reference. Alignment used target Cα atoms shared with the reference at residues contacting a binder in at least 20% of the pooled T-REX and PUCT complexes, with at least 15 matched atoms per complex. The Kabsch rotation and translation were also applied to the binder, whose center was the mean position of its Cα atoms. PCA was fitted jointly to these three-dimensional centers for all final CbAgo T-REX ($n = 116$) and PUCT ($n = 54$) TM0.6 representatives. The plotted

bioRxiv preprint doi: https://doi.org/10.64898/2026.09.22.753604; this version posted September 24, 2026. The copyright holder for this preprint (which was not certified by peer review) is the author/funder, who has granted bioRxiv a license to display the preprint in perpetuity. It is made available under a CC-BY 4.0 International license.

coordinates describe position relative to CbAgo and retain units of Å; axis directions and signs are arbitrary. They do not measure binder fold, orientation or contact-pattern dissimilarity.

To illustrate positional and contact variation among higher-scoring representatives, we selected P1–P3 from the 116 T-REX representatives ranked by the minimum of three scaled margins: $(\mathrm{pLDDT} - 90)/10$, $(7/31 - \mathrm{iPAE})/0.05$ and $(1.5 - \mathrm{scRMSD})/0.3$, with scRMSD in Å. This example-selection score differs from $m_{\min}$ used to choose the final cluster representatives (Section B.2). We retained the upper half ($n = 58$) and chose the highest-scoring design as P1. P2 and P3 each maximized the minimum combined distance to the previously chosen examples: one-half the three-dimensional binder-center separation divided by its 95th percentile across all CbAgo T-REX pairs, plus one-half the contact-pattern dissimilarity.

Sample-size-matched comparisons. For the all-target comparison, we compared contact patterns at a common sample size and mapped target positions by global sequence alignment to remove PDB residue-number offsets. For each target, the reference was the longest sequence observed across T-REX and PUCT, preferring the most frequent at equal length. Alignment scores were $+2$ per match, -1 per mismatch and -2 per gap; ties preferred a diagonal step, then a gap in the query, then a gap in the reference. Only identical aligned residues supplied position mappings. These matches had to cover at least 70% of each sequence and constitute at least 90% of aligned nongap pairs. Contacts without a mapped target position were omitted; every retained design had a nonempty feature set. The resampled CbAgo position comparison also used this sequence mapping, with Kabsch alignment on all matched target Cα atoms. Figure 5c instead uses the recurrent-contact residues defined above.

For each target and method, we sampled 30 final TM0.6 representatives without replacement and repeated sampling 1,000 times (random seed 20260831); all pools contained at least 30 representatives. Each sample yielded a median pairwise contact-pattern dissimilarity, or, for CbAgo positions, a median three-dimensional Euclidean distance between binder centers. We report the mean and fifth–ninety-fifth percentiles of these resampled medians. The intervals describe variation among subsets of the observed pools, not confidence intervals across independent campaigns.

The CbAgo mean resampled median separation was 31.2 Å [25.2–36.6] for T-REX and 9.97 Å [9.08–11.00] for PUCT. Contact-pattern dissimilarity was higher for T-REX on four of seven targets (Supplementary Table S24). These comparisons support broader predicted binder positions for CbAgo, while the contact-pattern comparison did not favor one method uniformly across targets.

bioRxiv preprint doi: https://doi.org/10.64898/2026.09.22.753604; this version posted September 24, 2026. The copyright holder for this preprint (which was not certified by peer review) is the author/funder, who has granted bioRxiv a license to display the preprint in perpetuity. It is made available under a CC-BY 4.0 International license.

Table S24 | **Sample-size-matched predicted contact-pattern variation.** For each target, 30 final TM0.6 representatives were sampled without replacement from the T-REX and PUCT pools; both pools contained at least 30 representatives. The method columns report the mean [5th–95th percentile] of the median pairwise dissimilarity across 1,000 samples; n is the number of representatives per sample and method. Dissimilarity is one minus Jaccard similarity for 6-Å contact features formed from aligned target-sequence position and contacting-binder residue physicochemical class; higher values indicate more varied predicted contact patterns. Δ is the difference between the two means (T-REX minus PUCT), calculated before rounding. Bold marks the larger unrounded mean in each row. Contact patterns measure a different aspect of structural variation from the binder positions in Figure 5c. Intervals describe variation among subsets of the observed pools, not confidence intervals across independent campaigns.

Target	n	T-REX	PUCT	Δ
CD45	30	0.619 [0.592–0.652]	**0.620 [0.597–0.645]**	-0.001
BetV1	30	**0.936 [0.877–0.961]**	0.806 [0.764–0.848]	+0.131
CbAgo	30	**0.945 [0.907–0.975]**	0.675 [0.661–0.689]	+0.270
HER2-AAV	30	**0.704 [0.691–0.717]**	0.660 [0.648–0.672]	+0.044
SC2RBD	30	**0.729 [0.711–0.750]**	0.717 [0.705–0.729]	+0.012
PDL1	30	0.683 [0.663–0.701]	**0.711 [0.691–0.730]**	-0.028
IL7RA	30	0.693 [0.671–0.713]	**0.710 [0.695–0.728]**	-0.017
